# Atualização da Recomendação para Avaliação da Doença das Artérias Carótidas e Vertebrais pela Ultrassonografia Vascular: DIC, CBR, SBACV – 2023

**DOI:** 10.36660/abc.20230695

**Published:** 2023-11-14

**Authors:** Ana Cristina Lopes Albricker, Claudia Maria Vilas Freire, Simone Nascimento dos Santos, Monica Luiza de Alcantara, Armando Luis Cantisano, Carmen Lucia Lascasas Porto, Salomon Israel do Amaral, Orlando Carlos Glória Veloso, Domingos de Morais, José Aldo Ribeiro Teodoro, Ana Cláudia Gomes Pereira Petisco, Mohamed Hassan Saleh, Marcio Vinícius Lins de Barros, Fanilda Souto Barros, Ana Luiza Dias Valiente Engelhorn, Carlos Alberto Engelhorn, Érica Patrício Nardino, Melissa Andreia de Moares Silva, Luisa Ciucci Biagioni, Adriano José de Souza, Anna Karina Paiva Sarpe, Arthur Curtarelli de Oliveira, Marcelo Rodrigo de Souza Moraes, Miguel José Francisco, Peter Célio Françolin, Carlos Eduardo Rochitte, Rogerio Iquizli, Alair Augusto Sarmet Moreira Damas dos Santos, Valdair Francisco Muglia, Bruno de Lima Naves

**Affiliations:** 1 Centro Universitário de Belo Horizonte Belo Horizonte MG Brasil Centro Universitário de Belo Horizonte (UniBH), Belo Horizonte, MG – Brasil; 2 Instituto Mineiro de Ultrassonografia Belo Horizonte MG Brasil IMEDE – Instituto Mineiro de Ultrassonografia, Belo Horizonte, MG – Brasil; 3 Universidade Federal de Minas Gerais Belo Horizonte MG Brasil Universidade Federal de Minas Gerais (UFMG), Belo Horizonte, MG – Brasil; 4 Empresa Brasileira de Serviços Hospitalares Brasília DF Brasil Empresa Brasileira de Serviços Hospitalares (UBSERH), Brasília, DF – Brasil; 5 Eccos Diagnóstico Cardiovascular Avançado Brasília DF Brasil Eccos Diagnóstico Cardiovascular Avançado, Brasília, DF – Brasil; 6 Hospital Quinta D’Or, Rede D’Or São Luiz Rio de Janeiro RJ Brasil Hospital Quinta D’Or, Rede D’Or São Luiz, Rio de Janeiro, RJ – Brasil; 7 Hospital Barra D’Or Rio de Janeiro RJ Brasil Hospital Barra D’Or Rio de Janeiro, RJ – Brasil; 9 Universidade do Estado do Rio de Janeiro Rio de Janeiro RJ Brasil Universidade do Estado do Rio de Janeiro (UERJ), Rio de Janeiro, RJ – Brasil; 10 Hospital Samaritano Rio de Janeiro RJ Brasil Hospital Samaritano, Rio de Janeiro, RJ – Brasil; 11 Rede UnitedHealth Group Rio de Janeiro RJ Brasil Rede UnitedHealth Group (UHG), Rio de Janeiro, RJ – Brasil; 12 Hospital Pasteur Rio de Janeiro RJ Brasil Hospital Pasteur, Rio de Janeiro, RJ – Brasil; 13 Hospital Américas Rio de Janeiro RJ Brasil Hospital Américas, Rio de Janeiro, RJ – Brasil; 14 Hospital de Clínicas Mário Lioni Rio de Janeiro RJ Brasil Hospital de Clínicas Mário Lioni, Rio de Janeiro, RJ – Brasil; 15 Universidade Estadual de Londrina Londrina PR Brasil Universidade Estadual de Londrina, Londrina, PR – Brasil; 16 PRENOTO Clínica Médica e Diagnóstica Ribeirão Preto SP Brasil PRENOTO Clínica Médica e Diagnóstica, Ribeirão Preto, SP – Brasil; 17 Instituto Dante Pazzanese de Cardiologia São Paulo SP Brasil Instituto Dante Pazzanese de Cardiologia, São Paulo, SP – Brasil; 18 Rede Mater Dei de Saúde Belo Horizonte MG Brasil Rede Mater Dei de Saúde, Belo Horizonte, MG – Brasil; 19 Instituto Fanilda Barros Vitória ES Brasil Instituto Fanilda Barros, Vitória, ES – Brasil; 20 Pontifícia Universidade Católica do Paraná Curitiba PR Brasil Pontifícia Universidade Católica do Paraná (PUCPR), Curitiba, PR – Brasil; 21 Faculdade de Medicina do ABC Paulista SP Brasil Faculdade de Medicina do ABC Paulista, SP – Brasil; 22 Faculdade de Medicina Unoeste Guarujá SP Brasil Faculdade de Medicina Unoeste, Guarujá, SP – Brasil; 23 Hospital de Clínicas de Itajubá Itajubá MG Brasil Hospital de Clínicas de Itajubá, Itajubá, MG – Brasil; 24 Clínica Vascularline São Paulo SP Brasil Clínica Vascularline, São Paulo, SP – Brasil; 25 Ecocenter Medicina Diagnóstica Belo Horizonte MG Brasil Ecocenter Medicina Diagnóstica, Belo Horizonte, MG – Brasil; 26 Hospital Ipiranga São Paulo SP Brasil Hospital Ipiranga, São Paulo, SP – Brasil; 27 Hospital São Luiz Itaim Rede D’Or São Luiz São Paulo SP Brasil Hospital São Luiz Itaim, Rede D’Or São Luiz, São Paulo, SP – Brasil; 28 Escola Paulista de Medicina Universidade Federal de São Paulo São Paulo SP Brasil Escola Paulista de Medicina da Universidade Federal de São Paulo (EPM/UNIFESP), São Paulo, SP – Brasil; 29 Hospital Albert Einstein São Paulo SP Brasil Hospital Albert Einstein, São Paulo, SP – Brasil; 30 Instituto do Coração Faculdade de Medicina Universidade de São Paulo São Paulo SP Brasil Instituto do Coração (InCor) da Faculdade de Medicina da Universidade de São Paulo (FMUSP), São Paulo, SP – Brasil; 31 Hospital do Coração São Paulo SP Brasil Hospital do Coração (Hcor), São Paulo, SP – Brasil; 32 Universidade Federal Fluminense Niterói RJ Brasil Universidade Federal Fluminense (UFF), Niterói, RJ – Brasil; 33 Universidade de São Paulo Ribeirão Preto SP Brasil Universidade de São Paulo (USP), Ribeirão Preto, SP – Brasil; 34 Hospital Madre Teresa Belo Horizonte MG Brasil Hospital Madre Teresa, Belo Horizonte, MG – Brasil

**Table:** 

Atualização da Recomendação para Avaliação da Doença das Artérias Carótidas e Vertebrais pela Ultrassonografia Vascular: DIC, CBR, SBACV – 2023	
O relatório abaixo lista as declarações de interesse conforme relatadas à SBC pelos especialistas durante o período de desenvolvimento deste posicionamento, 2022/2023.	
Especialista	Tipo de relacionamento com a indústria
Adriano José de Souza	Nada a ser declarado
Alair Augusto Sarmet Moreira Damas dos Santos	Nada a ser declarado
Ana Cláudia Gomes Pereira Petisco	Nada a ser declarado
Ana Cristina Lopes Albricker	Nada a ser declarado
Ana Luiza Dias Valiente Engelhorn	Nada a ser declarado
Anna Karina Paiva Sarpe	Outros relacionamentos Financiamento de atividades de educação médica continuada, incluindo viagens, hospedagens e inscrições para congressos e cursos, provenientes da indústria farmacêutica, de órteses, próteses, equipamentos e implantes, brasileiras ou estrangeiras: - Sigvaris e Venosan.
Armando Luis Cantisano	Nada a ser declarado
Arthur Curtarelli de Oliveira	Nada a ser declarado
Bruno de Lima Naves	Outros relacionamentos Financiamento de atividades de educação médica continuada, incluindo viagens, hospedagens e inscrições para congressos e cursos, provenientes da indústria farmacêutica, de órteses, próteses, equipamentos e implantes, brasileiras ou estrangeiras: - Bayer: Xarelto; Apsen: Dobeven.
Carlos Alberto Engelhorn	Nada a ser declarado
Carlos Eduardo Rochitte	Declaração financeira A - Pagamento de qualquer espécie e desde que economicamente apreciáveis, feitos a (i) você, (ii) ao seu cônjuge/companheiro ou a qualquer outro membro que resida com você, (iii) a qualquer pessoa jurídica em que qualquer destes seja controlador, sócio, acionista ou participante, de forma direta ou indireta, recebimento por palestras, aulas, atuação como proctor de treinamentos, remunerações, honorários pagos por participações em conselhos consultivos, de investigadores, ou outros comitês, etc. Provenientes da indústria farmacêutica, de órteses, próteses, equipamentos e implantes, brasileiras ou estrangeiras: - Eventual Palestrante Honorário – Pfizer: Amiloidose; GE: Tomografia Cardiovascular; Edwards: TAVI; Manole: Livros de RMC e TCC. B - Financiamento de pesquisas sob sua responsabilidade direta/pessoal (direcionado ao departamento ou instituição) provenientes da indústria farmacêutica, de órteses, próteses, equipamentos e implantes, brasileiras ou estrangeiras: - V-Plaque (Inclisiran- Novartis): Instituição Hcor. Outros relacionamentos Participação societária de qualquer natureza e qualquer valor economicamente apreciável de empresas na área de saúde, de ensino ou em empresas concorrentes ou fornecedoras da SBC: - Blume Medicina Diagnóstica: acionista
Carmen Lucia Lascasas Porto	Outros relacionamentos Financiamento de atividades de educação médica continuada, incluindo viagens, hospedagens e inscrições para congressos e cursos, provenientes da indústria farmacêutica, de órteses, próteses, equipamentos e implantes, brasileiras ou estrangeiras: - Bayer: palestras.
Claudia Maria Vilas Freire	Nada a ser declarado
Domingos de Morais Filho	Nada a ser declarado
Érica Patrício Nardino	Nada a ser declarado
Fanilda Souto Barros	Nada a ser declarado
José Aldo Ribeiro Teodoro	Nada a ser declarado
Luisa Ciucci Biagioni	Nada a ser declarado
Marcelo Rodrigo de Souza Moraes	Nada a ser declarado
Marcio Vinícius Lins de Barros	Nada a ser declarado
Melissa Andreia de Moraes Silva	Nada a ser declarado
Miguel José Francisco Neto	Nada a ser declarado
Mohamed Hassan Saleh	Nada a ser declarado
Monica Luiza de Alcantara	Declaração financeira A - Pagamento de qualquer espécie e desde que economicamente apreciáveis, feitos a (i) você, (ii) ao seu cônjuge/companheiro ou a qualquer outro membro que resida com você, (iii) a qualquer pessoa jurídica em que qualquer destes seja controlador, sócio, acionista ou participante, de forma direta ou indireta, recebimento por palestras, aulas, atuação como proctor de treinamentos, remunerações, honorários pagos por participações em conselhos consultivos, de investigadores, ou outros comitês, etc. Provenientes da indústria farmacêutica, de órteses, próteses, equipamentos e implantes, brasileiras ou estrangeiras: - Boston Scientific: Watchman FLX.
Orlando Carlos Glória Veloso	Nada a ser declarado
Peter Célio Françolin	Outros relacionamentos Participação societária de qualquer natureza e qualquer valor economicamente apreciável de empresas na área de saúde, de ensino ou em empresas concorrentes ou fornecedoras da SBC: - Ensino.
Rogerio Iquizli	Nada a ser declarado
Salomon Israel do Amaral	Outros relacionamentos Participação societária de qualquer natureza e qualquer valor economicamente apreciável de empresas na área de saúde, de ensino ou em empresas concorrentes ou fornecedoras da SBC: - Imagem Cardiovascular.
Simone Nascimento dos Santos	Nada a ser declarado
Valdair Francisco Muglia	Nada a ser declarado

## Sumário

1. Classe de Recomendação e Nível de Evidência 5

2. Resumo das Principais Orientações e

Recomendações 5

3. Introdução e Equipamento 6

3.1. Introdução 6

3.2. Higienização e Prevenção de Infecções 8

4. Espessura Mediointimal e Detecção de

Placas das Artérias Carótidas para Avaliação do

Risco Cardiovascular 8

4.1. Definição Ultrassonográfica da Espessura Mediointimal e da

Placa Carotídea 9

5. Avaliação das Estenoses Carotídeas 9

5.1. Critérios Anatômicos 9

5.2. Papel da Angiotomografia e Angiorressonância 10

5.3. Critérios de Velocidade 11

5.4. Considerações Técnicas para a Avaliação ao Doppler 11

5.5. Estenose da Artéria Carótida Interna 11

**5.5.1. Estenoses Menores que 50%** 12

**5.5.2. Estenoses Maiores que 50%** 12

**5.5.3. Suboclusões e Oclusões** 14

5.6. Estenose da Artéria Carótida Comum e Artéria Carótida Externa 14

5.7. Condições que Afetam as Medidas de Velocidade 15

6. Avaliação Ultrassonográfica após Endarterectomia e

Implante de *Stent* 15

6.1. Introdução 15

6.2. Protocolo do Exame 16

6.3. Avaliação ecográfica após endarterectomia carotídea 16

6.4. Achados do Exame de USV Pós-endarterectomia 16

7. Avaliação Morfológica das Placas Carotídeas 16

7.1. Estudo da morfologia da placa 16

**7.1.1. Morfologia da Placa** 16

**7.1.2. Características das Placas Ateroscleróticas e Risco de DCV** 16

**7.1.3. Medida do Volume da Placa** 16

7.2. Caracterização da Placa Aterosclerótica pela Angiotomografia e

Angiorressonância Magnética 19

**7.2.1. Dissecção de Vasos Cervicais** 19

8. Agente de Realce de Ultrassom na Caracterização da

Placa Aterosclerótica 19

8.1. Características e Propriedades dos Agentes de

Realce de Ultrassom 19

8.2. Aspectos Técnicos que Influenciam a Obtenção de Imagem

com Contraste 19

8.3. Índice Mecânico 20

8.4. Ganho de Imagem 20

8.5. Quantidade de Contraste 20

8.6. Diagnóstico de Oclusão e Sub Oclusão 20

8.7. Avaliação da Neovascularização e Vulnerabilidade das Placas 20

8.8. Dissecção 21

8.9. Inflamação 21

8.10 Avaliação de Stent 21

8.11 Preparação do Contraste 21

8.12. Protocolo Básico de Exame de Ultrassonografia Vascular

com Contraste de Microbolhas 22

9. Avaliação da Doença Ateromatosa em Artérias

Vertebrais 22

9.1. Introdução 22

9.2. Avaliação Ultrassonográfica de Vertebrais 22

9.3. Metodologia do Exame de Rotina 22

9.4. Parâmetros Normais 22

9.5. Quantificação da Estenose 22

**9.5.1. Estenose Proximal (V0-V1)** 22

**9.5.2. Estenose Vertebral nos Demais Segmentos (V2-V4)** 23

**9.5.3. Oclusão de Vertebral** 23

9.6. Síndrome do Roubo pela Artéria Subclávia 23

10. Doppler Transcraniano na Doença Aterosclerótica

Carotídea e Vertebral Extracraniana 23

10.1. Técnicas do Exame 23

10.2. Protocolo Padrão do DTC “Cego” Convencional 25

10.3. Protocolo padrão de Doppler Transcraniano em

Monitorização Contínua 27

10.4. Utilidade clínica do Doppler transcraniano na doença

aterosclerótica cervical 27

**10.4.1. Identificação de Pacientes com HITS** 27

**10.4.2. Repercussões Hemodinâmicas Induzidas** 27

**10.4.3. Avaliação de Estenose Vertebral Intracraniana (V4)** 28

10.5. Recomendações 29

Referências 29

## 1. Classe de Recomendação e Nível de Evidência

As declarações de consenso foram classificadas conforme o mostrado nos Quadros 1 e 2, de acordo com os padrões adotados pela Sociedade Brasileira de Cardiologia (SBC).

**Quadro 1:** – Classe de recomendação de acordo com os padrões adotados pela Sociedade Brasileira de Cardiologia.

Definição	Classe de recomendação
Condições para as quais há evidências conclusivas ou, na sua falta, consenso do grupo.	I
Condições para as quais há evidências conflitantes e/ou divergências de opinião sobre a utilidade do método.	II
Peso ou evidência/opinião a favor do método. Aprovado pela maioria dos autores.	IIa
Segurança e utilidade menos bem estabelecidas, não havendo predomínio de opiniões a favor.	IIb
Condições para as quais há evidências e/ou consenso de que o método não é útil.	III

**Quadro 2:** – Níveis de evidência de acordo com os padrões adotados pela Sociedade Brasileira de Cardiologia.

Definição	Classe de recomendação
Dados obtidos a partir de múltiplos estudos randomizados de bom porte, concordantes e/ou de metanálise robusta de estudos clínicos randomizados.	A
Dados obtidos a partir de metanálise menos robusta, a partir de um único estudo randomizado ou de estudos não randomizados (observacionais).	B
Dados obtidos a partir de opiniões consensuais de especialistas.	C

## 2. Resumo das Principais Orientações e Recomendações

O resumo das principais orientações desse painel de especialistas está descrito no Quadro 3.

**Quadro 3:** – Resumo dos principais pontos sobre a ultrassonografia vascular do sistema carotídeo. ACC: artéria carótida comum; ACE: artéria carótida externa; ACI: artéria carótida interna; USV: ultrassonografia vascular; VDF: velocidade diastólica final; VPS: velocidade de pico sistólico.

Orientações do painel de especialistas	Classe de recomendação	Nível de evidência	Referência
A USV é recomendada como método de primeira escolha para avaliação da doença arterial carotídea sintomática ou assintomática pelas grandes sociedades internacionais.	I	B	1
Devem ser avaliadas as artérias carótidas comuns, internas e externas bilateralmente, em toda a sua extensão, além do tronco braquiocefálico.	I	B	1
A avaliação da estenose carotídea pela USV envolve critérios hemodinâmicos que incluem medidas da velocidade do fluxo e suas relações (razões) a partir do Doppler espectral, associadas à avaliação anatômica da placa e quantificação da estenose local – avaliação multiparamétrica.	I	B	1-3
A VPS é considerada o critério hemodinâmico mais bem estabelecido para a quantificação das estenoses das artérias carótidas internas, apresentando melhor correlação com a angiografia.	I	B	1-4
A VDF e as relações (razões) de velocidades colaboram para o diagnóstico das estenoses e têm grande valor em situações em que a VPS, enquanto valor absoluto, pode não refletir adequadamente o grau de estenose.	I	B	1-4
Em casos de suboclusão da artéria carótida interna, pode haver ou não elevação da velocidade do fluxo e, eventualmente, a não identificação do fluxo. Em casos duvidosos, é recomendada a realização de outro exame complementar.	I	B	1,5-7
A presença de fluxo de velocidade reduzida e padrão de alta resistência em artéria carótida comum pode sugerir a presença de oclusão da artéria carótida interna ipsilateral.	I	B	1,6,8,9
As recomendações para a graduação das estenoses da ACI não devem ser aplicadas para classificar as lesões na ACC ou na ACE.	I	B	1
A avaliação das estenoses na ACC, pode ser realizada pela razão de velocidades sistólicas pré-estenose e no local da estenose, assim como pela quantificação anatômica.	I	B	1,10,11
As estenoses na ACE podem ser quantificadas pela elevação da VPS, assim como pela razão entre a VPS no ponto da estenose e a VPS na artéria carótida comum.	I	B	1,12-14

O Quadro 4 resume a revisão das recomendações do posicionamento de 2015^1^ e as novas recomendações em relação aos tópicos higienização do equipamento, definição de placa carotídea, espessura mediointimal, graduação da estenose e morfologia de placa.

**Quadro 4:** – Recomendações revisadas selecionadas e novas recomendações. Angio-TC: angiotomografia; angio-RM: angiografia por ressonância magnética; ASE: Sociedade Americana de Ecocardiografia; CC: carótida comum; CI: carótida interna; DTC: Doppler transcraniano; EMI: espessura mediointimal; NR: nova recomendação; PC: placa carotídea; R: revisada; US: ultrassom; USV: ultrassonografia vascular; VDF: velocidade diastólica final; VPS: velocidade do pico sistólico.

Nova recomendação ou revisada	Recomendação 2015	Classe de recomendação	Recomendação 2023	Classe de recomendação	Nível de evidência
**Higienização do equipamento**					
NR			Realizar desinfecção/esterilização dos equipamentos conforme a classificação do procedimento.	I	B
**Definição de PC**					
R	Estrutura focal estendendo-se no mínimo 0,5 mm para a luz do vaso, e/ou medindo mais do que 50% do valor da medida da EMI adjacente, e/ou ainda uma medida de EMI maior que 1,5 mm	I	Classificação da ASE 2020(26) – Ênfase na altura e aspecto focal ou difuso da PC em gradação de risco (< 1,5 mm, entre 1,5 e 2,4 mm, ≥ 2,5 mm).	I	B
NR			Análise da carga aterosclerótica e volume da placa aterosclerótica pelo 3D	IIa	B
**Espessura mediointimal**					
R	Na ausência de PC, a descrição da EMI no laudo ficará a critério do ultrassonografista ou de acordo com a solicitação do médico assistente.	I	Não há novas recomendações. A medida da EMI não é recomendada como rotina na população geral.	I	B
**Graduação da estenose das carótidas**					
R	Critério anatômico local deve ser usado para caracterizar estenoses inferiores a 50%.	I	Não há novas recomendações.	I	B
NR			Angio-TC e angio-RM – pacientes sintomáticos, para avaliar o grau de obstrução, quando este não tiver sido obtido pela USV.	I	B
R	Classificação das estenoses em decis na avaliação hemodinâmica multiparamétrica.	I	Não há novas recomendações – Tabela 2.	I	B
R	VPS é o critério de maior acurácia	I	Não há novas recomendações – Tabela 2.	I	B
R	VDF e razão VPS CI/VPS CC são considerados critérios adicionais na avaliação multiparamétrica.	I	Não há novas recomendações – Tabela 2.	I	B
R	Razão VPS CI/VDF CC é critério adicional de menor acurácia e pode ser usado na avaliação multiparamétrica no caso de não haver concordância com os outros parâmetros.	I	Não há novas recomendações – Tabela 2.	I	B
R	Suboclusão – presença de fluxo filiforme ao mapeamento em cores *(string sign* ou *trickle flow*).	I	Angio-TC – Suboclusão: demonstra “colapso parcial”. Medida do lúmen < 1,3 mm, medida do calibre da CI distal < 3,5 mm, relação carótida afetada/carótida contralateral < 0,87, relação CI afetada/CE ipsilateral < 1,27	I	B
R	Oclusão – ausência de perviedade e ausência completa do fluxo sanguíneo, bem como fluxo de alta resistência na CC e fluxo de altíssima resistência no ponto da pré-oclusão.	I	Angio-TC – oclusão: demonstra “colapso total” (sinal do cordão)	I	B
**Morfologia da PC**					
R			Não houve modificação nas classificações.	I	B
NR			Uso do agente de realce do US (com especificações técnicas) para identificação das placas vulneráveis – presença de neovascularização.	I	B
**Doppler transcraniano**					
NR			Investigação de “microembolia silenciosa” deve ser realizada com aparelho de DTC “cego” com capacete para fixação dos transdutores no crânio.	I	B
NR			Avaliação pré-endarterectomia da “reserva vasomotora cerebral”.	I	B
NR			Monitorização perioperatória e no mínimo nos 90 minutos imediatos pós-endarterectomia.	I	B
NR			Inclusão da avaliação dos segmentos intracranianos de vertebrais e de basilar (via janela foraminal) nos exames de rotina de carótidas e vertebrais cervicais de pacientes sintomáticos e sem lesões anatômicas extracranianas que justifiquem a clínica.	I	B

## 3. Introdução e Equipamento

### 3.1. Introdução

O uso do ultrassom (US) na medicina foi iniciado nos anos 1940 e, desde então, vem tendo papel importante no diagnóstico das doenças cardiovasculares (DCV). Devido à sua ampla aplicabilidade, relativo baixo custo e reprodutibilidade, o US tem seu papel estabelecido no auxílio diagnóstico de diversas patologias. Esta diretriz foi elaborada por cardiologistas membros do Departamento de Imagem Cardiovascular (DIC) da Sociedade Brasileira de Cardiologia (SBC), angiologistas e cirurgiões vasculares membros da Sociedade Brasileira de Angiologia e Cirurgia Vascular (SBACV) e radiologistas membros do Colégio Brasileiro de Radiologistas (CBR), especialistas em ultrassonografia vascular (USV), com o objetivo de orientar a melhor utilização dessa técnica, dentro dos conhecimentos recomendados na literatura médica atual, atualizando, com o mesmo enfoque, a diretriz previamente publicada em 2015.^1^

A fundamentação do diagnóstico pela USV de importantes patologias foi embasada nas recomendações do painel de especialistas do DIC de 2015, 2016 e 2019.^1,15,16^ Outros tópicos foram adicionados nessa atualização, como USV transcraniana, utilização de agente de realce ultrassonográfico e alguns pontos do diagnóstico de estenoses carotídea pela angio-TC (angiotomografia) e angio-RM (angiografia por ressonância magnética). Entretanto, o leitor interessado deverá recorrer a publicações mais amplas e específicas sobre essas outras modalidades de imagem.

Nosso objetivo é difundir as melhores práticas da USV entre os profissionais da área, homogeneizar a interpretação dos exames e contribuir para um aproveitamento adequado dessa ferramenta não invasiva, amplamente disponível e de baixo custo.

A descrição sobre equipamentos, aplicativos, transdutores e aspectos relacionada à imagem estão descritas na íntegra da Recomendação de 2015.^1^

### 3.2. Higienização e Prevenção de Infecções

Além dos requisitos tecnológicos e técnicos dos aparelhos e do examinador, é fundamental mencionar a importância da higienização dos aparelhos e medidas de prevenção de infecção entre os profissionais. Qualquer equipamento médico de diagnóstico que entre em contato com o paciente gera risco de infecção. O risco de infecção é baixo, mas existem relatos de contaminação do transdutor de ultrassom, principalmente endocavitários e associados à inserção de acesso central, além da contaminação do gel por múltiplas bactérias.

Na classificação de Spaulding,^17^ que determina a necessidade de esterilização/desinfecção dos equipamentos, os procedimentos com USV são classificados como: 1) críticos, quando o transdutor encosta em tecido estéreis; 2) semicríticos, quando acessa membranas mucosas, tecidos não íntegros (com ou sem contaminação por sangue); e 3) não críticos, quando não há contato com tecidos estéreis, mucosas ou tecidos não íntegros. Para os procedimentos críticos, é necessária a limpeza e esterilização ou higienização completa (HC ou HLD – *high level desinfection*). Nos classificados como semicríticos, a limpeza associada à HC é suficiente. Já para os não críticos, apenas a limpeza superficial (LS ou LLD – *low level desinfection*) é necessária.

A maior parte dos exames diagnósticos de carótida e transcranianos se enquadram como não críticos. A utilização de coberturas (luvas, preservativos ou envelope plástico) não é recomendada, porém a higienização também deve ser mantida. Após o exame, o transdutor deve ser higienizado com pano para a retirada do gel, seguido de uso de água e sabonete, aguardando a secagem antes da desinfecção, que deve abordar o transdutor, cabo e teclado, com produtos como compostos com amônio quaternário, álcool ou fenóis. Caso haja necessidade de HC, recomenda-se manter o transdutor submerso em solução de gluteraldeído, peróxido de hidrogênio ou ácido periacético por 8 a 15 minutos. É necessário manter o cuidado com a prevenção da infecção relacionada ao exame, que, apesar de mínima, pode ocorrer, principalmente em laboratórios, clínicas e hospitais em que são realizados exames diversos. É sempre importante confirmar com o fabricante do aparelho quais soluções higienizadoras podem ser utilizadas, já que elas podem danificar plástico do transdutor e do cabo.

## 4. Espessura Mediointimal e Detecção de Placas das Artérias Carótidas para Avaliação do Risco Cardiovascular

Com a publicação das diretrizes brasileiras de 2007, 2013 e 2019,^1,16,18-20^ dos documentos de consenso de Mannheim 2004-2006-2011^21^ e do consenso da Sociedade Americana de Ecocardiografia,^22^ os especialistas brasileiros na área da USV se mobilizaram para difundir a prática correta da medida da espessura mediointimal (EMI) e da detecção da placa aterosclerótica das artérias carótidas.

Sabe-se que os fatores de risco cardiovasculares tradicionais estão associados ao aumento da EMI.^23-25^ O aumento da EMI parece envolver principalmente a camada média, enquanto a formação da placa carotídea se relaciona ao espessamento da camada íntima e o seu crescimento em direção ao lúmen do vaso.^26^

Estudos clínicos adotaram uma ampla variedade de limites da EMI e, notadamente, o ponto de corte da estratificação de risco baseada em valores numéricos depende das características de base dos indivíduos. Polak et al.^27^ mostraram, em recente publicação, uma *score* de percentil combinado com medições da EMI na artéria carótida comum (ACC) distal e artéria carótida interna (ACI) proximal, que melhoraram a predição de risco de eventos cardiovasculares além do alcançado por fatores de risco tradicionais, mesmo quando adicionada a medida do escore de cálcio no modelo do estudo.

Embora a medida da EMI não seja recomendada “de rotina” na população geral, se pensarmos na predição de risco cardiovascular como uma estimativa a longo prazo, o valor dessa medida talvez seja de relevante importância.^28^ Chamamos atenção que, no contexto do envelhecimento populacional, deve-se ter atenção a uma possível superestimação do risco cardiovascular em idosos com poucos fatores de risco, levando ao uso excessivo de medicações. Uma acurada identificação dos que seriam verdadeiramente de baixo risco poderia resultar em melhor evolução clínica, com prováveis implicações econômicas. Uma recente subanálise do estudo MESA comparou a habilidade de marcadores de risco “negativos” em modificar para baixo a estimativa do risco cardiovascular em 10 anos, entre eles, a medida da EMI < percentil 25.^29^

A diretriz brasileira de dislipidemia de 2017^20^ caracteriza a placa aterosclerótica como uma medida da EMI > 1,5 mm, sendo, assim, importante para o ecografista vascular saber realizar essas medidas. Outro aspecto importante para a correta realização dessa medida é o fato de ser utilizada em diversos protocolos de pesquisa. A técnica e a interpretação da medida da EMI estão descritas no texto base dessa atualização.^1^

A placa carotídea (PC) é uma manifestação da aterosclerose e parece ser um preditor de risco cardiovascular mais forte do que a medida da EMI isoladamente. Uma recente metanálise que incluiu 11 estudos populacionais, com mais de 54.000 pacientes, demostrou que a PC, quando comparada à EMI, teve uma maior acurácia diagnóstica como preditor de infarto agudo do miocárdio (IAM).^30^ Várias publicações estudaram a PC como indicador prognóstico de eventos cardiovasculares, demonstrando seu poder preditivo para a incidência de DCV da presença de placa carotídea com eventos cardiovasculares.^31-39^

A I Diretriz Brasileira de Prevenção Cardiovascular^40^ e a V Diretriz Brasileira de Dislipidemias e Prevenção da Aterosclerose^19^ recomendam a presença de aterosclerose carotídea subclínica, detectada por metodologia de imagem, como critério de identificação de pacientes em alto risco de eventos coronarianos. Além disso, tanto as diretrizes brasileiras quanto o consenso da Sociedade Americana de Ecocardiografia (SAE)^22^ recomendam a utilização da placa carotídea como fator agravante do risco em pacientes de risco intermediário.

### 4.1. Definição Ultrassonográfica da Espessura Mediointimal e da Placa Carotídea

**Figura 1:**
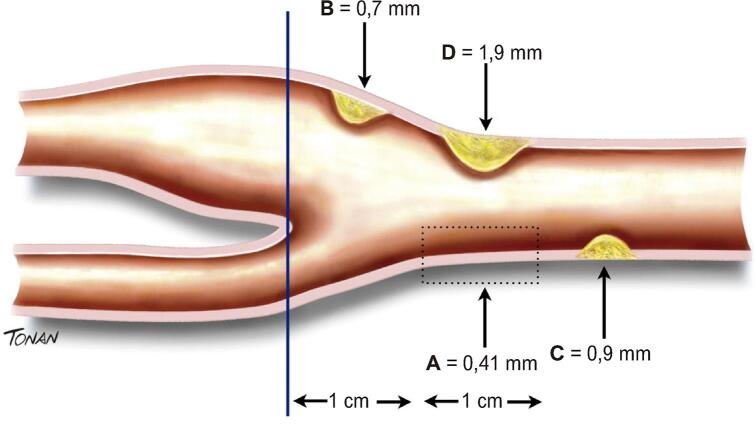
– Ilustração esquemática demonstrando exemplos de medida da espessura mediointimal (EMI) e de placas. Medida da EMI (A). Medidas diferentes de 3 placas carotídeas: B) protusão ≥ 0,5 mm para a luz do vaso; C) medida > 50% do valor da medida da EMI adjacente; D) medida da EMI > 1,5 mm.

A EMI é caracterizada ao modo bidimensional por uma dupla linha com definição das interfaces luz-íntima e média-adventícia. A distância entre as duas interfaces acústicas é considerada a medida da EMI. A PC pode ser definida como uma estrutura focal estendendo-se no mínimo 0,5 mm para a luz do vaso e/ou medindo mais do que 50% do valor da medida da EMI adjacente e/ou, ainda, uma medida de EMI maior que 1,5 mm.^21^ A Figura 1 exemplifica esquematicamente a medida da EMI e das três formas de definição da placa carotídea, como no documento de 2015. Detalhes sobre a técnica e interpretação da medida estão no documento base.

Em recente publicação, Johri et al.^26^ consideraram que a EMI maior ou igual a 1,5 mm pode ser considerada como equivalente de placa ateromatosa, principalmente se a imagem for difusa (Tipo II). Os mesmos autores também classificaram como placa tipo I a presença de protuberância em direção à luz do vaso com medida inferior a 1,5 mm. Esae painel de especialistas entende que a placa classificada como tipo I por Johri et al.^26^ é equivalente às duas primeiras definições de placa do estudo de Mannheim.^7^ Nesse contexto, é importante que o ultrassonografista esteja atento principalmente à classificação da placa tipo I, tendo como parâmetro exames anteriores.

## 5. Avaliação das Estenoses Carotídeas

### 5.1. Critérios Anatômicos

A USV é capaz de avaliar estenoses carotídeas tanto pelo critério de velocidades quanto pela quantificação da estenose feita pelas medidas dos diâmetros residuais, preferencialmente pelo corte transverso.

Os que advogam que a quantificação seja feita por critérios anatômicos se baseiam nas seguintes premissas:^41^ a) critérios de velocidades não permitem discriminar faixas mais estreitas de estenoses devido à sobreposição (“*overlap*”) que ocorre entre as diversas faixas;^42^ b) há grande variação das medidas de velocidades entre os equipamentos, provocando discrepâncias no resultado; c) a correção do ângulo provoca grande variação interobservador; d) houve significativa melhora na qualidade de imagem ultrassonográfica ao modo B nos últimos anos.

Entre os participantes desse painel, houve consenso de que o critério fundamental para a quantificação das estenoses carotídeas é o hemodinâmico. O critério anatômico deve ser usado para caracterizar as estenoses inferiores a 50% (sem repercussão hemodinâmica). Após serem categorizadas pelo critério de velocidade, sugere-se, informar os resultados da faixa de estenose em intervalos de 10%.^43^

Todas as considerações sobre a medida realizada pelo critério anatômico estão detalhadas na diretriz anterior a essa atualização, não tendo sido realizada nenhuma alteração em relação ao documento anterior.^1^

### 5.2. Papel da Angiotomografia e Angiorressonância

Em pacientes com sintomas neurológicos isquêmicos focais correspondentes ao território carotídeo, a angio-TC ou a angio-RM são indicadas para detectar estenose carotídea quando a ultrassonografia não pode ser obtida ou gera resultado não diagnóstico (recomendação Classe I; nível de evidência C).

Tanto a angio-TC quanto a angio-RM, com técnicas de pós-processamento, podem produzir imagens angiográficas semelhantes às da angiografia de subtração digital e permitir medições de estenose de acordo com os critérios NASCET (*North American Symptomatic Carotid Trial*) ou ECST (*European Carotid Surgery Trial*).^41,44,45^ A mensuração (numérica, em porcentagem) do grau de estenose carotídea pode ser feita de formas diferentes a partir desses critérios.^46^

A reprodutibilidade dos diferentes métodos (ultrassonografia, angio-TC e angio-RM) na avaliação de grau de estenose carotídea, em comparação ao padrão-ouro (angiografia digital), tem o benefício adicional de serem técnicas não invasivas e que possibilitam a avaliação do lúmen vascular no plano axial verdadeiro (diferente das projeções ortogonais da angiografia digital) e alguma análise da parede arterial (não avaliável na angiografia por ser uma técnica luminográfica exclusiva).^47^

Atualmente tomografias computadorizadas de alta velocidade e com multidetectores permitem a avaliação direta do diâmetro do lúmen carotídeo e dos tecidos adjacentes com altíssima resolução espacial.^49^ Bartlett et al.^48^ demonstraram uma correlação linear entre a medida em milímetros do lúmen residual, ao nível da estenose da carótida, com o grau de estenose estimado pela angiografia com o método do estudo NASCET.^41^ Os limites de 1,4 a 2,2 mm podem ser usados para avaliar uma estenose moderada (50 a 69%) com uma sensibilidade de 75% e uma especificidade de 93,8%. Um diâmetro da luz residual ≤ 1,3 mm correlaciona-se com uma estenose superior a 70% e pode ser usado como valor de corte, com sensibilidade de 88,2%, especificidade de 92,4% e valor preditivo negativo de 98%, sendo uma ferramenta excelente para diagnosticar ou afastar uma estenose importante.

Cabe destacar a necessidade de identificar os casos de suboclusão carotídea (situação caracterizada por colapso parcial ou total do vaso distalmente ao plano da estenose) quando não deve ser medida numericamente a estenose, sendo qualificada apenas como suboclusão e subclassificada como “com colapso total” quando for observada acentuada redução distal do calibre/sinal do cordão ou “com colapso parcial” quando for observada redução mais discreta do calibre distal do vaso.

A situação de suboclusão “com colapso parcial” nem sempre é clara e evidente, e existem alguns critérios por imagem que podem ajudar sua identificação, evitando erros: calibre do lúmen no plano da estenose < 1,3 mm, medida do calibre da artéria carótida interna distal < 3,5 mm, relação carótida interna doente/carótida interna contralateral < 0,87, relação carótida interna doente/carótida externa do mesmo lado < 1,27 e contrastação menor em relação ao vaso contralateral.^49^

A medida direta da luz residual minimizaria potenciais erros de medida, quando se faz a relação com a luz da carótida interna distal, principalmente nos casos de colapso das paredes nas estenoses acentuadas (Tabela 1).

**Tabela 1:** – Tabela multimodalidade para quantificação das estenoses de carótidas: comparação das percentagens de diminuição do diâmetro distal (angiografia), pelos critérios anatômicos locais (ultrassom – US) e as correspondentes medidas da luz residual pela ultrassonografia e tomografia computadorizada

Arteriografia (NASCET)	%Estenose – US Anatômico Local (ECST)	LÚMEN Residual (mm) US – Imagem do Fluxo (B-Flow)	LÚMEN Residual (mm) Tomografia Comp
Inferior a 20%	< 50%	> 1,5	> 2,2
20-29%	50-55%		
30-39%	58-63%		
40-49%	64-69%		
50-59%	70-75%		1,4-2,2
60-69%	76-81%		
70-79%	82-87%	< 1,5	1,3-1,0
80-89%	88-93%		1,0
90-99%	94-99%		Fluxo filiforme
Oclusão		Ausência de preenchimento	Ausência de preenchimento

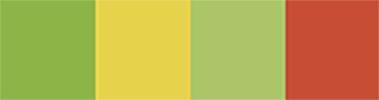
*A escala de cores refere-se a maior experiência da utilização do método pela comunidade científica.*

Suwanwela et al.^50^correlacionaram as medidas de velocidade obtidas pelo Doppler com a medida da luz residual realizada em espécimes cirúrgicas retiradas “em bloco”, sugerindo que uma estenose importante, definida como diâmetro do lúmen residual ≤ 1,5 mm, associada a alterações hemodinâmicas significativas estipuladas pelos critérios de velocidade, tem 100% de especificidade e até 96% de sensibilidade. Mais recentemente, Yurdakul et al.^51^ demonstraram, utilizando a técnica *B-flow*, com melhor resolução temporal e espacial e menor ocorrência do fenômeno de “extravasamento” que o Doppler colorido e o *Power Doppler*, que uma luz residual menor que 1,5 mm apresentava *performance* semelhante à angiografia com subtração digital pelo método NASCET para estimar estenose do ramo interno entre 70 e 99%, com sensibilidade de 93%, especificidade de 94% e acurácia de 94%.

**Figura 2:**
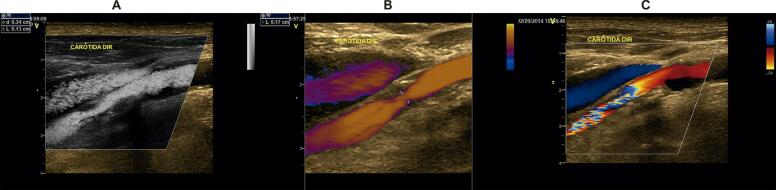
– Medida da luz residual (A) com B-*flow*, (B) B-*flow *angio e (C) Doppler colorido. Nas técnicas relacionadas com o B-*flow*, há menor sobreposição da cor sobre a placa, sendo o método preferível para a medida da luz residual.

A Figura 2 traz a medida da luz residual utilizando-se o *B-flow*, *B-flow angio* e Doppler colorido. A comparação das percentagens de diminuição do diâmetro distal (arteriografia) pelos critérios anatômicos locais (US) e as correspondentes medidas da luz residual pela ultrassonografia e tomografia computadorizada estão demostradas na Figura 2.

### 5.3. Critérios de Velocidade

Várias instituições publicaram seus critérios de avaliação das estenoses por análises das velocidades do fluxo, com algumas diferenças em sua interpretação.^52-58^

Arous et al.^55^ avaliaram 10 instituições americanas e mostraram que, entre elas, havia a utilização de diferentes critérios Doppler ultrassonográficos para a graduação das estenoses carotídeas, gerando diferenças significativas no número de intervenções, subsequentemente impactando em custos no sistema de saúde. Recentemente, Columbo et al.^59^ levantaram dados de 338 centros diagnósticos americanos, em duas populações, 4.791 pacientes ≥ 65 anos do *Cardiovascular Health Study* e 28.483 pacientes assintomáticos, submetidos a revascularização carotídea, pertencentes ao *Vascular Quality Initiative Registry* (www.vqi.org) e que demonstraram uma grande variação de pontos de corte da velocidade de pico sistólica (VPS) entre as instituições, tanto para estenoses maiores que 50%, como para estenoses maiores que 70%, implicando em disparidades no diagnóstico das estenoses e nas decisões de intervenção.

Em editorial referente ao mesmo estudo, Kim e Zierler^57^ reforçaram a necessidade de se normatizar os parâmetros para o diagnóstico das estenoses carotídeas.

Em 2015, o Departamento de Imagem Cardiovascular da Sociedade Brasileira de Cardiologia (DIC-SBC) publicou recomendações para a quantificação das estenoses das artérias carótidas, englobando critérios de avaliação de fluxo ao Doppler, associados à avaliação anatômica da placa. Também teve como objetivo subdividir os graus de estenose em decis, de forma que o resultado ultrassonográfico fornecesse informações mais objetivas, auxiliando na decisão terapêutica.^1^ Dessa forma, como outros autores também sugeriram, houve a aprovação de uma abordagem multiparamétrica para a quantificação das estenoses da ACI.^43,58^

### 5.4. Considerações Técnicas para a Avaliação ao Doppler

A avaliação da velocidade do sangue pelo método Doppler deve ser realizada em conjunto com a avaliação da placa ao bidimensional. Deve-se aferir, ao Doppler pulsado, o traçado espectral nas carótidas comuns, internas e externas bilateralmente e em qualquer ponto em que haja suspeita de estenose sugerida pelas imagens em Modo B e/ou Doppler colorido.^15^ A visualização da placa, seja hipo ou hiperecogênica, assim como calcificada, com ou sem sombra acústica, é imprescindível para o diagnóstico da estenose, uma vez que condições hemodinâmicas diversas podem cursar com velocidades elevadas ou reduzidas independentemente da presença de estenoses.

Para considerações técnicas para a avaliação pelo Doppler, como ângulo de insonação correto e local da medida das velocidades, indicamos a consulta da diretriz anterior a essa atualização.^1^

### 5.5. Estenose da Artéria Carótida Interna

**Figura 3:**
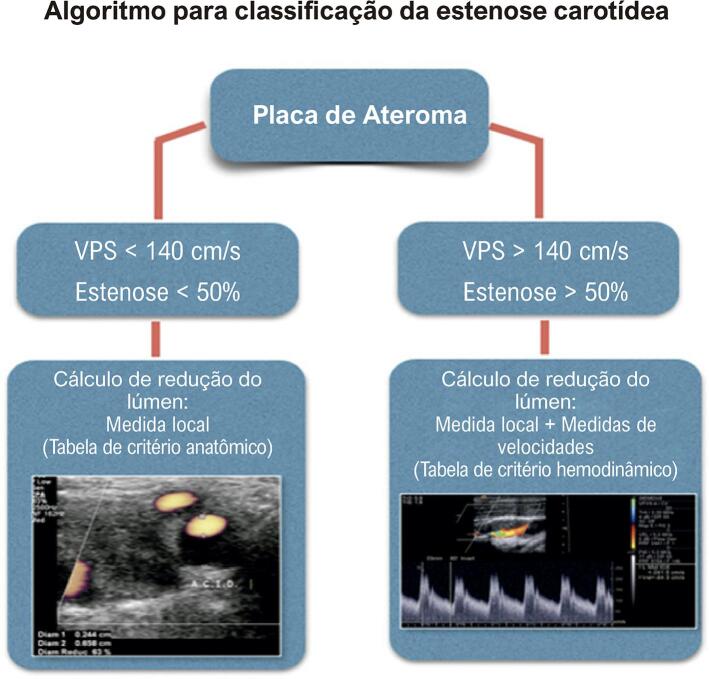
– Recomendação do DIC-SBC para a sequência da avaliação das estenoses carotídeas. VPS: velocidade do pico sistólico.

O presente documento revisa os critérios publicados pelo DIC-SBC em 2015, com atualização dos seus dados. A Figura 3 demonstra a recomendação do DIC-SBC para a sequência da avaliação das estenoses carotídeas.

#### 5.5.1. Estenoses Menores que 50%

Este documento sugere que as subdivisões para a categoria das estenoses menores que 50% continuem sendo realizadas pela análise ao Modo B, utilizando preferencialmente o corte ultrassonográfico transverso que forneça a melhor imagem para o cálculo de redução do lúmen.^43,59^

#### 5.5.2. Estenoses Maiores que 50%

**Figura 4:**
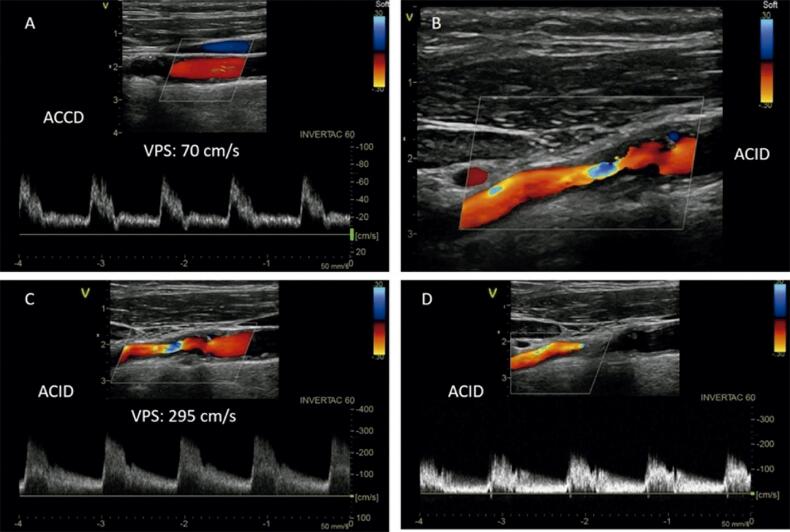
– Estenose entre 70 a 79% da artéria carótida interna. A) Fluxo da artéria carótida comum; B) Estenose da artéria carótida interna ao Doppler colorido; C) Fluxo da artéria carótida interna no ponto da estenose; D) Fluxo turbulento pós-estenótico na artéria carótida interna.

Entre os critérios para a avaliação das estenoses, destaca-se a VPS, que, na presença da placa, é considerada um parâmetro importante e objetivo. No entanto, a análise conjunta com outros parâmetros, como a VDF e as razões de velocidades, confere confiabilidade e facilita o diagnóstico (Figuras 4 e 5). Além disso, com a utilização de diversos parâmetros, é possível estreitar a faixa diagnóstica.

**Figura 5:**
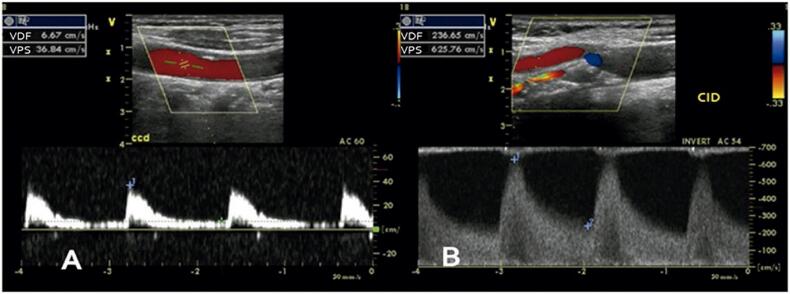
– Estenose > 90% da artéria carótida interna. A) Fluxo da artéria carótida comum; B) Fluxo da artéria carótida interna.

Este documento corrobora a subdivisão da classificação das estenoses da ACI em decis, conforme a Tabela 2, conforme recomendação do DIC 2015.^1^

**Tabela 2:** – Quantificação das estenoses carotídeas

%Estenose (NASCET)	VPS cm/s	VDF cm/s	VPS CI / VPS CC	VPS CI / VDF CC	VDF CI / VDF CC
< 50%	< 140	< 40	< 2,0	< 8	< 2,6
50 – 59%	140 – 230	40 – 69	2,0 – 3,1	8 – 10	2,6 – 5,5
60 – 69%		70 – 100	3,2 – 4,0	11 – 13	
70 – 79%	> 230	> 100	> 4,0	14 – 21	
80 – 89%		> 140		22 – 29	> 5,5
> 90%	> 400		> 5,0	> 30	
Suboclusão	Variável – fluxo filiforme	Variável – fluxo filiforme	Variável – fluxo filiforme	Variável – fluxo filiforme	Variável – fluxo filiforme
Oclusão	Ausência de fluxo	Ausência de fluxo	Não se aplica	Não se aplica	Não se aplica

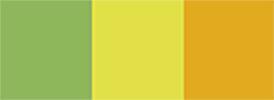
*As cores representam, da esquerda para a direita, os critérios mais relevantes. CC: carótida comum; CI: carótida interna; VDF: velocidade diastólica final; VPS: velocidade de pico sistólico.*

A correlação entre parâmetros de velocidade pela USV com a angiografia já foi demonstrada por diversos autores, conforme apresentado na Tabela 3.^60-62^

**Tabela 3:** – Correlação dos parâmetros de velocidade pela USV com a angiografia (valores de “r”)

	VPS	VDF	VPS CI/VPS CC	VDF CI/VDF CC
AbuRahma et al. (2011)	0,81	0,70	0,57	0,54
Petisco et al. (2015)	0,81	0,78	0,81	-----
Braum et al. (2008)	0,825	0,762	0,766	0,643

A USV tem boa acurácia na identificação das estenoses maiores que 70%, porém o mesmo não acontece para estenoses menores que 50% e, em especial, as entre 50 e 69%.^42,62,63^ Recentemente, Barlinn et al.,^64^ utilizando os critérios da German Society of Ultrasound in Medicine (DEGUM) também mostraram uma sensibilidade menor para a avaliação das estenoses entre 50 e 69% do que nas entre 70 e 99% (sensibilidade de 35% e 81% e especificidade de 89% e 69%, respectivamente).

O consenso de 2003 e o Joint do Reino Unido preconizam o VPS > 230 cm/s para a identificação das estenoses > 70%, tendo sido validado por outros autores em suas instituições.^60,61,62^ AbuRahma et al.^65^encontraram boa acurácia na validação do consenso de 2003, mas sugerem que, para estenoses ACI ≥ 70%, a VPS > 230 cm/s deveria ser usada em pacientes sintomáticos, enquanto, para os assintomáticos, a abordagem multiparamétrica (VPS > 230 cm/s; VDF > 100 cm/s; razão VPS CI/VPS CC > 4) deveria ser considerada, ou então uma VPS > 280 cm/s.

No diagnóstico das estenoses entre 50 e 69%, o consenso de 2003 e o Joint do Reino Unido preconizam a VPS entre 125 e 230 cm/s, no entanto, alguns autores encontraram melhor desempenho com VPS maiores para as estenoses > 50%. AbuRahma et al.^65^mostraram uma melhor especificidade com a VPS ≥ 137 cm/s do que com 125 cm/s (91% x 85%), optando pela VPS de 140 cm/s já utilizada em sua instituição.^66,67^ Valor semelhante foi encontrado em nosso meio por Petisco et al.,^59^ em que a VPS ≥ 141 cm/s apresentou uma melhor especificidade do que a VPS ≥ 125 cm/s (90% x 83%), com acurácia semelhante. Outros valores de VPS estão presentes na literatura, como descritos pelo DEGUM e pelo EQUALIS (External Quality Assurance in Laboratory Medicine in Sweden), em que, respectivamente, VPS > 200 cm/s e 230 cm/s identificariam estenoses ≥ 50 % e VPS maiores que 300 cm/s e 320 cm/s corresponderiam a ≥ 70%.^4,67,68^ Recentemente, Gornick et al.^4^ avaliaram retrospectivamente exames ultrassonográficos de 167 pacientes (299 carótidas), comparando os critérios do consenso de 2003 com a angiografia, e observaram que a VPS ≥ 180 cm/s obteve uma melhor sensibilidade, especificidade e acurácia (93,3%, 81,6% e 85,2%, respectivamente) para identificar as estenoses ≥ 50%, assim como a associação de critérios: a VPS ≥ 125 cm/s, com a razão VPS ACI/VPS ACC ≥ 2 (94,3%, 84,3% e 87,4% respectivamente). Reforçando a necessidade de uma padronização internacional dos critérios ultrassonográficos, propostas recentes contam com uma abordagem multiparamétrica para uma classificação mais precisa das estenoses.^69^

Além da VPS, a VDF pode ser útil no diagnóstico das estenoses > 70% e 80%. O consenso de 2003 sugere a VDF > 100 cm/s como parâmetro adicional para a identificação das obstruções > 70%, assim como outros autores obtiveram boa especificidade utilizando esse parâmetro.^70,71^ Para o diagnóstico das estenoses maiores que 80%, a VDF > 140 cm/s é utilizada há anos pela Universidade de Washington e mostrou-se com especificidade maior que 90% também em outros estudos.^62,72^ Arous et al.^73^ demonstraram que a VPS ≥ 450 cm/s ou a VDF ≥ 120 cm/s identificaram as estenoses ≥ 80% com uma *area under curve* (AUC) de 0,66, não havendo diferença significativa na AUC entre as VDF ≥ 120 cm/s e ≥ 140 cm/s (0,657 x 0,653, respectivamente).

Além das velocidades absolutas, as razões de velocidades são particularmente úteis, seja como adjuvantes na quantificação das estenoses, seja em casos especiais, em que as velocidades podem estar alteradas por outras condições que podem subestimar ou superestimar o grau de estenose. São elas: VPS ACI/VPS ACC, VPS ACI/VDF ACC e VDF ACI/VDF ACC. A mais utilizada é a razão VPS ACI/VPS ACC, avaliada e endossada por diversos estudos.^2,3,43,72-77^ A razão VPS ACI/VDF ACC (índice de *St Mary’s)*, subdivide as estenoses > 50% em decis,^78^ porém não foi muito estudada, podendo haver sobreposição dos valores para graus diferentes de estenose. A razão VDF ACI/VDF ACC pode identificar estenoses > 80% da ACI quando maior que 5,5, segundo alguns autores,^78-80^ mas com correlação inferior com a angiografia.^60,62^

O fluxo pós-estenótico pode auxiliar na identificação das estenoses muito severas e em placas calcificadas, com sombra acústica, quando há turbulência do fluxo após a placa, redução significativa da velocidade (VPS < 30 cm/s) e alargamento do tempo de aceleração.^43,67^ Ressalta-se também a importância de comparar o fluxo pós-estenótico com o fluxo no vaso contralateral.^81^

#### 5.5.3. Suboclusões e Oclusões

O diagnóstico ultrassonográfico da suboclusão baseia-se na demonstração do estreitamento da luz do vaso ao *color* e/ou *power* Doppler, com fluxo filiforme (*string sign* ou *trickle flow)*, porém, pode estar associado a velocidades altas, baixas ou indetectáveis, o que eventualmente dificulta o diagnóstico. Nas suboclusões com presença de velocidade sistólica elevada no ponto da estenose, nota-se redução significativa da velocidade distal à mesma.^5^

Segundo o consenso de 2003 para a diferenciação entre suboclusão e oclusão, recomenda-se não utilizar parâmetros de velocidade ao Doppler, mas, sim, a opinião do observador acerca das imagens. Como a suboclusão pode ser confundida com a oclusão, o Joint do Reino Unido e a AHA (American Heart Association) recomendam a complementação com outro método para definir: angio-TC, angio-RM ou angiografia convencional.^7,61^

**Figura 6:**
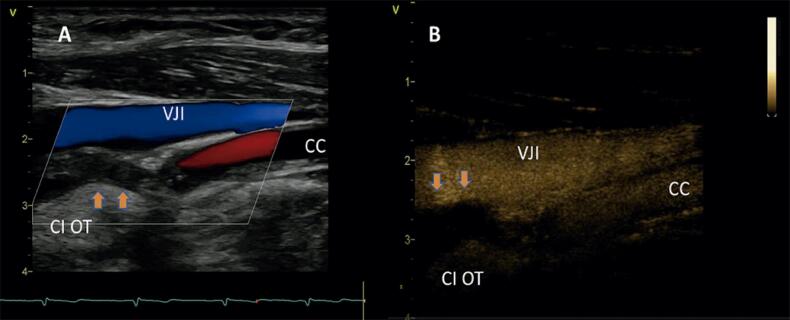
– Oclusão da carótida interna. A) Ausência de fluxo na artéria carótida interna ao Doppler colorido; B) Contraste ultrassonográfico não visualizado na luz da carótida interna ocluída.

As oclusões carotídeas podem ser diagnosticadas pela ultrassonografia como a ausência de luz patente na escala de cinza e ausência de detecção de fluxo ao Doppler colorido*, power* Doppler, Doppler espectral e à avaliação com injeção de contraste com microbolhas, assim como a presença de fluxo de alta resistência na ACC, e do fluxo em *staccato* – fluxo com velocidade muito reduzida e de altíssima resistência no ponto da oclusão ou pré-oclusão^3,81^ (Figura 6).

Na presença de oclusão da ACI, surgem mecanismos compensatórios, como o desenvolvimento de circulação colateral, com o intuito de prevenir a isquemia cerebral, porém a mais importante via de colateralização é pelo polígono de Willis.

Outra via colateral pode ser feita com fluxo anterógrado por ramos distais da ACE ipsilateral que se conectam com o ramo oftálmico da ACI, podendo, então, ser detectado fluxo retrógrado na artéria oftálmica.^8^ Porém, sabe-se que essa condição não está presente em todos os casos de oclusão da ACI devido aos diferentes padrões da circulação retrobulbar.^9^ Assim como se sabe que estenoses hemodinamicamente significativas (maiores que 70% e suboclusões) da ACI podem cursar com fluxo retrógrado da artéria oftálmica.^43^

Nos casos em que há oclusão da artéria carótida comum, a carótida interna pode encontrar-se pérvia, com fluxo anterógrado proveniente da artéria carótida externa e seus ramos.

## 5.6. Estenose da Artéria Carótida Comum e Artéria Carótida Externa

A incidência isolada de estenose da ACC é baixa, e pouco se sabe sobre a evolução dessa lesão. Suspeita-se que pacientes com estenose isolada da ACC apresentem mais sintomas hemisféricos, afasia e amaurose fugaz.^62^

Não há evidências de que as recomendações para a graduação da estenose da ACI devam ser aplicadas para classificar as lesões na ACC ou na ACE.

**Figura 7:**
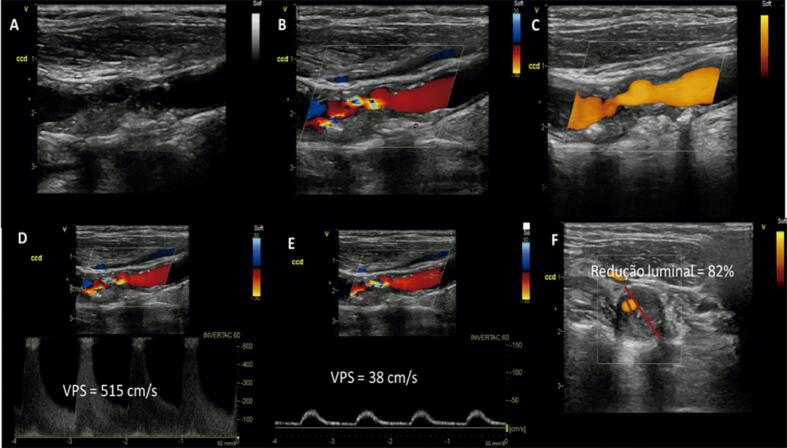
– Estenose da artéria carótida comum. A) Modo B; B) Doppler colorido; C)* Power *Doppler; D) Elevação do VPS no ponto da estenose da artéria carótida comum; E) VPS pré-estenose; F) Corte transversal com importante redução do lúmen residual. VPS: velocidade do pico sistólico.

Este grupo de trabalho recomenda a quantificação da estenose da ACC utilizando não só as medidas de velocidades, mas também a razão de velocidades pré- e no ponto da estenose maior que duas vezes para aquelas maiores que 50% e, principalmente, a análise da redução do lúmen pelo Doppler colorido ou *power* Doppler do fluxo e pelo modo B (Figura 7). Devemos lembrar que pode haver limitações para avaliação das estenoses ostiais da ACC, principalmente à esquerda.

A Tabela 4 sumariza os principais critérios descritos na literatura acerca da estenose da ACE.

**Tabela 4:** – Principais critérios descritos na literatura para a quantificação das estenoses da artéria carótida externa

	Estenoses da artéria carótida externa	
**Autor**	**VPS ACE**	**Razão VPS ACE/ VPS ACC**
Acer et al. (1996)	< 50% ⇢ < 150 cm/s > 60% ⇢ > 250 cm/s	
Paivansalo et al. (1996)		≥ 50% ⇢ ≥ 2
Kronick et al. (2019)	> 50% ⇢ ≥ 200 cm/s com *aliasing*	
Shmelev et al. (2019)	≥ 50%, sem estenose ACI ≥ 50% ⇢ > 148 cm/s ≥ 50%, com estenose ACI ≥ 50% ⇢ > 179 cm/s	> 50%, sem estenose ACI ⇢ ≥ 1,45 > 50%, com estenose ACI ⇢ ≥ 1,89

*ACC: artéria carótida comum; ACE: artéria carótida externa; ACI: artéria carótida interna; VPS: velocidade do pico sistólico.*

## 5.7. Condições que Afetam as Medidas de Velocidade

Algumas condições, seja por estenose arterial, seja por motivos não vasculares locais, afetam as medidas da análise espectral. Elas podem estar localizadas na bifurcação carotídea, distal ou proximal à mesma ou ainda na carótida contralateral. Entre as condições proximais à bifurcação, ressaltamos as valvopatias aórticas (estenose ou insuficiência), estenoses de origem aterosclerótica ou arterites com envolvimento do arco aórtico, ramos e carótida comum.^82-84^ Além das valvopatias, outras circunstâncias, como a disfunção sistólica do ventrículo esquerdo de grau importante, as arritmias cardíacas, taquicardias e bradicardias podem alterar a onda fluxo no sistema arterial, incluindo as artérias carótidas, sem a presença de doença estenótica desses vasos.

Devemos ter em mente que as alterações cardíacas geram efeitos sistêmicos, ou seja, as modificações encontradas nas ondas de fluxo das artérias carótidas estarão presentes bilateralmente, assim como acometem os demais leitos arteriais.^85^

As condições que afetam as medidas de velocidades estão pormenorizadas na diretriz do DIC 2015 precedente a esta atualização.^1^

## 6. Avaliação Ultrassonográfica após Endarterectomia e Implante de *Stent*

### 6.1. Introdução

Intervenções no território carotídeo por via cirúrgica convencional ou por via endovascular são frequentemente realizadas, especialmente para o tratamento de lesões ateroscleróticas. O acompanhamento após esses procedimentos é essencial para identificar precocemente qualquer alteração que possa interferir na perviedade após o tratamento e garantir melhores resultados pós-operatórios.^86^ Sabe-se que o exame de USV apresenta baixo custo e boa acurácia em comparação com a angiografia, porém não há consenso quanto à periodicidade do acompanhamento.^87^

### 6.2. Protocolo do Exame

O exame pós-intervenção é semelhante ao de diagnóstico. É essencial a avaliação e descrição de todos os achados inerentes aos procedimentos.

### 6.3. Avaliação ecográfica após endarterectomia carotídea

O tratamento cirúrgico da estenose carótida é feito por meio de incisão na parede anterior, remoção da placa de aterosclerose e síntese da artéria com ou sem colocação de *patch*.

Uma das principais preocupações após a endarterectomia (EAC) de carótida é a taxa de reestenose e o risco de acidente vascular cerebral (AVC) subsequente^88^ que, felizmente, são infrequentes.^89,90^

As reestenoses diagnosticadas entre 6 e 12 meses após a EAC são, geralmente, decorrentes de hiperplasia neointimal. Já as lesões que se desenvolvem após 24 e 36 meses tendem a representar recorrência do processo aterosclerótico.^91^

AbuRahma et al.^92^ não encontraram valor significativo para a repetição de USV de rotina após EAC com *patch.* Já para Bandyk et al.,^93^ e Zierler et al.,^86^ o benefício da vigilância supera seu risco, e recomendam a vigilância com USV com grau de recomendação 1B.

### 6.4. Achados do Exame de USV Pós-endarterectomia

As suturas de fechamento de arteriotomia podem ser vistas como ecos brilhantes e uniformemente espaçados ao longo da parede da ACC e ACI na imagem em modo B (Figura 7A). Se um *patch* tiver sido usado, ele poderá criar uma dilatação no local da EAC de várias dimensões (Figuras 7B e 7C). Enquanto um *patch* de veia pode ter aparência indistinguível da parede da artéria nativa, a dilatação e as suturas podem ajudar a identificar sua presença. O *patch* de Dacron aparecerá como uma superfície espessa e brilhantemente ecogênica, e o de politetrafluoretileno (PTFE) normalmente aparecerá como uma linha dupla brilhante que representa a espessura do material e os efeitos da penetração do ultrassom.^86^

Devem-se aferir as medidas dos diâmetros no vaso nativo, nos locais das anastomoses e na região do alargamento, caso haja, para que se possa acompanhar e comparar posteriormente.

As principais características ultrassonográficas e complicações após intervenções carotídeas foram descritas e ilustradas nas recomendações do DIC de 2015. Nesta atualização, houve apenas uma mudança nos critérios de reestenose, e o Quadro 5 mostra o resumo, com caráter de reafirmar as definições e incluir a atualização.

**Quadro 5:** – Características ultrassonográficas e complicações principais após procedimentos. ACC: artéria carótida comum; ACE: artéria carótida externa; ACI: artéria carótida interna; USV: ultrassonografia vascular; VDF: velocidade diastólica final; VPS: velocidade do pico sistólico.

Exame pós-endarterectomia	Descrição ao USV
**Desproporção de calibres:** Tempo de ocorrência imediato	Identificação de grande diferença de calibres entre o bulbo carotídeo pós-endarterectomia e o segmento distal da carótida interna (comum com a colocação de patch carotídeo)
**“Degrau”:** Tempo de ocorrência imediato	Identificação de um “degrau” na parede arterial no ponto de abordagem cirúrgica da artéria
**Complicação pós-endarterectomia**	
**Dilatação aneurismática:** Tempo de ocorrência geralmente tardio	Dilatação acentuada no local onde foi realizada a endarterectomia; pode ter trombo associado
**Trombose oclusiva/não oclusiva:** Tempo de ocorrência recente	Presença de imagem hipoecogênica aderida ao local do procedimento, com ou sem componente móvel
**Reestenose:** Tempo de ocorrência: 3 a 24 meses – mecanismo: hiperplasia neointimal 24 meses – mecanismo: aterosclerose	**Atualização:** Redução da luz ao modo B nos cortes transversal e longitudinal com turbilhonamento do fluxo local Estenoses > 70%: VPS > 300 cm/s; VDF => 125 cm/s e razão de VPS ACI/ACC > 5
**Complicação pós-stent**	
**Mal posicionamento** Tempo de ocorrência imediato	Identificação do stent não posicionado no local de maior estenose com manutenção da turbulência no ponto de maior estenose
**Expansão inadequada** Tempo de ocorrência 0 a 24 meses	Medidas dos diâmetros nas extremidades do stent e/ou no corpo do stent com estenose residual > 30%
**Reestenose:** Tempo de ocorrência: 3 a 24 meses – mecanismo: hiperplasia neointimal 24 meses – mecanismo: aterosclerose	**Acima de 50%:** VPS ≥ 220 cm/s e razão VPS ACI/ACC ≥ 2,7 **Acima de 80%:** VPS ≥ 340 cm/s e razão VPS ACI/ACC ≥ 4,15 **Tipos:** I: hiperplasia focal na extremidade do stent (< 10 mm) II: hiperplasia focal intrastent (< 10 mm) III: hiperplasia difusa (> 10 mm) IV: hiperplasia difusa proliferativa (> 10 mm) que estende para fora das extremidades do stent V: Oclusão do stent
**Fratura/torção stent**	USV não é o método de escolha Suspeitar na presença de calcificação significativa e/ou reestenose com Rx alterado
**Efeito do Implante stent na ACE**	Pode levar estenose na origem da ACE com turbilhonamento do fluxo (fluxo passa pela malha do stent)

Apesar de a maioria dos estudos considerar a estenose > 70% após EAC como critério de gravidade na reestenose, o seu ponto de corte varia na literatura. Assim, novos estudos são necessários para a padronização dos critérios de velocidades ultrassonográficos nas reestenoses após EAC. Entretanto, devem ser levadas em consideração as diferenças de velocidade encontradas nas endarterectomias com ou sem *patch* e a possibilidade de desproporção de calibre no final da endarterectomia na carótida interna.

Recomendamos a adoção dos critérios recentes de Bandyk et al.^93^ para a graduação das estenoses > 70% após EAC, sendo VPS > 300 cm/s, VDF > 125 cm/s, razão das VPS ACI/ACC > 5. Para a vigilância pela USV, seguindo os mesmos autores, recomendamos o intervalo de 1, 3 e 12 meses após o procedimento.

## 7. Avaliação Morfológica das Placas Carotídeas

O estudo da morfologia da placa aterosclerótica vem ganhando espaço dentro da avaliação da aterosclerose carotídea. Convencionalmente, a graduação da estenose carotídea sempre teve o papel de maior destaque nos exames de imagem de carótidas e vertebrais, já que é a variável mais utilizada na tomada de decisão cirúrgica da endarterectomia ou do implante de *stent* carotídeo. Entretanto, há mais de 2 décadas estudam-se aspectos morfológicos e histopatológicos ligados à instabilidade da placa aterosclerótica, isto é, placas com o mesmo grau de estenose não necessariamente apresentam o mesmo potencial isquêmico para eventos tromboembólicos. A habilidade de identificar qual placa seria mais instável ou vulnerável pode ter um papel fundamental na decisão terapêutica.

A identificação de uma PC com núcleo rico em lipídios, ulceração ou sinais sugestivos de hemorragia intraplaca (HIP) em pacientes com acidentes vasculares encefálicos (AVEs) repetidos e estenoses não significativas pode direcionar para a intervenção carotídea ou intensificação do tratamento farmacológico de acordo com as melhores práticas médicas.^94^ A definição de placa aterosclerótica se encontra na parte 2 desse documento (Figura 1) e se manteve como a adotada no documento desse mesmo grupo de 2015.^1^

### 7.1. Estudo da morfologia da placa

A caracterização da morfologia da placa tem um papel importante na ocorrência de eventos cerebrovasculares e pode também ser um importante preditor de eventos. A pesquisa das características relacionadas ao maior risco de eventos demonstra um esforço para identificar os parâmetros relacionados à placa que, juntamente com o grau de estenose, podem predizer com maior precisão a presença de placa vulnerável e o risco associado de eventos isquêmicos. O US, entretanto, tem limitações inerentes ao método nessa caracterização. Outros métodos ainda não foram incorporados na rotina para essa avaliação, já que ainda não há nada totalmente estabelecido provando uma melhora adicional na estratificação de risco.^95,96^

#### 7.1.1. Morfologia da Placa

A descrição da morfologia da placa deve ser realizada nos laudos do exame de USV carotídea como já descrito na recomendação do DIC de 2015, de acordo com o Quadro 5.^1^ A caracterização da placa aterosclerótica pode ser preditiva de progressão do grau de estenose e eventos clínicos. Placas hipoecogênicas, heterogêneas e irregulares são marcadoras de risco de eventos como AVC e ataque isquêmico transitório (AIT).

Neste documento, atualizamos em relação ao valor de algumas características das placas ateroscleróticas e risco de DCV, avaliação do volume de placa e dados da angio-TC e angio-RM.

#### 7.1.2. Características das Placas Ateroscleróticas e Risco de DCV

Herr et al. utilizaram um método semelhante ao GSM em pacientes avaliados para doença cardiovascular. Os autores concluíram que o aumento da ecogenicidade da placa carotídea (tecido fibroso e/ou semelhante ao cálcio) se correlacionou com o aumento de doença arterial coronária, e uma combinação de altura da placa, percentual de cálcio e/ou percentual de gordura aumenta o risco de eventos cardiovasculares. Esse estudo aponta para o potencial de incorporação da análise da composição da placa pelo ultrassom carotídeo na estratificação de risco (Quadro 6).^97^

**Quadro 6:** – Sumário das caracterizações da placa aterosclerótica e risco cardiovascular. Angio-RM: angiografia por ressonância magnética; angio-TC: angiotomografia; AVE: acidente vascular encefálico; RM: ressonância magnética; US: ultrassom. *Essa análise não tem boa acurácia com exames perioperatórios e está reduzida na presença de cálcio e placas estenóticas.

Característica da placa	Definição	Risco clínico/referência
**Ecogenicidade**	*Tipo I:* uniformemente ecolucente *Tipo II:* predominantemente ecolucente *Tipo III:* predominantemente ecogênica *Tipo IV:* uniformemente ecogênica *Tipo V:* calcificada.	Placas dos Tipos 2 e 3 são associadas a maior risco de AVEs e as dos Tipos IV e V as mais estáveis.^1^
**Localização**	Descrever em qual segmento do sistema carotídeo a placa se encontra: carótida comum, bifurcação, ramos externo e interno proximal e médio.	
Superfície*	*1. Regular:* < 0,4 mm de profundidade *2. Irregular:* irregularidade 0,4 a 2,0 mm de profundidade *Ulceração:* De Bray – concavidade e extensão > 2,0 mm; base bem definida e fluxo reverso na concavidade ao color Muraki – concavidade clara e ecogenicidade da base menos intensa que da parede adjacente *3. Com ou sem componente móvel:* informar o tamanho se presente.	A presença de irregularidade e ulceração tem elevado risco de eventos.^1^
**Hemorragia intraplaca**	Área anecoica próxima à superfície da placa com capa fibrótica íntegra.	Marcador de vulnerabilidade pela associação significativa com eventos cerebrovasculares; acontece em placas com e sem comprometimento hemodinâmico e parece ser causada por ruptura de neovascularização intraplaca ou da própria placa aterosclerótica.^1,103-105^
**Volume da placa**	Equivale ao conteúdo aterosclerótico medido em um segmento arterial definido pelo 3D, possibilitando o acompanhamento da progressão da lesão e do tratamento.	O volume total da placa, medido de 1,5 cm da artéria carótida comum distal até 1 cm distal à bifurcação, é fator preditor de eventos futuros de doença cardiovascular.^26,98,99^
**Análise da angio-TC e angio-RM**	Vantagem de ambos: possuem resolução espacial submilimétrica, mas não são utilizados para avaliação de risco cardiovascular – *Vessel wall imaging*: recurso técnico recente para diagnóstico de hematomas intramurais e dissecção pela RM.	Muito úteis para o diagnóstico da dissecção de vasos cervicais agudos e subagudos e hematoma intramural, em que o US não é tão acurado – padrão-ouro.^101,102^

#### 7.1.3. Medida do Volume da Placa

Nos últimos anos, os avanços em torno da ultrassonografia ocorreram em grande escala. A criação de sondas vasculares com tecnologia tridimensional e de *softwares* para reconstrução em 3D possibilitaram estudos e recomendações sistemáticas para a padronização e quantificação da placa arterial carotídea na estratificação de risco para a doença aterosclerótica cardiovascular.^26^ Por meio dessa técnica prática e reprodutível, é possível quantificar o volume, caracterizar a anatomia e função da parede arterial, incluindo a caracterização da placa, com resolução espacial aprimorada.^26,98^ A principal vantagem da quantificação 3D é a capacidade de medir uma lesão específica em todos os planos, técnica que possibilita o acompanhamento da progressão da lesão e do tratamento da mesma.

O volume da placa carotídea equivale ao conteúdo aterosclerótico medido em um segmento arterial definido. A importância dessa aferição se dá devido à possibilidade diagnóstica de placas em artérias angiograficamente normais e em lesões carotídeas com menos de 50% de estenose.^98^

A aquisição do volume de placa da carótida pode ser mensurada por meio de dois métodos distintos, de acordo com o equipamento disponível:

1. Protocolo de região única, em que há reconstrução de um segmento específico ou placa única.
2. Protocolo de vaso completo, em que há reconstrução de um conjunto de dados adquiridos ao longo do trajeto examinado.

O volume total da placa, medido de 1,5 cm da artéria carótida comum distal até 1 cm distal à bifurcação, é fator preditor de eventos futuros de DCV.^26,99^ A avaliação ultrassonográfica da EMI e o volume de placa têm sido usados para estratificação de risco e avaliação de terapias antiateroscleróticas. Segundo Wannarong et al.,^99^ a medida do volume de placa e sua progressão são superiores às medidas de EMI em ambas as situações.

De acordo com o estudo de Ball et al.,^98^ o volume de PC em pacientes com sintomas de isquemia cerebral é maior em pacientes nas primeiras semanas da manifestação, quando o risco de AVE também é maior. No entanto, não houve relação significativa entre o volume da placa e a estenose carotídea. Noflatscher et al. demonstraram forte correlação entre o volume total de placa carotídea com fatores de risco cardiovascular (hipertensão, hiperlipidemia, idade, presença de doença cerobrovascular e/ou coronariana) e o número de leitos vasculares acometidos.^100^ No entanto, atualmente, os dados para classificação de volume da placa são limitados, e mais estudos são necessários para estabelecer valores de corte preditores de DCV.^26^

## 7.2. Caracterização da Placa Aterosclerótica pela Angiotomografia e Angiorressonância Magnética

Entre as várias indicações da angio-RM e da angio-TC está a caracterização das placas e da parede arterial por possuírem resolução espacial submilimétrica, com acurácia similar na detecção desses processos quando considerados os equipamentos e técnicas mais modernos disponíveis.^101,102^ Torna-se importante a racionalidade na tomada de decisão de quando indicar uma ou outra técnica, individualizando-se caso a caso, de acordo com as particularidades clínicas. Esses exames, entretanto, não são utilizados para a avaliação de risco cardiovascular e, sim, para pacientes já sintomáticos ou já inicialmente rastreados por outro método, como a ultrassonografia vascular, e para avaliação da gravidade da estenose e extensão da doença. Todavia, esses métodos de imagem são muito úteis para o diagnóstico da dissecção de vasos cervicais e hematoma intramural, onde o US não é tão acurado.

### 7.2.1. Dissecção de Vasos Cervicais

A angio-CT e a angio-RM são úteis no diagnóstico de dissecção arterial cervical (recomendação Classe I – Nível de evidência: C) e são técnicas não invasivas de grande acurácia, que se tornaram métodos de escolha na suspeita de dissecção arterial em lugar da angiografia digital (padrão-ouro).

As técnicas de angio-TC e angio-RM nos equipamentos mais modernos disponíveis demonstram acurácia similar na detecção de dissecções arteriais. Destaca-se, contudo, a maior sensibilidade da RM na demonstração de hematomas murais e sua capacidade superior em diferenciar dissecções agudas e subagudas (caracterizadas pela presença de deoxi ou meta-hemoglogina predominantes no hematoma mural). Um recurso técnico adicional e mais recente (a imagem de parede de vaso por RM – “*vessel wall imaging*”) contribui para essa detecção superior.

## 8. Agente de Realce de Ultrassom na Caracterização da Placa Aterosclerótica

O maior avanço da ultrassonografia após a introdução do modo B e do Doppler foi a introdução de agentes de contraste, ampliando muito o valor desse método e a sua utilização na prática clínica.^106^ Prefere-se utilizar o termo agente de realce de ultrassom (ARUS), ecorrealçador, ao termo agente de contraste, para diferenciar dos contrastes que usam iodo ou gadolínio.^107^

A grande inovação técnica foi a introdução de módulos de imagem específicos para o ARUS nos equipamentos de US com a utilização da harmônica de pulso invertido, possibilitando a visualização direta de sinais emitidos por esses agentes, independentemente de suas velocidades. Devido às características próprias dos sinais das microbolhas (que são fundamentalmente diferentes daqueles provenientes dos tecidos estacionários), são criadas imagens “específicas das microbolhas”, que podem exibir volume e perfusão de parênquimas teciduais com sensibilidade e resolução espacial extremamente elevadas.^108^ A utilização do ARUS abriu novos horizontes no campo da pesquisa em muitas patologias arteriais, uma vez que ele é capaz de fornecer novos conjuntos de dados que podem ser fundamentais no manejo do paciente. Os aspectos a seguir devem ser do pleno conhecimento para sua utilização.

### 8.1. Características e Propriedades dos Agentes de Realce de Ultrassom

O ARUS, ao contrário daqueles agentes empregados para a ressonância magnética (RM) e a tomografia computadorizada (TC) que utilizam as características físicas e químicas das células para o seu efeito, utiliza as características físicas do próprio método ultrassonográfico, ou seja, quanto maior a diferença de densidade entre os meios, maior a reflexão da energia emitida e maior a amplitude do sinal de US. Indiscutivelmente, o meio gasoso é o que promove a maior diferença, correspondendo a um aumento do sinal da ordem de 30 decibéis.

Os ARUS são microbolhas de gás contidas em cápsulas com membrana fosfolipídica que possuem flexibilidade e estabilidade. O agente SonoVue® (Bracco Imaging S.p.A.) é o único produto liberado atualmente no Brasil pela Agência Nacional de Vigilância Sanitária (Anvisa) e pelo rol da Agência Nacional de Saúde Suplementar (ANS). O SonoVue® consiste em microesferas de gás hexafluoreto de enxofre encapsuladas. As microbolhas possuem diâmetro médio de 2,3 µm (tamanho que as impede de atravessar as paredes dos vasos sanguíneos e alcançar o espaço intersticial, não ocupando o espaço extravascular). Por ser um gás lipofílico, tem baixa solubilidade no sangue e não se difunde para fora da cápsula. Essa capa proteica composta de uma camada única de fosfolipídios atua como surfactante, conferindo-lhe estabilidade e flexibilidade ao longo de seu trajeto na macro e microcirculação sanguínea. O SonoVue® é, portanto, considerado um agente integrante exclusivo do pool de sangue e um marcador da circulação sanguínea (propriedade que o distingue dos contrastes utilizados na RM e na TC, que podem atravessar para o espaço extracelular). Após a ruptura da microbolha, o gás é exalado na respiração através dos pulmões em sua quase totalidade, não sofrendo qualquer metabolização hepática ou excreção renal.^109^Assim, não há contraindicação ao uso em pacientes com insuficiência renal, uma vantagem com ampla aplicação em diabéticos, hipertensos, cardiopatas e outras doenças que cursam com insuficiência renal crônica.

### 8.2. Aspectos Técnicos que Influenciam a Obtenção de Imagem com Contraste

Atualmente, a maioria dos fabricantes de equipamentos de US possui *software* específico para estudo com ARUS, o que pode estar incluído na configuração original da máquina ou ser adquirido à parte. Entretanto, mesmo naqueles sem o módulo de imagem específico para contraste, alguns parâmetros podem ser configurados pelo próprio operador. Para a obtenção de melhor resultado durante o estudo contrastado, alguns conceitos e regulagens do equipamento devem obrigatoriamente ser conhecidos.

### 8.3. Índice Mecânico

O comportamento das microbolhas, quando expostas ao US, varia de acordo com a potência de US emitida, ou seja, a amplitude da onda acústica, a qual é denominada de índice mecânico (IM) nos equipamentos. Em estudos sem ARUS, o IM encontra-se na faixa de 1,6 a 1,9; entretanto, sob essa potência acústica, a microbolha invariavelmente entra em oscilação vigorosa e se rompe, gerando dois efeitos indesejados: aumento abrupto da intensidade do sinal com borramento excessivo na imagem e marcada redução da concentração de agente, com consequente encurtamento do tempo de exame. Esse modo de imagem, chamado de “imagem por estimulação acústica”, não necessita de equipamentos com tecnologia específica para contraste, mas, por outro lado, não utiliza todo o potencial do agente, limitando-se à função de ecorrealçador.

Ao reduzirmos o IM para ≤ 0,2, conseguimos não somente manter a integridade das microbolhas, como também fazer com que elas oscilem de forma não linear (compressão inicial seguida de expansão) e entrem em ressonância, emitindo frequências (as chamadas “frequências harmônicas”) diferentes da frequência fundamental emitida pelo transdutor. Os equipamentos dotados dessa tecnologia conseguem filtrar esses sinais emitidos especificamente pelas microbolhas, obtendo um estudo mais duradouro e que destaca o sinal das microbolhas em detrimento dos tecidos, estes praticamente anulados na imagem que aparece como fundo escuro. Essa forma de estudo, chamada também de “estudo com contraste com baixo IM”, permite avaliar de forma contínua o tempo de chegada do (*wash in*) ao local de estudo, o período de realce e a concentração das microbolhas na estrutura-alvo, muito importante para situações como o estudo dos vasos nutridores da parede vascular (*vasa vasorum*), das placas carotídeas, da distribuição capilar (perfusão) renal e de massas em geral.^108^

Um efeito indesejado no estudo contrastado com baixo IM é a limitação da profundidade atingida pela onda de pulso, que sofre maior atenuação à medida que caminha pelos tecidos. Algumas formas de minimizar esse efeito são: adoção de janelas acústicas alternativas que permitam aproximar a estrutura de interesse, utilizar transdutores de banda larga com frequências menores (muitas vezes necessário no estudo das carótidas) e, em último caso, aumentar o IM, tendo como consequência a maior destruição de bolhas no campo proximal.^110^

### 8.4. Ganho de Imagem

Um controle do equipamento que merece atenção no estudo contrastado é o ganho da imagem, que amplifica o sinal recebido durante o pós-processamento no equipamento. O ganho elevado produz uma imagem brilhante e um aumento generalizado no ruído de fundo, obscurecendo o sinal do contraste (uma vez que o nível de saturação do equipamento tenha sido atingido, não haverá margem para aumento do sinal provocado pelo contraste). Durante o estudo com contraste, deve-se, portanto, reduzir o ganho do equipamento até que a imagem fique virtualmente de cor preta, exceto para estruturas altamente ecogênicas. Alguns fabricantes possuem controles de ajuste de ganho para estudos contrastados, que podem facilmente ser ativados e desativados durante o estudo. Quando se realiza um ajuste manual, deve-se ter a menor quantidade de sinais acústicos antes da injeção do agente e entender se esse sinal é provocado por aumento do IM (quando são visualizadas estruturas específicas na imagem) ou do ganho (que provoca aumento generalizado do ruído em toda a imagem).^110^ Em geral, os equipamentos permitem o estudo simultâneo com Modo B e contraste em telas paralelas (lado a lado).

### 8.5. Quantidade de Contraste

A dose do ecorrealçador a ser injetado deve ser previamente estudada pelo examinador. Doses altas provocam inicialmente borramento (saturação) do sinal e atenuação (sombra acústica) das estruturas no campo distal, até que haja queda para concentrações adequadas do nível de contraste. Além disso, não será possível distinguir pequenas diferenças de realce entre estruturas, uma vez que o limite superior da faixa dinâmica (escala de cinza) do equipamento foi ultrapassado.^110^ Uma forma de destacar os diferentes níveis de realce provocados pelo contraste em uma estrutura é ajustando a dose do agente para níveis que permitam a opacificação adequada, sem borramento ou atenuação, e aumentando o nível da faixa dinâmica (*dynamic range*) do equipamento. Doses baixas, por sua vez, não alcançarão o nível de opacificação desejado.

As indicações para uso de contraste em ultrassonografia vascular e especificamente nas carótidas são resumidas no Quadro 7.^111^

**Quadro 7:** – Indicações para o uso de agentes de realce na ultrassonografia vascular nas artérias carótidas e vertebrais.

Aplicação	Classe de recomendação	Nível de evidência
Oclusão x suboclusão	IIa	B
Neovascularização placa	I	B
Dissecção	IIa	B
Inflamação	IIb	B

### 8.6. Diagnóstico de Oclusão e Sub Oclusão

**Figura 8:**
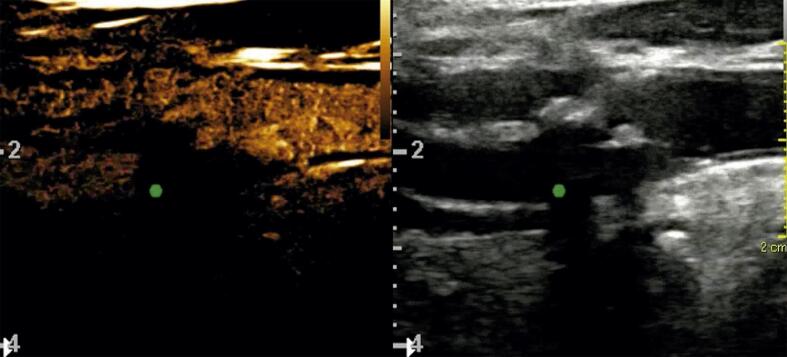
– Utilização do ecorrealçador em placas calcificadas demonstrando estenose > 70% (próxima à suboclusão) na emergência da artéria carótida interna esquerda (circunferência verde). A. Na fase com microbolhas, há passagem do meio de contraste pela placa, sendo observada contrastação após o ponto de estenose (circunferência verde), entre 27 segundos e 34 segundos, após a injeção endovenosa do contraste Sonovuer. No modo B, verificar a dificuldade no local da estenose pela sombra acústica (circunferência verde).

Na suspeita de doença da artéria carótida, a utilização de microbolhas melhora a sensibilidade do US e da técnica Doppler, permitindo distinguir a oclusão da estenose suboclusiva rígida de forma comparável à TC multidetectores com contraste.^111-113^ O US contrastado determina melhor visualização endovascular, caracterizando a geometria detalhada dos segmentos pré-estenóticos, intraestenóticos e pós-estenóticos desprovidos de artefatos ou dependência de angulo^112,113^ (Figura 8).

### 8.7. Avaliação da Neovascularização e Vulnerabilidade das Placas

Essa indicação do ARUS para a avaliação das placas carotídeas baseia-se na premissa de que a placa vulnerável apresenta uma fina cápsula fibrótica que cobre um grande núcleo lipídico necrótico em um processo inflamatório ativo. A presença de neovascularização no interior da placa aterosclerótica é a chave para a identificação da placa sob risco, já que os neovasos servem como porta de entrada para células inflamatórias, lipídios e células vermelhas, aumentando o espaço entre as junções e contribuindo, assim, para o crescimento da placa. Além disso, os vasos neoformados têm um maior risco de ruptura, levando à hemorragia intraplaca e a um rápido crescimento da mesma.^106,112-114^

O ARUS permite uma melhor avaliação da parede do vaso e da superfície das placas. Pelo fato de poder detectar individualmente a passagem das bolhas pelos capilares, essa técnica permite a visualização direta da neovascularização intraplaca, já que as microbolhas são estritamente marcadoras intravasculares.^106^ Portanto, na aterosclerose carotídea, o ARUS não só é capaz de diferenciar oclusão de estenose crítica, como também de realizar uma avaliação qualitativa da placa. As características de placa mais importantes que o ARUS é capaz de identificar são ulceração, neovascularização e presença de infiltrados inflamatórios. Todos esses fatores contribuem para a vulnerabilidade da placa.^106,112^

### 8.8. Dissecção

A utilização do ARUS pode melhorar a acurácia do exame USV com Doppler, entretanto o padrão de referência para o diagnóstico das dissecções vasculares é a RM.^111,115^ Nos pacientes com história de contraindicação ao contraste com gadolínio, destaca-se a importância dessa metodologia.

### 8.9. Inflamação

O ARUS também possui aplicabilidade na avaliação de vasculites dos grandes vasos, como grande diferencial na avaliação das paredes vasculares. Com essa metodologia de utilização das microbolhas, há visualização da borda do lúmen e permite-se a avaliação dinâmica das paredes carotídeas, que é um marcador da atividade da doença.^111,116^

### 8.10 Avaliação de *Stent*

A avaliação evolutiva após colocação de *stent* pode ser efetuada com a utilização de microbolhas.^111,117^ A utilização dessa metodologia permite melhor avaliação intraluminal do *stent*, pois não apresenta artefatos usualmente verificados no estudo com a análise do Doppler espectral. Dessa forma, o estudo com ARUS possibilita avaliação do comprimento e da morfologia das estenoses *intrastent*.

### 8.11 Preparação do Contraste

O SonoVue® é composto por um *kit* que inclui: um frasco ampola com 25 mg de pó liofilizado em uma atmosfera de hexafluoreto de enxofre; uma seringa preenchida com 5 mL de solução de cloreto de sódio 9 mg/mL (0,9%); e um sistema de transferência. O produto é de fácil preparo à beira do leito, seguindo-se as instruções do fabricante. Após transferir o conteúdo da seringa para o frasco com pó, o mesmo deverá ser agitado durante 20 segundos para a formação das microbolhas e para a solução salina transformar-se em uma suspensão com aspecto leitoso (indicando distribuição homogênea das microbolhas). Nesse estado, a suspensão pode ser armazenada por até 6 horas. Se as microbolhas se acumularem na superfície durante o repouso, a solução poderá ser novamente agitada para que as microbolhas readquiram distribuição homogênea antes do uso. A via usual de administração é uma injeção intravenosa em bolus em veia de calibre adequado para punção com agulha de 20 G de diâmetro (preferencialmente em fossa antecubital). Um pequeno volume inicial deve ser administrado, seguido de um flush com 10 mL de solução salina a 0,9% para empurrar o agente de contraste até a veia central (o que ocorre em segundos).

A dose recomendada na maioria das publicações para injeção única nos estudos de USV é de 2,4 mL, podendo variar de 1 a 4,8 mL, a sonda empregada e a sensibilidade do equipamento disponível (lembrando sempre que sondas com frequência mais elevada, como no caso do exame de carótidas, necessitam de doses maiores, no caso 4,8 mL). Os primeiros 10 a 40 segundos após o bolus correspondem à curva de realce do contraste (*wash in e wash out*) e devem ser registrados continuamente para posterior análise. Em alguns casos específicos, como na pesquisa de *endoleaks* tardios pós-colocação de próteses endovasculares aórticas, o tempo de avaliação pode chegar a 5 minutos; nesses casos, clipes menores podem ser registrados. Deve-se ter em mente que, quanto maior o IM, maior a destruição de bolhas e menor o tempo de duração do contraste. Após a ruptura das bolhas, o hexafluoreto de enxofre é rapidamente eliminado pelos pulmões, em cerca de 2 minutos.

SonoVue® é um agente seguro, com baixo índice de complicações. Há relatos de reação anafilática em cerca de menos de 0,0014% dos casos.^111^

### 8.12. Protocolo Básico de Exame de Ultrassonografia Vascular com Contraste de Microbolhas1,16

Definida a indicação de uso de contraste com microbolhas em exame de US vascular, a rotina básica obrigatória requer:

– Repetição e registro de exame de USV padrão do órgão de interesse.
– Garantia de acesso venoso para injeção de solução de contraste com microbolhas (punção de veia periférica preferencialmente).
– Preparação da solução de contraste com microbolhas (SonoVue®) de acordo com as orientações do fabricante do produto.
– Acionamento do modo de imagem específico para contraste no equipamento de US; caso não haja *software* específico, ajuste de IM (< 0,6 e o mais próximo possível de 0,1), ganho de imagem (escurecer o fundo) e escolha de janelas adequadas que reduzam a profundidade do órgão alvo do estudo.
– Administração da solução com contraste, ajustes para reduzir excesso de realce e registro de imagens (clipes) digitais durante os 10 a 60 segundos após bolus inicial; nos casos de exames específicos com maior duração, registrar clipes necessários no decorrer do tempo (que pode alcançar 5 a 8 minutos) para análise posterior.

O exame com contraste de microbolhas é fundamentalmente dinâmico, e a duração do estudo é curta em razão da rápida destruição das microbolhas pelas ondas de US, mesmo quando se utiliza um IM muito baixo. Portanto, o registro em mídia digital dinâmica é essencial para posterior processamento e reavaliação cuidadosa das imagens, garantindo diagnóstico seguro e armazenamento perene dos resultados.

As principais limitações do uso ARUS em USV são inexperiência do examinador, a ausência de *softwares* específicos, o acesso ao produto em unidades da rede pública de saúde e a ausência completa de “janela” ultrassonográfica. As contraindicações clínicas são IAM, doença pulmonar obstrutiva crônica (DPOC) severa, arritmias cardíacas graves e hipersensibilidade ao ARUS (rara).^1,16,111^

## 9. Avaliação da Doença Ateromatosa em Artérias Vertebrais

### 9.1. Introdução

A investigação de envolvimento aterosclerótico nas artérias vertebrais extracranianas através da USV é indissociável do estudo das carótidas. Isso é essencial para o diagnóstico e tratamento das lesões carotídeas severas, bem como para avaliação criteriosa dos riscos da abordagem cirúrgica. Aproximadamente 25% dos acidentes vasculares isquêmicos encefálicos envolvem a circulação posterior, e a doença aterosclerótica corresponde a 20% dos casos.^118^ As placas ateroscleróticas se localizam predominantemente na origem das artérias vertebrais, sendo que na maioria dos casos são extensões de placas das artérias subclávias.^119^ A presença de estenose vertebrobasilar na vigência de um AVE ou AIT envolvendo a circulação posterior aumenta em aproximadamente 33% o risco de recorrência no primeiro mês após o evento inicial.^120,121^

A descrição detalhada da anatomia das artérias do sistema vertebrobasilar pode ser encontrada na diretriz do DIC 2015 que antecede esta atualização.^1^

### 9.2. Avaliação Ultrassonográfica de Vertebrais

Com os recursos técnicos atualmente disponíveis, consegue-se estudar a artéria vertebral em toda a sua extensão, incluindo o segmento intracraniano e a artéria basilar proximal. Recomendamos incluir, na rotina de avaliação, o estudo da origem do vaso (sede mais frequente das estenoses) e os demais segmentos extracranianos.

### 9.3. Metodologia do Exame de Rotina

A posição do paciente é a mesma adotada para o estudo das carótidas. A profundidade do campo de imagem pode variar com a anatomia do pescoço. A escala de cores deve ser reduzida, aumentando-se a sensibilidade de detecção do fluxo em cores.

A metodologia do exame está descrita na recomendação publicada em 2015 pelo DIC.^1^

### 9.4. Parâmetros Normais

Os parâmetros anatômicos e hemodinâmicos de hipoplasia da artéria vertebral estão descritos na publicação de 2015^1^ e demonstrados na Tabela 5.

**Tabela 5:** – Parâmetros anatômicos e hemodinâmicos para a definição de hipoplasia da artéria vertebral

Critérios anatômicos e hemodinâmicos para hipoplasia vertebral
Diâmetro ≤ 2 mm no segmento V2
Redução do componente diastólico do fluxo
Índice de resistência > 0,75
Aumento do calibre da vertebral contralateral (> 4 mm) com velocidades normais

### 9.5. Quantificação da Estenose

#### 9.5.1. Estenose Proximal (V0-V1)

O diagnóstico baseia-se na identificação de turbilhonamento ao *color* Doppler e no aumento de velocidades de fluxo no local da lesão (que nem sempre é visualizado). Em vertebrais com curso tortuoso, pode haver um aumento fisiológico das velocidades. Uma curva espectral amortecida à vazante corrobora a presença de estenose significativa proximal. Em casos com imagem bidimensional de boa qualidade, pode-se identificar a redução luminal do vaso e medir, com o auxílio do *powerangio*, o lúmen residual pelo critério anatômico distal.

Recomendamos a utilização da Tabela 6, adaptada do estudo de Hua et al.,^122^ com valores de corte para a definição dos graus de estenose proximal da artéria vertebral. A VPS na origem do vaso é o parâmetro de maior especificidade para quantificação de estenose vertebral proximal quando comparada aos demais critérios espectrais como índice de velocidade máxima (IVV) e velocidade diastólica final (VDF).

**Tabela 6:** – Valores de corte para velocidades nas estenoses proximais da artéria vertebral (adaptada de Hua et al.**122**)

Estenose	< 50%	50-69%	70-99%
Vmax	≥ 85 cm/s	≥ 140 cm/s	≥ 210 cm/s
IVV*	≥ 1,3	≥ 2,1	≥ 4
Vdf	≥ 27 cm/s	≥ 35 cm/s	≥ 55 cm/s

*IVV: índice de velocidade máxima no ponto da estenose e o segmento V2; VDF: velocidade diastólica final

#### 9.5.2. Estenose Vertebral nos Demais Segmentos (V2-V4)

Quando o local da estenose é identificado ao ultrassom, sua avaliação deve se basear em análise multiparamétrica, como turbulência ao *color* Doppler, aumento localizado das velocidades de fluxo, aumento dos índices de velocidade e amortecimento do fluxo distal, uma vez que não existem tabelas de quantificação das estenoses para esses segmentos.^123^

Nos casos de segmentos não visualizáveis ao exame convencional (segmento V4 intracraniano), os achados são indiretos e correlacionam-se com o nível da estenose e emergência do ramo cerebelar posterior inferior (ACPI). Nas estenoses pré-emergência do ramo cerebelar posterior inferior (pré-ACPI), as curvas espectrais apresentam velocidades reduzidas e padrão de resistência elevada registradas nos segmentos V1-V2. Estenose após emergência do ramo cerebelar posterior inferior (pós-ACPI) não causa alteração de fluxo, pois há desvio para o cerebelo. Nesses casos, o estudo com Doppler transcraniano (DTC) torna-se indispensável para o diagnóstico.

#### 9.5.3. Oclusão de Vertebral

Os achados variam de acordo com o nível da oclusão. O Quadro 8 mostra as possíveis curvas espectrais de acordo com o nível da oclusão. Não raro, uma vertebral ocluída em sua origem pode reabitar em seu segmento distal através de circuitos anastomóticos bem definidos. Essa possibilidade deve ser pesquisada através do estudo dos segmentos extracranianos distais.

**Quadro 8:** – Curvas espectrais de acordo com o nível de oclusão da artéria vertebral. ACPI: ramo cerebelar posterior inferior; PICA: artéria cerebelar inferior posterior.

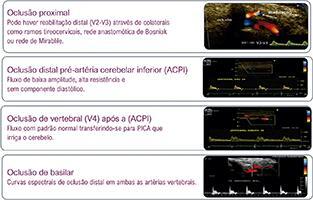

## 9.6. Síndrome do Roubo pela Artéria Subclávia

Estenose hemodinamicamente significativa ou oclusão do tronco braquiocefálico ou de segmento proximal da artéria subclávia (direita ou esquerda) provocam efeito de “roubo” de fluxo da vertebral contralateral (caso a vertebral ipsilateral tenha calibre normal e ausência de doença ateromatosa significativa associada) para suprir a subclávia acometida.^118,124-126^ Nesse caso, a morfologia da curva espectral e a direção da onda de fluxo na artéria vertebral do mesmo lado da subclávia comprometida, em repouso ou após manobra de provocação de hiperemia reativa (compressão do membro superior ipsilateral com manguito de pressão insuflado), permitem avaliar o efeito de “roubo”. Ao contrário das estenoses vertebrais distais, em que o primeiro componente afetado é o componente diastólico, no “roubo”, a primeira alteração ocorre durante a fase sistólica, com breve desaceleração do fluxo sistólico (quase imperceptível para examinadores menos experientes).

O Quadro 9 apresenta a classificação dos diferentes tipos de morfologia das curvas espectrais encontradas nas artérias vertebrais. Em geral, o tipo de roubo correlaciona-se com graus maiores de estenose da subclávia ou tronco braquiocefálico. No caso de estenose grave do tronco braquicefálico, pode haver concomitância de roubo carotídeo que apresentará curva espectral com inversão sistólica do fluxo.

**Quadro 9:** – Classificação dos tipos de roubo de acordo com o padrão de curva espectral.

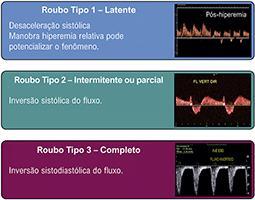

## 10. Doppler Transcraniano na Doença Aterosclerótica Carotídea e Vertebral Extracraniana

O objetivo fundamental do DTC em portadores (assintomáticos ou não) de doença aterosclerótica carotídea ou vertebral extracraniana é investigar o valor preditivo de ocorrência de acidente vascular encefálico isquêmico (AVEi).

As ferramentas de real valor oferecidas pelo DTC são: a) detecção de microembolia encefálica espontânea; b) registro de informações hemodinâmicas durante a monitorização transoperatória (endarterectomia) e em procedimentos endovasculares.^127-129^

### 10.1. Técnicas do Exame

A técnica do DTC varia na dependência da indicação clínica. Na avaliação ambulatorial e transoperatória, a necessidade de monitorização contínua e de longa duração requer uma aparelhagem específica, com capacete ajustável ao crânio e com fixador(es) de transdutor(es). Isso garantirá a captura de evento transitório com informação necessária para a definição da conduta terapêutica mais adequada.^129^

Os aparelhos exclusivamente dedicados ao DTC são “cegos” em razão da ausência de imagem bidimensional e do mapeamento de fluxo em cores (MFC), com óbvia perda de informações anatômicas úteis. No entanto, esses aparelhos permitem a monitorização contínua de fluxo pela possibilidade de contar com o capacete craniano para a fixação do transdutor.

Devemos realizar inicialmente o exame padrão de DTC convencional, com a finalidade de análise da anatomia vascular e busca de possíveis vias colaterais de fluxo.^130-131^ Além disso, pode-se fazer o rastreamento de sítio de estenose intravascular segmentar intracraniana *in situ*, presente em 10% dos casos de AVEi.^134,135^

O exame padrão de DTC “cego” inclui a insonação do fluxo em segmentos de todas as artérias tronculares: circulação anterior, constituída por ramos das carótidas internas direita e esquerda; e circulação posterior, constituída por ramos da artéria basilar, que por sua vez, resulta da confluência das artérias vertebrais direita e esquerda.^136,137^

**Figura 9:**
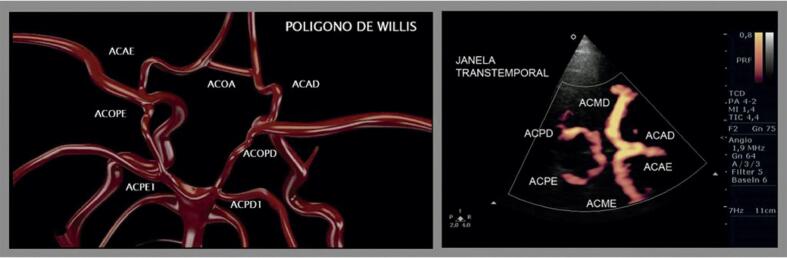
– Polígono de Willis em representação esquemática (9A) e imagem ultrassonográfica com contraste obtida através de janela temporal direita (9B).

Ambas as circulações se conectam através de artérias “comunicantes” (anterior e posteriores direita e esquerda), integrando um circuito conhecido como “polígono de Willis” (Figura 9). Essa arquitetura vascular consiste em eficiente mecanismo automático de colateralização em caso de oclusão em qualquer um dos vasos, impedindo ou atenuando as consequências da isquemia cerebral. Entretanto, variantes anatômicas ocorrem em mais da metade dos indivíduos, justificando as sequelas heterogêneas decorrentes da oclusão de uma mesma artéria em pessoas diferentes. Na circulação anterior, em cada hemisfério encefálico, a carótida interna (ACI) emite um ramo para o globo ocular (oftálmica) e, em seguida, origina as cerebrais anterior (ACA) e média (ACM), responsáveis pela irrigação da maior extensão do órgão. Na circulação posterior, as vertebrais direita e esquerda se fundem em artéria basilar, que se divide em cerebrais posteriores (ACP) direita e esquerda, irrigando o tronco encefálico e a região do cerebelo.

O transdutor com frequência de 2 MHz (ou menos) é um elemento obrigatório para o DTC, pois a localização profunda dos vasos intracranianos exige ondas de US de baixa frequência. A identificação do vaso insonado pelo Doppler “cego” depende de: a) janela ultrassonográfica utilizada; b) posição do transdutor em relação ao crânio (ângulo de incidência do US); c) profundidade do “volume amostral”; d) características das curvas espectrais das ondas de fluxo [morfologia; direção do fluxo em relação ao transdutor; velocidade sistólica máxima (VPS), velocidade diastólica final (VDF) e velocidade média; índices de pulsatilidade e resistência]. Esses parâmetros definem a artéria examinada (Tabela 7), exceto em casos de variantes anatômicas, onde há desvantagem em relação ao Doppler com imagem.

**Tabela 7:** – Critérios para a identificação de vasos intracranianos

Artéria	Janela	Ângulo do transdutor com crânio	Profundidade do vaso	Velocidade média de fluxo	Direção do fluxo
Sifão carotídeo infraselar	Orbital	Perpendicular	55-70 mm	40-50 cm/s	Negativa
Sifão carotídeo genicular	Orbital	Perpendicular	55-70 mm	40-50 cm/s	Positiva negativa
Sifão carotídeo supraselar	Orbital	Perpendicular	55-70 mm	40-50 cm/s	Positiva
Oftálmica	Orbital	Perpendicular	40-60 mm	20 cm/s	Positiva
Carótida interna distal	Temporal	Para baixo	55-70 mm	45 cm/s	Positiva
Cerebral anterior	Temporal	Para cima e anterior	60-70 mm	60 cm/s	Negativa
Cerebral média	Temporal	Perpendicular	35-60 mm	70 cm/s	Positiva
Cerebral posterior (p1)	Temporal	Para baixo e para trás	55-70 mm	40 cm/s	Positiva
Cerebral posterior (p2)	Temporal	Para baixo e para trás	55-70 mm	40 cm/s	Negativa
Vertebral (v4)	Foraminal	Ligeiramente para cima e lateral	55-70 mm	40 cm/s	Negativa
Basilar proximal	Foraminal	Ligeiramente para cima e central	70-120 mm	45 cm/s	Negativa
Cerebelar posterior inferior	Foraminal	Ligeiramente para cima e lateral	40-55 mm	45 cm/s	Positiva

### 10.2. Protocolo Padrão do DTC “Cego” Convencional

Decúbito dorsal e transdutor de 2 MHz suavemente posicionado sobre cada uma das cinco janelas ultrassonográficas clássicas, em sequência livre, para garantir o estudo de fluxo em todas as artérias tronculares intracranianas: a) transorbitais (direita e esquerda); b) transtemporais (direita e esquerda); c) transforaminal.

**a)Janelas transorbitais:** examinar oftálmicas e sifões carotídeos (porções intracavernosas de carótidas internas). Transdutor apoiado sobre o globo ocular, com pálpebra fechada, sem qualquer pressão local (Figura 10A).
  **Figura 10:**
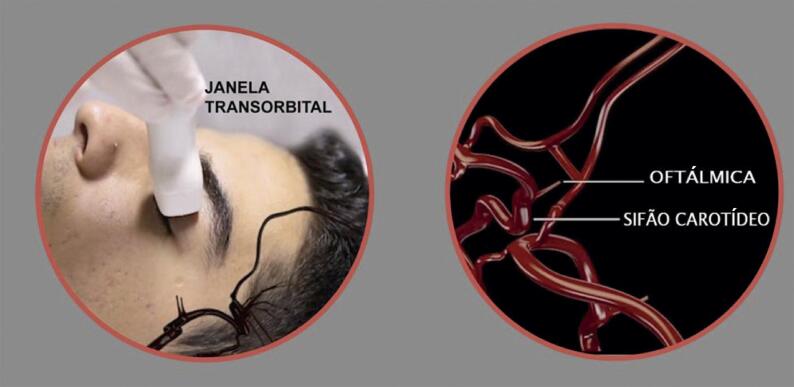
– Doppler transcraniano e janela ultrassonográfica transorbital (10A) e representação esquemática das artérias insonadas (sifão carotídeo e oftálmica – 10B).
**b) Janelas transtemporais:** *s*ituadas acima do arco zigomático (cerca de 1 cm de distância do meato auditivo externo), variam individualmente em extensão e qualidade. Transdutor inicialmente em posição perpendicular ao crânio; em seguida, sutilmente inclinado anterior e posteriormente para a obtenção de imagens referentes às curvas espectrais de fluxo de carótida interna distal, cerebral anterior (A1), cerebral média (M1), topo de basilar e cerebral posterior (P1 e P2) ipsilaterais (Figura 11). As comunicantes (anteriores e posteriores) também podem ser insonadas através dessas janelas ultrassônicas.
  **Figura 11:**
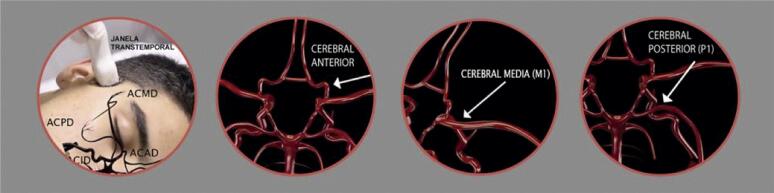
– Doppler transcraniano e janela ultrassonográfica transtemporal (11A); representações esquemáticas de artérias cerebrais anterior (11B), média (11C) e posterior (11D).
**c) Janela transforaminal:** única via de acesso aos fluxos em luzes de segmentos intracranianos das vertebrais (V4) e à origem da basilar (Figura 12), além das artérias cerebelares posteroinferiores (ramos cruciais como via colateral de escoamento de fluxo em casos de oclusão de vertebral acima da emergência dos mesmos).
  **Figura 12:**
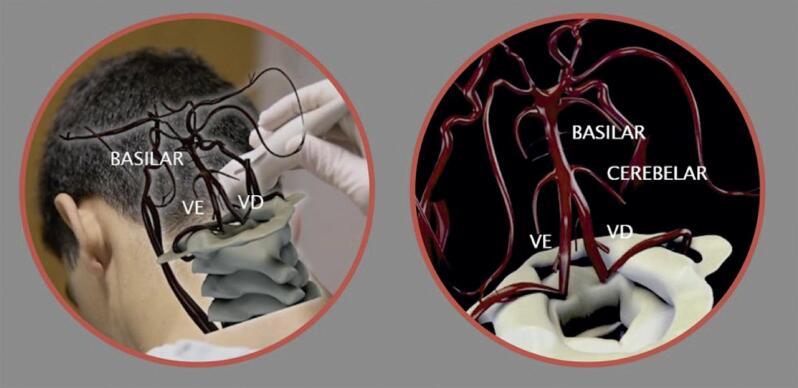
– Doppler transcraniano e janela foraminal (12A); representação esquemática dos de vertebrais e basilar (12B). VD: vertebral direita; VE: vertebral esquerda.
O paciente deve ser posicionado em decúbito lateral, com queixo tocando o tórax para exposição da região occipital (topografia do forame magno), poderá ainda ser colocado sentado no leito ou em uma cadeira, facilitando o posicionamento do examinador. O Doppler pulsátil evidenciará o fluxo afastando-se do transdutor em luzes de vertebrais e basilar; e nas cerebelares posteroinferiores, a direção será inversa.

As curvas espectrais de fluxo registradas nas artérias tronculares intracranianas apresentam morfologia semelhante entre si, diferindo somente nas velocidades próprias de cada vaso e na direção em relação ao transdutor. O padrão é de baixa resistência para todos os segmentos, exceto em oftálmica (única artéria com índice de resistência algo elevado; embora seja ramo da ACI, irriga estruturas extracranianas).

### 10.3. Protocolo padrão de Doppler Transcraniano em Monitorização Contínua

**Figura 13:**
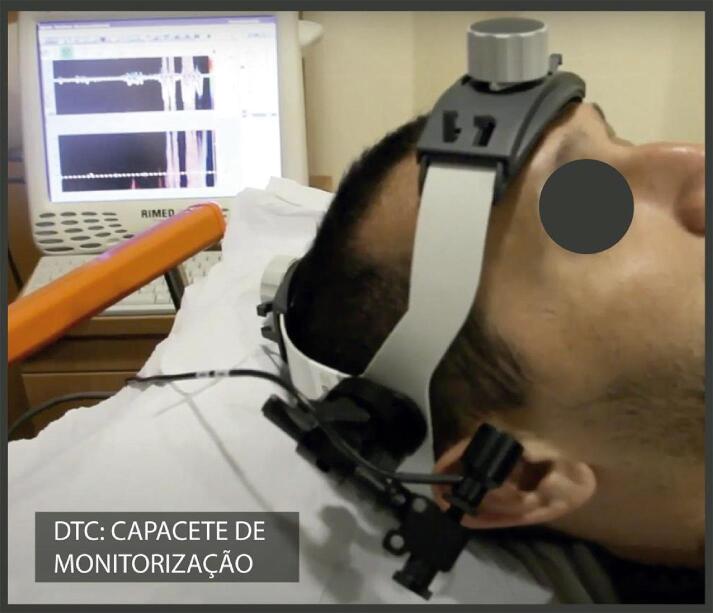
– Capacete ajustável ao crânio para a fixação de transdutores e monitorização contínua de fluxo em cerebrais médias. DTC: Doppler transcraniano.

**Figura 14:**
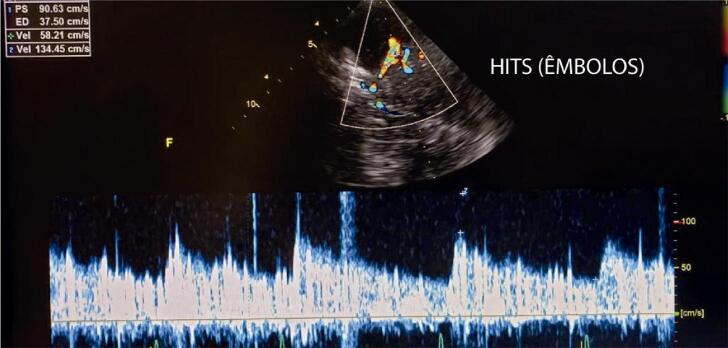
– HITS (High Intensity Transient Signals) indicadores de êmbolos (gasosos ou sólidos).

Capacete ajustável ao crânio, com um ou dois transdutores “cegos” fixados e posicionados em janelas temporais, dirigidos para artérias cerebrais médias (Figura 13). A análise contínua e simultânea de fluxo em ambas as artérias assegura a observação e o registro de ocorrência de êmbolos em tempo real e a contagem de eventos por hora. A microembolia é traduzida como um sinal transitório, de curta duração (inferior a 300 ms), de alta intensidade, impresso na curva espectral de fluxo (Doppler pulsátil) como um traço vertical associado a sonoridade própria, denominado HITS (*high intensity transient signals*) (Figura 14). A contagem do número de HITS e a diferenciação em sólidos e gasosos é importante nas endarterectomias e procedimentos endovasculares carotídeos. O risco de AVEi depende da intensidade dos fenômenos embólicos e pode ser estimado de acordo com o número de HITS catalogados com o DTC.^138,139^

### 10.4. Utilidade clínica do Doppler transcraniano na doença aterosclerótica cervical

#### 10.4.1. Identificação de Pacientes com HITS

A presença de microembolia distalmente a estenose carotídea indica um risco 7,5 vezes maior de AVEi recorrente ou episódio de ataque isquêmico transitório.^140^ Nos pacientes com estenose carotídea sintomática recente (menos de 7 dias) o risco de AVCi recorrente é de 26% em 30 dias.^140^ Portanto, o rastreamento da microembolia permitirá a intensificação da terapia antitrombótica, baseada nos resultados de estudos multicêntricos CARESS (*Clopidogrel and Aspirin for Reduction of Emboli in Symptomatic Carotid Stenosis*)^141^ e CLAIR (*CLopidogrel plus Aspirin for Infarction Reduction*),^142^ ou a antecipação da endarterectomia ou do tratamento endovascular.

A estratificação de risco de pacientes com estenose carotídea assintomática é outra grande utilidade da identificação de HITS. O estudo ACES (*Asymptomatic Carotid Emboli Study*) identificou um risco anual de AVCi ou AIT ipsilateral à estenose de 7,1% nos pacientes com HITS e de 3,0% naqueles sem microembolia.^143^

A incidência de microembolia nas primeiras 24 horas de instalação do AVCi alcança até 49% dos pacientes e reduz progressiva e significativamente após 48 horas.^144-147^ Da mesma forma, nos pacientes com AIT, a presença de HITS está associada à ocorrência de AVEi ou novo AIT.^147^

Durante a endarterectomia, a detecção em tempo real de êmbolos liberados na fase de oclusão carotídea para ressecção da placa aterosclerótica pode ser executada com facilidade e rapidez pela monitorização contínua com DTC, garantindo maior segurança ao procedimento e reduzindo complicações isquêmicas pós-operatórias

#### 10.4.2. Repercussões Hemodinâmicas Induzidas

Especial atenção deve ser dirigida à avaliação de repercussões hemodinâmicas intracranianas, induzidas durante a monitorização de fluxo em cerebrais médias, realizada com aparelho dedicado ao DTC e com capacete de fixação. Nos pacientes sintomáticos, a monitorização deve perdurar por 1 hora, no mínimo; nos assintomáticos, esse tempo deve ser prolongado para 4 horas na intenção de se obter uma melhor acurácia. A análise da autorregulação encefálica e de reserva vasomotora cerebral está entre as informações de maior utilidade.

A autorregulação encefálica (ou pressão de autorregulação) é o mecanismo através do qual o fluxo sanguíneo encefálico se mantém relativamente constante, mesmo diante de variações da pressão de perfusão encefálica (PPE).

Os fatores que influenciam a perfusão encefálica são a PPE e a resistência cerebrovascular (microcirculação). O fluxo sanguíneo encefálico pode manter-se constante durante a variação da pressão arterial média (PAM) se ocorrerem alterações compensatórias na microcirculação (arteríolas). Existem dois métodos para a avaliação do estado de autorregulação encefálica: o estático e o dinâmico. O DTC é um dos métodos mais utilizados para a estimativa de mudanças na perfusão encefálica. A autorregulação dinâmica traduz as alterações transitórias do fluxo sanguíneo encefálico após rápidas mudanças na pressão arterial e pode ser provocada pelo teste do manguito femoral: manguitos de pressão arterial posicionados nas coxas de paciente são mantidos insuflados e, em seguida, são desinsuflados abruptamente com objetivo de provocar hiperemia nos membros inferiores e queda da pressão arterial sistêmica. A autorregulação encefálica fará com que a hipotensão não cause mudança no fluxo sanguíneo cerebral.^148^

**Figura 15:**
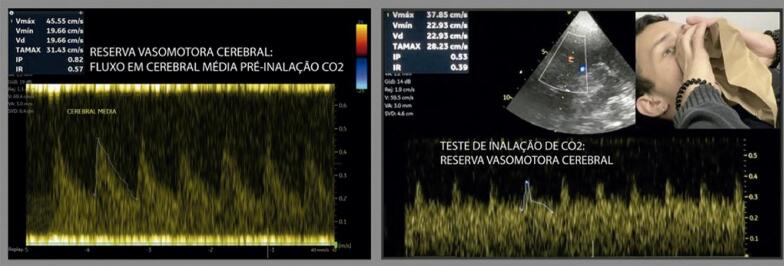
– Teste de avaliação de capacidade de reserva vasomotora cerebral através da inalação de CO_*2*_; redução significativa de velocidades e índice de resistência (15B) em relação ao registro pré-inalação CO_*2*_ (15A).

A reserva vasomotora cerebral (RVC) pode ser estimada através do teste de reatividade cerebrovascular, cujo objetivo é quantificar a capacidade de dilatação de um determinado território arterial, identificando pacientes com estenoses hemodinamicamente críticas e alto risco de falência circulatória encefálica.^149^ Entre os testes de avaliação da reserva microcirculatória, a inalação de CO2 consiste em inspirar, de maneira controlada, uma mistura gasosa enriquecida com CO2.^150^ A hipercapnia gera dilatação de arteríolas e aumento do fluxo sanguíneo (Figura 15); a hipocapnia promove vasoconstricção e redução do fluxo sanguíneo encefálico. Durante a monitorização da velocidade de fluxo sanguíneo em artéria cerebral média, a velocidade pode ser reduzida a 50% abaixo dos valores basais durante hipocapnia, enquanto na hipercapnia, pode elevar-se até 200% acima dos valores de base. Como ponto de corte clínico, recomenda-se que aumentos de fluxo inferiores a 10% devam ser considerados como prejuízo da reserva vasomotora cerebral.

No período intraoperatório, a monitorização de fluxo em cerebrais médias com DTC permite a análise das variações de velocidades de fluxo sanguíneo em resposta ao uso de anestésicos voláteis (causam vasodilatação da microcirculação cerebral e aumento do fluxo sanguíneo encefálico) e agentes hipnóticos (desencadeiam diminuição do fluxo sanguíneo encefálico).^151^

Síndrome de hiperperfusão: no pós-operatório imediato de endarterectomia carotídea, em paciente com estenose severa, o leito cerebral de pequenos vasos (artérias piais e arteríolas) pode apresentar vasodilatação crônica e com perda da capacidade de vasoconstricção após restauração súbita da perfusão com a endarterectomia. Isso resultará em hiperemia cerebral inadequada assim que a pressão normal for introduzida no leito tecidual vasodilatador, e poderá ocorrer morbidade significativa associada com edema, hipertensão intracraniana e hemorragia.^152-155^ Tal mecanismo tem sido descrito também imediatamente ou até 24 a 48 horas após ressecção de malformações arteriovenosas. O DTC pode detectar curvas espectrais com velocidades aumentadas e baixa pulsatilidade e resistência em vasos cerebrais. A medida de velocidades de fluxo em cerebrais médias servirá de guia durante o tratamento até a normalização.

#### 10.4.3. Avaliação de Estenose Vertebral Intracraniana (V4)

**Figuras 16:**
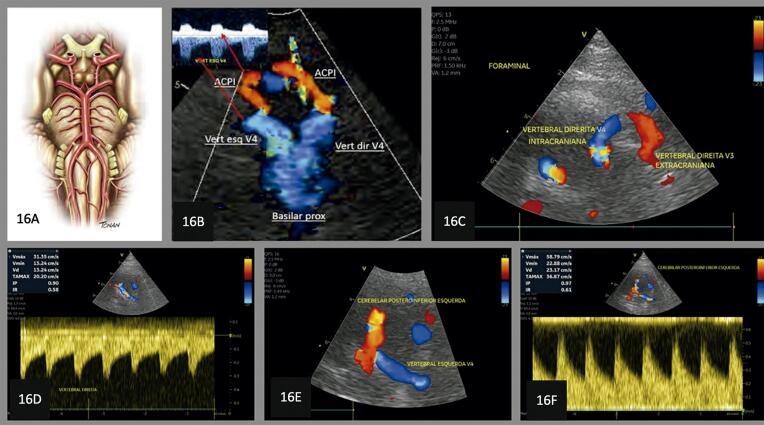
– Anatomia de segmentos intracranianos de vertebrais, basilar e ramos (16A); janela foraminal: mapeamento em cores de fluxo em luzes de vertebrais (V4) e ramos cerebelares posteroinferiores (ACPI – 16B); mapeamento em cores de transição de vertebral V3 (extra) para V4 (intracraniana – 16C); curva espectral de Doppler pulsátil afastando-se do transdutor (V4) (16D); mapeamento em cores de fluxo em ramo cerebelar posteroinferior esquerdo (fluxo em direção ao transdutor – 16E); e curvas espectrais de Doppler pulsátil em ACPI esquerda (16F). ACPI: ramo cerebelar posterior inferior.

O estudo de rotina das artérias vertebrais não deveria restringir-se aos segmentos extracranianos, pois placas estenosantes graves ou até mesmo oclusões em segmentos intracranianos (V4) do sistema vertebrobasilar podem não provocar qualquer anormalidade em curvas espectrais de fluxo em topografia cervical (V0-V3). Se a placa aterosclerótica estiver localizada antes da emergência do ACPI da vertebral intracraniana, as curvas espectrais serão de baixa amplitude e elevada resistência em V1-V3 ipsilateral. Caso a lesão estenosante ou a oclusão da luz situe-se acima do ACPI, poderá haver desvio de fluxo para o cerebelo, e as curvas espectrais serão normais (isso torna o DTC um recurso valioso para o diagnóstico) (Figura 16).

A inclusão do estudo dos segmentos intracranianos do sistema vertebrobasilar requer transdutor setorial com 2,0 MHz (ou menos) com mapeamento de fluxo em cores. Através do forame magno, o US alcançará as artérias e permitirá a visualização de fluxo intraluminal, definindo a anatomia regional. O fluxo em vertebrais afasta-se do transdutor e, em ACPI, tem direção contrária, facilitando a identificação dos vasos.

## 10.5. Recomendações

As recomendações deste grupo de especialistas para o DTC na doença carotídea aterosclerótica estão resumidas no Quadro 4.

1. Em pacientes com placas ateroscleróticas carotídeas ou vertebrais extracranianas, a investigação de “microembolia silenciosa” deve ser realizada com aparelho de DTC “cego” com capacete para fixação dos transdutores no crânio. A monitorização contínua de fluxo em cerebrais médias ou basilar deve ocorrer durante no mínimo 4 horas consecutivas.
2. A avaliação pré-endarterectomia da “reserva vasomotora cerebral” é uma informação valiosa para a redução de risco de isquemia encefálica grave durante cirurgia.
3. A monitorização peroperatória e no mínimo nos 90 minutos imediatos pós-endarterectomia é fundamental para o diagnóstico simultâneo e tratamento precoce de consequências de embolização gasosa ou sólida (partículas de placas ateroscleróticas ou trombos).
4. Recomendamos a inclusão da avaliação dos segmentos intracranianos de vertebrais e de basilar (via janela foraminal) nos exames de rotina de carótidas e vertebrais cervicais de pacientes sintomáticos e sem lesões anatômicas extracranianas que justifiquem a clínica.
