# Supplementary material for: Atualização da Recomendação para Avaliação da Doença das Artérias Carótidas e Vertebrais pela Ultrassonografia Vascular: DIC, CBR, SBACV – 2023
Source: Arq Bras Cardiol. 2023 Nov 14;120(10):e20230695. [Article in Portuguese] doi: 10.36660/abc.20230695 (PMC10699323; doi:10.36660/abc.20230695)
Supplement: Translation

# Translation: Recommendation Update for Vascular Ultrasound Evaluation of Carotid and Vertebral Artery Disease: DIC, CBR and SABCV – 2023

**Authors:** Ana Cristina Lopes Albricker, Claudia Maria Vilas Freire, Simone Nascimento dos Santos, Monica Luiza de Alcantara, Armando Luis Cantisano, Carmen Lucia Lascasas Porto, Salomon Israel do Amaral, Orlando Carlos Glória Veloso, Domingos de Morais, José Aldo Ribeiro Teodoro, Ana Cláudia Gomes Pereira Petisco, Mohamed Hassan Saleh, Marcio Vinícius Lins de Barros, Fanilda Souto Barros, Ana Luiza Dias Valiente Engelhorn, Carlos Alberto Engelhorn, Érica Patrício Nardino, Melissa Andreia de Moares Silva, Luisa Ciucci Biagioni, Adriano José de Souza, Anna Karina Paiva Sarpe, Arthur Curtarelli de Oliveira, Marcelo Rodrigo de Souza Moraes, Miguel José Francisco, Peter Célio Françolin, Carlos Eduardo Rochitte, Rogerio Iquizli, Alair Augusto Sarmet Moreira Damas dos Santos, Valdair Francisco Muglia, Bruno de Lima Naves

**Affiliations:** 1 Brazil Centro Universitário de Belo Horizonte (UniBH), Belo Horizonte, MG – Brazil; 2 Brazil IMEDE – Instituto Mineiro de Ultrassonografia, Belo Horizonte, MG – Brazil; 3 Brazil Universidade Federal de Minas Gerais (UFMG), Belo Horizonte, MG – Brazil; 4 Brazil Empresa Brasileira de Serviços Hospitalares (UBSERH), Brasília, DF – Brazil; 5 Brazil Eccos Diagnóstico Cardiovascular Avançado, Brasília, DF – Brazil; 6 Brazil Hospital Quinta D’Or, Rede D’Or São Luiz, Rio de Janeiro, RJ – Brazil; 7 Brazil Hospital Barra D’Or Rio de Janeiro, RJ – Brazil; 9 Brazil Universidade do Estado do Rio de Janeiro (UERJ), Rio de Janeiro, RJ – Brazil; 10 Brazil Hospital Samaritano, Rio de Janeiro, RJ – Brazil; 11 Brazil Rede UnitedHealth Group (UHG), Rio de Janeiro, RJ – Brazil; 12 Brazil Hospital Pasteur, Rio de Janeiro, RJ – Brazil; 13 Brazil Hospital Américas, Rio de Janeiro, RJ – Brazil; 14 Brazil Hospital de Clínicas Mário Lioni, Rio de Janeiro, RJ – Brazil; 15 Brazil Universidade Estadual de Londrina, Londrina, PR – Brazil; 16 Brazil PRENOTO Clínica Médica e Diagnóstica, Ribeirão Preto, SP – Brazil; 17 Brazil Instituto Dante Pazzanese de Cardiologia, São Paulo, SP – Brazil; 18 Brazil Rede Mater Dei de Saúde, Belo Horizonte, MG – Brazil; 19 Brazil Instituto Fanilda Barros, Vitória, ES – Brazil; 20 Brazil Pontifícia Universidade Católica do Paraná (PUCPR), Curitiba, PR – Brazil; 21 Brazil Faculdade de Medicina do ABC Paulista, SP – Brazil; 22 Brazil Faculdade de Medicina Unoeste, Guarujá, SP – Brazil; 23 Brazil Hospital de Clínicas de Itajubá, Itajubá, MG – Brazil; 24 Brazil Clínica Vascularline, São Paulo, SP – Brazil; 25 Brazil Ecocenter Medicina Diagnóstica, Belo Horizonte, MG – Brazil; 26 Brazil Hospital Ipiranga, São Paulo, SP – Brazil; 27 Brazil Hospital São Luiz Itaim, Rede D’Or São Luiz, São Paulo, SP – Brazil; 28 Brazil Escola Paulista de Medicina da Universidade Federal de São Paulo (EPM/UNIFESP), São Paulo, SP – Brazil; 29 Brazil Hospital Albert Einstein, São Paulo, SP – Brazil; 30 Brazil Instituto do Coração (InCor) da Faculdade de Medicina da Universidade de São Paulo (FMUSP), São Paulo, SP – Brazil; 31 Brazil Hospital do Coração (Hcor), São Paulo, SP – Brazil; 32 Brazil Universidade Federal Fluminense (UFF), Niterói, RJ – Brazil; 33 Brazil Universidade de São Paulo (USP), Ribeirão Preto, SP – Brazil; 34 Brazil Hospital Madre Teresa, Belo Horizonte, MG – Brazil

**Table:** 

Recommendation Update for Vascular Ultrasound Evaluation of Carotid and Vertebral Artery Disease: DIC, CBR and SABCV – 2023	
The report below lists declarations of interest as reported to the SBC by the experts during the period of the development of these statement, 2022/2023.	
Expert	Type of relationship with industry
Adriano José de Souza	Nothing to be declared
Alair Augusto Sarmet Moreira Damas dos Santos	Nothing to be declared
Ana Cláudia Gomes Pereira Petisco	Nothing to be declared
Ana Cristina Lopes Albricker	Nothing to be declared
Ana Luiza Dias Valiente Engelhorn	Nothing to be declared
Anna Karina Paiva Sarpe	Other relationships Funding of continuing medical education activities, including travel, accommodation and registration in conferences and courses, from the brazilian or international pharmaceutical, orthosis, prosthesis, equipment and implants industry: - Sigvaris e Venosan.
Armando Luis Cantisano	Nothing to be declared
Arthur Curtarelli de Oliveira	Nothing to be declared
Bruno de Lima Naves	Other relationships Funding of continuing medical education activities, including travel, accommodation and registration in conferences and courses, from the brazilian or international pharmaceutical, orthosis, prosthesis, equipment and implants industry: - Bayer: Xarelto; Apsen: Dobeven.
Carlos Alberto Engelhorn	Nothing to be declared
Carlos Eduardo Rochitte	Financial declaration A - Economically relevant payments of any kind made to (i) you, (ii) your spouse/partner or any other person living with you, (iii) any legal person in which any of these is either a direct or indirect controlling owner, business partner, shareholder or participant; any payments received for lectures, lessons, training instruction, compensation, fees paid for participation in advisory boards, investigative boards or other committees, etc. From the brazilian or international pharmaceutical, orthosis, prosthesis, equipment and implants industry: - Eventual Speaker Honoraria – Pfizer: Amyloidosis; GE: Cardiovascular Tomography; Edwards: TAVI; Manole: CMR and CCT books. B - Research funding under your direct/personal responsibility (directed to the department or institution) from the Brazilian or international pharmaceutical, orthosis, prosthesis, equipment and implants industry: - V-Plaque (Inclisiran- Novartis): Hcor Institution. Other relationships Any economically relevant equity interest in companies in the healthcare or education industry or in any companies competing with or supplying to SBC: - Blume Medicina Diagnóstica: shareholder.
Carmen Lucia Lascasas Porto	Other relationships Funding of continuing medical education activities, including travel, accommodation and registration in conferences and courses, from the brazilian or international pharmaceutical, orthosis, prosthesis, equipment and implants industry: - Bayer: lectures.
Claudia Maria Vilas Freire	Nothing to be declared
Domingos de Morais Filho	Nothing to be declared
Érica Patrício Nardino	Nothing to be declared
Fanilda Souto Barros	Nothing to be declared
José Aldo Ribeiro Teodoro	Nothing to be declared
Luisa Ciucci Biagioni	Nothing to be declared
Marcelo Rodrigo de Souza Moraes	Nothing to be declared
Marcio Vinícius Lins de Barros	Nothing to be declared
Melissa Andreia de Moraes Silva	Nothing to be declared
Miguel José Francisco Neto	Nothing to be declared
Mohamed Hassan Saleh	Nothing to be declared
Monica Luiza de Alcantara	Financial declaration A - Economically relevant payments of any kind made to (i) you, (ii) your spouse/partner or any other person living with you, (iii) any legal person in which any of these is either a direct or indirect controlling owner, business partner, shareholder or participant; any payments received for lectures, lessons, training instruction, compensation, fees paid for participation in advisory boards, investigative boards or other committees, etc. From the brazilian or international pharmaceutical, orthosis, prosthesis, equipment and implants industry: - Boston Scientific: Watchman FLX.
Orlando Carlos Glória Veloso	Nothing to be declared
Peter Célio Françolin	Other relationships Any economically relevant equity interest in companies in the healthcare or education industry or in any companies competing with or supplying to SBC: - Ensino.
Rogerio Iquizli	Nothing to be declared
Salomon Israel do Amaral	Other relationships Any economically relevant equity interest in companies in the healthcare or education industry or in any companies competing with or supplying to SBC: - Cardiovascular Imaging.
Simone Nascimento dos Santos	Nothing to be declared
Valdair Francisco Muglia	Nothing to be declared

## Contents

1. Class of Recommendation and Level of Evidence 5

2. Summary of the Main Recommendations 5

3. Introduction and Equipment 6

3.1. Introduction 6

3.2. Cleaning and Prevention of Infections 6

4. Intima-media Thickness and

Detection of Carotid Artery Plaques for

Cardiovascular Risk Assessment 8

4.1. Ultrasound Features of Intima-media Thickness and

Carotid Plaque 9

5. Assessment of Carotid Stenosis 9

5.1. Anatomical Criteria 9

5.2. The Role of Computed Tomography Angiography And

Magnetic Resonance Angiography 9

5.3. Velocity Criteria 11

5.4. Technical Considerations for Doppler Assessment 11

5.5. Internal Carotid Artery Stenosis 11

**5.5.1. < 50% stenoses** 11

**5.5.2. > 50% stenoses** 11

**5.5.3. Occlusions and Near Occlusions** 13

5.6. Stenosis of the Common Carotid Artery and

External Carotid Artery 14

5.7. Conditions that Affect Velocity Measurements 14

6. Ultrasound Evaluation after Endarterectomy and

Stent Implantation 14

6.1. Introduction 14

6.2. Test Protocol 14

6.3. Ultrasound Evaluation after Carotid Endarterectomy 15

6.4. Vascular Ultrasound Findings after Endarterectomy 15

7. Morphological Assessment of Carotid Plaques 16

7.1. Investigation of Plaque Morphology 17

**7.1.1. Plaque Morphology** 17

**7.1.2. Characteristics of Atherosclerotic Plaques and Risk of**

**Cardiovascular Disease** 17

**7.1.3. Plaque Volume** 17

7.2. Atherosclerotic Plaque Characterization by Computed Tomography

Angiography and Magnetic Resonance Angiography 17

**7.2.1. Cervical Artery Dissection** 18

8. Ultrasound Enhancing Agents in the

Characterization of Atherosclerotic Plaques 18

8.1. Characteristics and Properties of Ultrasound Enhancing Agents 19

8.2. Technical Aspects that Affect the Acquisition of

Contrast-enhanced Images 19

8.3. Mechanical Index 19

8.4. Imaging Gain 19

8.5. Contrast Agent Dose 20

8.6. Diagnosis of Occlusion and Near-occlusion 20

8.7. Evaluation of Plaque Vulnerability and Neovascularization 20

8.8. Dissection 20

8.9. Inflammation 21

8.10 Follow-up after Stenting 21

8.11 Contrast Preparation 21

8.12. Basic Protocol for Vascular Ultrasound with

Microbubble Contrast Agents 21

9. Evaluation of Atheromatous Disease in

Vertebral Arteries 21

9.1. Introduction 21

9.2. Ultrasound evaluation of vertebral arteries 22

9.3. Methodology of Routine Examination 22

9.4. Normal Parameters 22

9.5. Stenosis Quantification 22

**9.5.1. Proximal Stenosis (V0-V1)** 22

**9.5.2. Vertebral Stenosis in the Remaining Segments (V2-V4)** 22

**9.5.3. Vertebral Artery Occlusion** 22

9.6. Subclavian Steal Syndrome 22

10. Transcranial Doppler in Extracranial Carotid and

Vertebral Atherosclerotic Disease 23

10.1. Imaging Techniques 23

10.2. Standard Protocol for a Conventional “Blinded” Transcranial

Doppler Examination 24

10.3. Standard Protocol for Continuous Transcranial Doppler 25

10.4. Clinical Usefulness of Transcranial Doppler in

Cervical Atherosclerotic Disease 26

**10.4.1. Identification of Patients with HITS** 26

**10.4.2. Induced Hemodynamic Repercussions** 27

**10.4.3. Evaluation of Intracranial Vertebral Stenosis (V4)** 28

10.5. Recommendations 28

References 29

## 1. Class of Recommendation and Level of Evidence

Consensus statements were classified according to Charts 1 and 2, based on standards adopted by the Brazilian Society of Cardiology (SBC).

**Chart 1:** – Class of recommendations according to the standards adopted by the Brazilian Society of Cardiology.

Definition	Grade of recommendation
Conditions for which there is conclusive evidence or, if not, for which group consensus was achieved.	I
Conditions for which there is conflicting evidence and/or divergence of opinions on the usefulness of the method.	II
Evidence or opinion in favor of the method. The majority of authors agrees.	IIa
Safety and usefulness are less well established, and there is no predominance of opinions in favor of the method.	IIb
Conditions for which there is evidence and/or a consensus that the method is not useful.	III

**Chart 2:** – Levels of evidence according to the standards adopted by the Brazilian Society of Cardiology.

Definition	Grade of recommendation
Data obtained from several large, randomized studies showing concurring results and/or a robust meta-analysis of randomized controlled trials.	A
Data obtained from a less robust meta-analysis, a single randomized study, or from nonrandomized (observational) studies.	B
Data obtained from consensual expert opinions.	C

## 2. Summary of the Main Recommendations

A summary of the main recommendations developed by this expert panel is described in Chart 3.

**Chart 3:** – Summary of key recommendations on vascular ultrasound of the carotid system. CCA: common carotid artery; ECA: external carotid artery; EDV: end-diastolic velocity; ICA: internal carotid artery; PSV: peak systolic velocity; VUS: vascular ultrasound.

Expert panel recommendations	Grade of recommendation	Level of evidence	Reference
Major international societies recommend VUS as first choice for the assessment of symptomatic or asymptomatic carotid artery disease.	I	B	1
The entire length of the common, internal, and external carotid arteries should be evaluated bilaterally, as well as the brachiocephalic trunk.	I	B	1
VUS evaluation of carotid stenosis involves hemodynamic criteria such as flow velocity measurements and its ratios by spectral Doppler, in association with anatomical characterization of the plaque and quantification of local stenosis – combined evaluation.	I	B	1-3
PSV is the most well-established hemodynamic criterion for quantification of ICA stenoses and has greater correlation with angiography.	I	B	1-4
EDV and velocity ratios can assist in the diagnosis of stenosis and are of great value in cases where PSV, as an absolute value, may not adequately reflect the degree of stenosis.	I	B	1-4
Near occlusion of the ICA may or may not present with increased flow velocity and, sometimes, the flow may not be detectable. In case of doubt, additional tests should be performed.	I	B	1,5-7
The presence of reduced flow velocity and high resistance on the CCA may indicate the presence of occlusion of the ipsilateral ICA.	I	B	1,6,8,9
Recommendations for grading ICA stenoses should not be used to classify lesions in the CCA or ECA.	I	B	1
Evaluation of CCA stenoses may be based on the ratio between systolic velocities prestenosis and in the stenosis, as well as anatomical quantification.	I	B	1,10,11
ECA stenoses can be quantified according to increases in PSV, as well as by the ratio between PSV in the stenosis and PSV in the CCA.	I	B	1,12-14

The 2015 Guideline recommendations are summarized in Chart 4,^1^ as well as new recommendations on equipment cleaning, carotid plaque definition, media-intimal thickness, grading of stenoses, and plaque morphology.

**Chart 4:** – Reviewed and new recommendations. ASE: American Society of Echocardiography; CCA: common carotid artery; CTA: computed tomography angiography; EDV: end-diastolic velocity; ICA: internal carotid artery; MRA: magnetic resonance angiography; PSV: peak systolic velocity; TCD: transcranial Doppler; US: ultrasound; USEA: ultrasound enhancement agent; VUS: vascular ultrasound.

New or reviewed recommendation	2015 recommendation	Grade of recommendation	2023 recommendation	Grade of recommendation	Level of evidence
**Equipment cleaning**					
NR			The equipment should be cleaned and disinfected according to procedure classification.	I	B
**CP definition**					
R	Focal structure extending at least 0.5 mm into the vessel lumen, and/or measuring more than 50% of the adjacent IME, and/or IME > 1.5 mm	I	2020 ASE classification (26) – Emphasis on the height and focal or diffuse aspect of the CP when grading the risk (< 1.5 mm, between 1.5 and 2.4 mm, ≥ 2.5 mm).	I	B
NR			3D analysis of atherosclerotic burden and atherosclerotic plaque volume	IIa	B
**IMT**					
R	In the absence of CP, the description of IME in the report is at the discretion of the sonographer or at the request of the attending physician.	I	There are no new recommendations. IMT measurement is not routinely recommended in the general.	I	B
**Grading of carotid stenoses**					
R	The local anatomical criterion should be used to characterize < 50% stenoses.	I	There are no new recommendations.	I	B
NR			CTA and MRA – to evaluate the degree of obstruction in symptomatic patients when not obtained by VUS.	I	B
R	Classification of stenoses in deciles in the combined hemodynamic evaluation.	I	There are no new recommendations – Table 2.	I	B
R	PSV is the most accurate criterion.	I	There are no new recommendations – Table 2.	I	B
R	EDV and the ICA PSV/CCA PSV are considered additional criteria in the parametric evaluation.	I	There are no new recommendations – Table 2.	I	B
R	The ICA PSV/CCA EDV ratio is an additional, less accurate criterion and may be used in the parametric evaluation if there is no agreement between the other parameters.	I	There are no new recommendations – Table 2.	I	B
R	Near occlusion – presence of thread-like flow on CFM (string sign or trickle flow).	I	CTA – near occlusion: the artery is partially “collapsed”. Lumen < 1.3 mm, distal ICA caliber < 3.5 mm, diseased ICA/contralateral ICA ratio < 0.87, affected ICA/ipsilateral ECA < 1.27	I	B
R	Occlusion – absence of patency and complete absence of blood flow, as well as high-resistance flow in the CCA and very high preocclusion resistance flow.	I	CTA – occlusion: the artery is completely “collapsed” (string sign).	I	B
**CP morphology**					
R			There have been no changes in classification.	I	B
NR			Use of USEAs (with technical specifications) to identify plaque vulnerability – presence of neovascularization.	I	B
**TCD**					
NR			“Silent” microemboli should be investigated by “blinded” TCD, using a headset to fix the transducer to the patient’s head.	I	B
NR			Pre-endarterectomy assessment of cerebral vasomotor reserve.	I	B
NR			Perioperative monitoring and for at least 90 minutes immediately after endarterectomy.	I	B
NR			Inclusion of the evaluation of the intracranial segments of the vertebral and basilar arteries (via the foraminal window) in routine examination of carotid and cervical vertebral arteries of symptomatic patients without extracranial anatomical lesions that warrant clinical attention justify the symptoms.	I	B

## 3. Introduction and Equipment

### 3.1. Introduction

The ultrasound (US) was introduced in the field of Medicine in the 1940s and has played an important role in the diagnosis of cardiovascular diseases (CVD) since then. The wide applicability, relatively low cost, and reproducibility of the US also made it an established tool in the diagnosis of several other diseases. This Guideline was developed by cardiologists from the Department of Cardiovascular Imaging (DIC) at the Brazilian Society of Cardiology (SBC), angiologists and vascular surgeons from the Brazilian Society of Angiology and Vascular Surgery (SBACV), and radiologists from the Brazilian College of Radiologists (CBR) – who are experts in vascular ultrasound (VUS) – with the aim of supporting the best use of VUS based on the current medical literature, as well as of updating the 2015 Guideline.^1^

The rationale for the use of VUS in the diagnosis of important diseases was based on the recommendations of the 2015, 2016, and 2019 DIC expert panel.^1,15,16^ Other topics included in this update are: transcranial Doppler, US enhancing agents (USEAs), and diagnostic aspects of carotid stenosis by computed tomography angiography (CTA) and magnetic resonance angiography (MRA). However, interested readers should seek more comprehensive and specific publications for further information on these other imaging modalities.

Our aim is to disseminate the best VUS practices among professionals, standardize the interpretation of imaging scans, and promote the best possible use of this noninvasive, widely available, and inexpensive tool.

Equipment, software, probe, and other imaging-related aspects are thoroughly described in the 2015 Guideline.^1^

### 3.2. Cleaning and Prevention of Infections

In addition to the technical and technological requirements of both equipment and the examiner, cleaning the equipment and adhering to infection prevention measures are of utmost importance among professionals. Any diagnostic equipment that gets in contact with a patient poses a risk of infection – although the risk is low, there are reports of transducer contamination, especially using endocavitary probes and in association with central venous access, in addition to bacterial contamination of the US gel.

According to the Spaulding classification,^17^ which determines the level of sterilization/decontamination required for a medical device, VUS procedures are classified as a) critical when the transducer comes into contact with sterile tissue, b) semi-critical when it comes into contact with mucous membranes and nonintact skin (with or without blood contamination), and c) noncritical when there is no contact with sterile tissues, mucous membranes, or nonintact skin. Cleaning and sterilization or high-level disinfection (HLD) are required for critical procedures; cleaning combined with HLD for semi-critical procedures; and low-level disinfection for noncritical procedures.

Most carotid and transcranial diagnostic tests are classified as noncritical. The use of a probe cover (glove, condom, or plastic wrap) is not recommended, but disinfection is required. After the examination, the transducer should be cleaned with a cloth to remove the gel and washed with soap and water. The transducer, the cable, and the keyboard should then be dried before disinfection using quaternary ammonium compounds, alcohols, or phenols. If HLD is required, it is recommended immersing the transducer in a solution of glutaraldehyde, hydrogen peroxide, or peracetic acid for 8-15 minutes. Although the risk of infection is very low, care should be taken to prevent test-related infections, especially in laboratories, clinics, and hospitals where various examinations are performed. Always check with the equipment’s manufacturer which disinfectants can be used, as they may damage the transducer and cable.

## 4. Intima-media Thickness and Detection of Carotid Artery Plaques for Cardiovascular Risk Assessment

After publication of the 2007, 2013, and 2019 Brazilian Guidelines,^1,16,18-20^ the 2004-2006-2011 Mannheim consensus statement,^21^ and the American Society of Echocardiography (ASE) Consensus Statement,^22^ Brazilian experts in VUS joined forces to describe the correct way to measure the intima-media thickness (IMT) and detect atherosclerotic plaques in carotid arteries.

Traditional cardiovascular risk factors are known to be associated with increased IMT.^23-25^ The increase in IMT appears to involve mostly the middle layer, whereas carotid plaque (CP) is related to the thickening of the inner layer and its protrusion into the vessel lumen.^26^

Clinical trials have adopted a wide range of IMT values and, of note, the cutoff point for risk stratification based on numerical values depends on the baseline characteristics of the patient. A recent study by Polak et al.^27^ described a combined percentile score with IMT measurements at the distal common carotid artery (CCA) and proximal internal carotid artery (ICA) that improved cardiovascular risk prediction compared with traditional risk factors, even when the calcium score was added to the study model.

Although IMT measurement is not routinely recommended in the general population, if we consider long-term cardiovascular risk prediction, this may be a valuable measure.^28^ Importantly, in the setting of population aging, cardiovascular risk may be overestimated in older adults with few risk factors, leading to excessive use of medications. The accurate identification of those at actual low risk could result in better clinical outcomes, with economic implications. A recent sub-analysis of the MESA study compared the capacity of ‘negative’ risk markers to downgrade the 10-year cardiovascular risk estimate, such as an IMT value below the 25th percentile.^29^

According to the 2017 Brazilian guideline for dyslipidemia,^20^ atherosclerotic plaque can be defined as IMT > 1.5 mm, so it is important for the vascular sonographer to know how to perform these measurements. Moreover, IMT measurements have a long track record of having been used in research protocols. The technique and interpretation of IMT measurement are described in the document that was the basis for this update.^1^

CP is a manifestation of atherosclerosis and appears to be a stronger predictor of cardiovascular risk than IMT measurement alone. A recent meta-analysis of 11 population-based studies including more than 54,000 patients showed that CP had a higher diagnostic accuracy for the prediction of myocardial infarction (MI) than IMT.^30^ Several publications have studied CP as a prognostic indicator of cardiovascular events, demonstrating its predictive power for the incidence of CVD and coronary events.^31-39^

The I Brazilian Guideline for Cardiovascular Prevention^40^ and the V Brazilian Guideline for Dyslipidemia and Atherosclerosis Prevention^19^ recommend the presence of subclinical carotid atherosclerosis, detected by imaging tests, as a criterion for identifying patients at high risk of coronary events. Furthermore, the Brazilian guidelines as well as the ASE Consensus Statement^22^ recommended that CP should be considered an aggravating factor in patients at intermediate risk.

### 4.1. Ultrasound Features of Intima-media Thickness and Carotid Plaque

On B-mode US, the IMT is characterized by a double-line pattern representing the lumen-intima and the media-adventitia interfaces. The IMT is the distance between the two acoustic interfaces. CP is defined as a focal structure extending at least 0.5 mm into the vessel lumen, and/or measuring more than 50% of the adjacent IMT, and/or an IMT > 1.5 mm.^21^Figure 1 shows a schematic representation of IMT measurement and the 3 definitions of CP, as shown in the 2015 Guideline. Further details on how to obtain and interpret these measurements are described in the base document.

**Figure 1:** – Schematic representation of intima-media thickness and carotid plaque measurements. IMT measurement (A). Three different measurements of the carotid plaque (CP): B) extending ≥ 0.5 mm into the vessel lumen; C) > 50% of the surrounding IMT; D) IMT > 1.5 mm.

In a recent study by Johri et al.,^26^ an IMT ≥ 1.5 mm was considered equivalent to atherosclerotic plaque (type II), especially if the image was diffuse. Type I plaque was defined by an extension < 1.5 mm into the vessel lumen. We understand that type I plaque, as defined by Johri et al.,^26^ corresponds to the first 2 plaque definitions presented in Mannheim’s study.^7^ Therefore, the sonographer should pay particular attention to the classification of type I plaque, using previous scans as a parameter.

## 5. Assessment of Carotid Stenosis

### 5.1. Anatomical Criteria

VUS is able to characterize carotid stenosis through both the velocity criteria and quantification of stenosis using residual diameter measurements, preferably through the transverse plane.

Those who advocate that carotid stenosis should be quantified using the anatomical criteria base their opinion on the following^41^: a) the velocity criteria cannot differentiate narrower degrees of stenosis due to overlap of velocity ranges^42^; b) velocity measurements vary greatly between different devices, leading to discrepant results; c) angle correction causes large inter-observer variation; d) the image quality of B-mode US has significantly improved in recent years.

Members of this expert panel agree that the fundamental criterion for the quantification of carotid stenoses is hemodynamic. The anatomical criteria should be used to quantify stenoses < 50% with no hemodynamic repercussions. After classification using the velocity criteria, it is recommended to inform the degree of stenosis in 10% intervals.^43^

All considerations on the measurement of stenosis using the anatomical criteria are detailed in the 2015 Guideline, and no changes have been made in relation to the previous document.^1^

### 5.2. The Role of Computed Tomography Angiography And Magnetic Resonance Angiography

In patients with focal ischemic neurological symptoms corresponding to the territory supplied by the carotid artery, CTA or MRA is indicated to detect carotid stenosis when the US cannot be performed or yields nondiagnostic results (grade of recommendation: I; level of evidence: C). Both CTA and MRA with postprocessing techniques can provide angiographic images with similar quality to digital subtraction angiography (DSA), allowing stenosis to be measured according to the North American Symptomatic Carotid Trial (NASCET) or European Carotid Surgery Trial (ECST) criteria.^41,44,45^ The degree of carotid stenosis is measured differently (numerical, in percentage) according to each criterion.^46^

Compared with the gold standard technique (DSA), US, CTA, and MRA have the additional benefit of being noninvasive and allowing evaluation of the vascular lumen in the true axial plane (differently from the orthogonal projections of DSA) and some imaging of the arterial wall (not feasible in angiography as it is an exclusively luminographic technique).^47^

Current high-speed, multidetector CTA techniques allow direct evaluation of the carotid lumen diameter and surrounding tissue with high spatial resolution.^49^ Bartlett et al.^48^ demonstrated a linear correlation between millimeter measurements of carotid stenosis and the degree of stenosis estimated by angiography using the NASCET method.^41^ Threshold values of 1.4 to 2.2 mm can be used to evaluate for moderate stenosis (50%-69%) with a sensitivity of 75% and a specificity of 93.8%. A ≤ 1.3-mm residual lumen diameter corresponds to > 70% stenosis and may be used as a cut-off value to diagnose or exclude significant stenosis with a sensitivity of 88.2%, a specificity of 92.4%, and a negative predictive value of 98%.

Of note, in carotid near-occlusion (partial or complete collapse of the ICA distal to the stenosis), the degree of stenosis should not be measured numerically but rather classified as near-occlusion with total collapse, when there is marked reduction of the distal ICA caliber/string sign, or near-occlusion with partial collapse, when there is less marked reduction in the distal IAC caliber.

Cases of near-occlusion with partial collapse are not always clear and evident, therefore there are some imaging criteria that may aid in their correct identification: 1) stenosis caliber < 1.3 mm, 2) distal ICA diameter < 3.5 mm, 3) diseased ICA/contralateral ICA ratio < 0.87, 4) diseases ICA/ipsilateral external carotid artery (ECA) ratio < 1.27, and lower contrast enhancement compared with the contralateral vessel.^49^

Direct measurement of the residual lumen would minimize potential measurement errors compared with the distal ICA lumen, especially in cases of collapse of the walls in severe stenoses (Table 1).

**Table 1:** – Different imaging modalities for quantification of carotid stenosis: comparison of percentage decreases in distal diameter (angiography) between local anatomical criteria (US) and corresponding residual lumen measurements by ultrasonography and computed tomography.

Arteriography (NASCET)	% EST-US Local anatomic (ECST)	Residual lumen (mm) US – B-flow imaging	Residual lumen (mm) Computed tomography
< 20%	< 50%	> 1.5	> 2.2
20-29%	50-55%		
30-39%	58-63%		
40-49%	64-69%		
50-59%	70-75%		1.4-2.2
60-69%	76-81%		
70-79%	82-87%	< 1.5	1.3-1.0
80-89%	88-93%		1.0
90-99%	94-99%		Thread-like flow
Occlusion		Absence of filling	Absence of filling

*The color scale refers to the degree of experience with each method among the scientific community.*

Suwanwela et al.^50^correlated Doppler velocities with residual lumen measurements from surgical pathological specimens removed *en bloc* and suggested that the Doppler criteria has 100% specificity and 96% sensitivity for detecting significant stenosis, defined as residual lumen diameter ≤1.5 mm, in association with significant hemodynamic changes defined by the velocity criteria. In a recent study, Yurdakul et al.^51^ used B-flow imaging, which has better spatial and temporal resolution and less contrast extravasation than color and power Doppler, to demonstrate that a residual lumen diameter < 1.5 mm performs similarly to DSA using the NASCET method to estimate 70%-99% ICA stenosis, with a sensitivity of 93%, specificity of 94%, and accuracy of 94%.

Figure 2 shows residual lumen measurement by B-flow imaging, B-flow angiography, and color Doppler. The comparison of percentage decreases in distal diameter (arteriography) between local anatomical criteria (US) and the corresponding residual lumen measurements by US and CT is shown in Figure 2.

**Figure 2:** – Residual lumen measurements by (A) B-flow imaging, (B) B-flow angiography, and (C) color Doppler. B-flow techniques have less color overlap over the plaque and are the imaging methods of choice for residual lumen measurement.

### 5.3. Velocity Criteria

Several institutions have published criteria for evaluating stenoses by analysis of flow velocities, with some differences between them.^52-58^

Arous et al.^55^ investigated 10 New England institutions and found that they used different duplex ultrasonography Doppler criteria for grading carotid stenoses, which led to significant differences in the number and subsequent costs of interventions. Columbo et al.^59^ examined data from 338 diagnostic centers in the United States relating to two groups: 4,791 patients aged ≥65 years from the Cardiovascular Health Study and 28,483 asymptomatic patients who underwent carotid artery revascularization in the Vascular Quality Initiative registry (www.vqi.org). The authors found great variation in peak systolic velocity (PSV) cut-off points between institutions, both for stenoses greater than 50% and greater than 70%, which led to discrepancies in the diagnosis of stenosis and treatment choices. This study was addressed in an editorial written by Kim and Zierler,^57^ who highlighted the need for standardization of diagnostic parameters for carotid stenosis.

In 2015, the Department of Cardiovascular Imaging of the Brazilian Society of Cardiology (DIC-SBC) published recommendations for the quantification of carotid artery stenosis, including criteria for flow assessment using Doppler associated with anatomical assessment of the plaque. It also divided the degree of stenosis into deciles so that US findings could provide more objective information, assisting in the therapeutic decision.^1^ Thus, as other authors have also suggested, a consensus was reached on the use of a combined approach for the quantification of ICA stenosis.^43,58^

### 5.4. Technical Considerations for Doppler Assessment

Doppler assessment of blood flow velocity should be performed in combination with two-dimensional (2D) evaluation of the plaque. The spectral tracing in the CCA, ICA, and ECA should be measured bilaterally using pulsed Doppler, as well as in any site where B-mode and/or color Doppler suggest the presence of stenosis.^15^ Visualization of the plaque, whether hypo or hyperechogenic, calcified, and with or without acoustic shadowing, is essential for the diagnosis of stenosis, as different hemodynamic conditions can progress with high or low velocities regardless of the presence of stenosis.

Technical considerations for Doppler assessment, such as the correct insonation angle and the location for velocity measurement, are described in the 2015 Guideline.^1^

### 5.5. Internal Carotid Artery Stenosis

This document reviews and updates the criteria published by the 2015 DIC-SBC Guideline. The sequence for carotid stenosis assessment recommended by DIC-SBC is shown in Figure 3.

**Figure 3:** – DIC-SBC recommended sequence for carotid stenosis assessment. PSV: peak systolic velocity.

#### 5.5.1. < 50% stenoses

This document suggests that < 50% stenoses continue to be graded using B-mode imaging, preferably using the transverse plane that provides the best image for measuring lumen reduction.^43,59^

#### 5.5.2. > 50% stenoses

PSV stands out among the criteria for the evaluation of stenoses, and, in the presence of plaque, is considered an important and objective parameter. However, combined analysis with other parameters, such as the EDV and velocity ratios, confers reliability and facilitates the diagnosis (Figures 4 and 5). Furthermore, the use of several parameters allows narrowing the diagnostic possibilities.

**Figure 4:** – Internal carotid artery stenosis of 70% to 79%. A) Common carotid artery flow; B) Internal carotid artery stenosis on color Doppler; C) Internal carotid artery flow in the stenosis; D) Poststenotic turbulent flow in the internal carotid artery.

**Figure 5:** – Internal carotid artery stenosis of > 90%. A) Common carotid artery flow; B) Internal carotid artery flow.

This document supports dividing the degree of ICA stenosis into deciles, according to Table 2, as recommended by the 2015 Guideline.^1^

**Table 2:** – Quantification of carotid stenosis

% Stenosis (NASCET)	PSV cm/s	EDV cm/s	ICA PSV / CCA PSV	ICA PSV / CCA EDV	ICA EDV / CCA EDV
< 50%	< 140	< 40	< 2.0	< 8	< 2.6
50 – 59%	140 – 230	40 – 69	2.0 – 3.1	8 – 10	2.6 – 5.5
60 – 69%		70 – 100	3.2 – 4.0	11 – 13	
70 – 79%	> 230	> 100	> 4.0	14 – 21	
80 – 89%		> 140		22 – 29	> 5.5
> 90%	> 400		> 5.0	> 30	
Near occlusion	Variable – thread-like flow	Variable – thread-like flow	Variable – thread-like flow	Variable – thread-like flow	Variable – thread-like flow
Occlusion	Undetectable flow	Undetectable flow	Not applicable	Not applicable	Not applicable

*The colors represent, from left to right, the most relevant criteria. CCA: common carotid artery; ICA: internal carotid artery; EDV: end-diastolic velocity; PSV: peak systolic velocity.*

The correlation between velocity parameters by VUS and angiography has already been demonstrated by several authors (Table 3).^60-62^

**Table 3:** – Correlation between velocity parameters by VUS and angiography (r-values)

	PSV	EDV	ICA PSV/CCA PSV	ICA EDV/CCA EDV
AbuRahma et al. (2011)	0.81	0.70	0.57	0.54
Petisco et al. (2015)	0.81	0.78	0.81	-----
Braum et al. (2008)	0.825	0.762	0.766	0.643

*CCA: common carotid artery; EDV: end-diastolic volume; ICA: internal carotid artery; PSV: peak systolic volume.*

VUS has good accuracy in identifying > 70% stenoses but the same does not apply for < 50% stenoses, particularly between 50% and 69%.^42,62,63^ Recently, Barlinn et al.^64^ showed that the German Society of Ultrasound in Medicine (DEGUM) criteria also had a lower sensitivity for the evaluation of stenoses between 50% and 69% than between 70% and 99% (sensitivity of 35% and 81% and specificity of 89% and 69%, respectively).

The 2003 Society of Radiologists in Ultrasound (SRU) consensus and the UK Joint Recommendations recommend a PSV cutoff of > 230 cm/s for the identification of > 70% stenoses, and this value was validated by other authors in their institutions.^60,61,62^ AbuRahma et al.^65^ found good accuracy in the 2003 consensus validation but suggest that, for ≥ 70% ICA stenoses, a PSV cutoff of > 230 cm/s should be used in symptomatic patients, whereas a combined approach should be used in asymptomatic patients (PSV > 230 cm/s; EDV > 100 cm/s; ICA PSV/CCA PSV > 4), or a PSV > 280 cm/s.

To diagnose 50%-69% stenoses, the 2003 consensus and the UK Joint Recommendations recommend a PSV between 125 and 230 cm/s; however, some authors found that higher PSV values were better at diagnosing > 50% stenoses. AbuRahma et al.^65^showed better specificity with a PSV ≥ 137 cm/s than with 125 cm/s (91% x 85%) and opted for a PSV of 140 cm/s, which was already used in their institution.^66,67^ A similar value was found in the study by Petisco et al.,^59^ in which a PSV ≥ 141 cm/s had better specificity than a PSV ≥ 125 cm/s (90% x 83%), with similar accuracy. Other PSV values have been described in the literature. The DEGUM and the External Quality Assurance in Laboratory Medicine in Sweden (EQUALIS) reported, respectively, that PSV values > 200 cm/s and 230 cm/s could diagnose ≥ 50% stenoses and PSV values > 300 cm/s and 320 cm/s could diagnose ≥ 70% stenoses.^4,67,68^ Gornick et al.^4^ retrospectively assessed US scans of 167 patients (299 carotid arteries) comparing the 2003 criteria proposed by the SRU consensus with angiography. They observed that PSVs ≥ 180 cm/s had better sensitivity, specificity, and accuracy (93.3%, 81.6%, and 85.2%, respectively) to diagnose ≥ 50% stenoses, as well as the association of a PSV ≥ 125 cm/sec with an ICA/CCA PSV ratio of ≥ 2 (sensitivity 94.3%, specificity 84.3%, accuracy 87.4%) Reinforcing the need for internationally standardized US criteria, recent proposals rely on a combined approach for a more accurate stenosis classification.^69^

In addition to PSV, EDV can also be useful in the diagnosis of > 70% and 80% stenoses. The 2003 SRU consensus suggests an EDV > 100 cm/s as an additional parameter for identifying obstructions > 70%, and other authors have obtained good specificity using this parameter as well.^70,71^ For the diagnosis of > 80% stenoses, an EDV > 140 cm/s has been used for years by the University of Washington, and was shown to have specificity greater than 90% in other studies as well.^62,72^ Arous et al.^73^ demonstrated that a PSV ≥ 450 cm/s or an EDV ≥ 120 cm/s can diagnose ≥ 80% stenoses with an area under curve (AUC) of 0.66, with no significant difference in AUC between EDVs ≥ 120 cm/s and ≥ 140 cm/s (0.657 x 0.653, respectively).

In addition to absolute velocities, velocity ratios – ICA PSV/CCA PSV, ICA PSV/CCA EDV, and ICA EDV/CCA EDV – are also particularly useful, either as an aid in quantifying stenosis or in particular cases where velocities may be altered due to other conditions that may underestimate or overestimate the degree of stenosis. The ICA PSV/CCA PSV ratio is the most used and has been evaluated and recommended by several studies.^2,3,43,72-77^ The ICA PSV/CCA EDV ratio (St Mary’s index) divides > 50% stenoses into deciles,^78^ but has not been much investigated, and there may be overlapping values for different degrees of stenosis. According to some authors,^78-80^ the ICA EDV/CCA EDV ratio can identify > 80% ICA stenoses when greater than 5.5, but has a lower correlation with angiography.^60,62^

Post stenotic flow can assist in the identification of very severe stenoses and stenoses in calcified plaques, with acoustic shadow, when there is turbulent flow after the plaque, significant reduction in velocity (PSV < 30 cm/s), and an increased acceleration time.^43,67^ It is also important to compare the post stenotic flow with the contralateral flow.^81^

#### 5.5.3. Occlusions and Near Occlusions

The diagnosis of carotid near-occlusions is based on the narrowing of the vessel lumen on color/power Doppler, with thread-like flow (string sign or trickle flow); however, it may be associated with high, low, or undetectable velocities, which occasionally hinders the diagnosis. In near occlusions with high PSV in the stenosis, the velocity distal to the stenosis is significantly reduced.^5^

The 2003 SRU consensus recommends differentiating between near occlusion and occlusion based on the examiner’s opinion rather than Doppler velocity parameters. The UK Joint Recommendations and the American Heart Association (AHA) recommend using an additional diagnostic method to differentiate near occlusions from occlusions, such as CTA, MRA, or conventional angiography.^7,61^

Total carotid occlusions are characterized by the absence of patent lumen in gray-scale US and undetectable flow on color, power, and spectral Doppler and with the use of microbubble contrast agents, in addition to the presence of high-resistance flow in the CCA and staccato flow (very reduced and highly resistant flow in the occlusion or before the occlusion) (Figure 6).^3,81^

**Figure 6:** – Total occlusion of the internal carotid artery (ICA). A) Undetectable flow in the ICA on color Doppler; B) Absence of contrast enhancement in the ICA lumen.

In the presence of ICA occlusion, compensatory mechanisms, such as the development of collateral circulation, arise with the aim of preventing cerebral ischemia, but the most important way of collateralization route is through the circle of Willis.

Another source of collateral flow is created from anterograde flow in the distal branches of the ipsilateral ECA that connect to the ophthalmic branch of the ICA, allowing the detection of retrograde blood flow in the ophthalmic artery.^8^ However, this condition is not present in all cases of ICA occlusion because there are different patterns of retrobulbar circulation,^9^ and it is known that hemodynamically significant stenoses (greater than 70% and near occlusions) of the ICA can progress with retrograde flow in the ophthalmic artery.^43^

In cases of CCA occlusion, the ICA may be patent, with anterograde flow from the ECA and its branches.

## 5.6. Stenosis of the Common Carotid Artery and External Carotid Artery

The incidence of isolated CCA stenosis is low, and little is known about the clinical course of these lesions. Patients with isolated CCA stenosis are suspected to experience more hemispheric symptoms, aphasia, and amaurosis fugax.^62^

There is no evidence to support that recommendations for grading ICA stenosis should be applied to the classification of lesions in the CCA or ECA.

This working group recommends that CCA stenoses should be quantified not only using velocity measurements, but also pre and post stenotic velocity ratios > 2 for those greater than 50%, as well as the measurement of lumen narrowing on color/power Doppler and B-mode imaging (Figure 7). It should be noted that assessment of ostial stenoses of the CCA, especially on the left, may be limited.

**Figure 7:** – Common carotid artery stenosis. A) B-mode imaging; B) Color Doppler; C) Power Doppler; D) High PSV in the CCA stenosis; E) Pre stenotic PSV; F) Transverse plane showing significant narrowing of the residual lumen. PSV: peak systolic velocity.

The main criteria described in the literature for quantifying ECA stenoses are summarized in Table 4.

**Table 4:** – Main criteria described in the literature for quantifying external carotid artery stenosis

	External carotid artery stenoses	
**Author**	**ECA PSV**	**ECA PSV/CCA PSV ratio**
Acer et al. (1996)	< 50% ⇢ < 150 cm/s > 60% ⇢ > 250 cm/s	
Paivansalo et al. (1996)		≥ 50% ⇢ ≥ 2
Kronick et al. (2019)	> 50% ⇢ ≥ 200 cm/s with aliasing	
Shmelev et al. (2019)	≥ 50%, without ≥ 50% ICA stenosis ⇢ > 148 cm/s ≥ 50%, with ≥ 50% ICA stenosis ⇢ > 179 cm/s	> 50%, without ICA stenosis ⇢ ≥ 1.45 > 50%, with ICA stenosis ⇢ ≥ 1.89

*CCA: common carotid artery; ECA: external carotid artery; ICA: internal carotid artery; PSV: peak systolic velocity.*

## 5.7. Conditions that Affect Velocity Measurements

Some conditions, whether due to arterial stenosis or local non-vascular reasons, affect spectral analysis measurements. They may be located distally or proximally to the carotid bifurcation or in the contralateral carotid artery – among the first, we underline aortic valve diseases (stenosis or insufficiency), atherosclerotic stenosis, and arteritis involving the aortic arch, branches, and CCA.^82-84^ In addition to valve diseases, other conditions, such as significant left ventricular systolic dysfunction, cardiac arrhythmias, tachycardia, and bradycardia, can alter the waveform in the arterial system, including the carotid arteries, without the presence of stenosis in these vessels.

It should be noted that cardiac alterations generate systemic effects, that is, changes in waveforms in the carotid artery present bilaterally, just as they affect the other arterial beds.^85^

Conditions affecting velocity measurements are detailed in the 2015 DIC Guideline preceding this update.^1^

## 6. Ultrasound Evaluation after Endarterectomy and Stent Implantation

### 6.1. Introduction

Endovascular and conventional carotid interventions are frequently performed, especially for the treatment of atherosclerotic lesions. Follow-up is essential to identify any changes that may interfere with patency after treatment as early as possible and ensure better postoperative results.^86^ Compared with angiography, VUS is known to be inexpensive and to have good accuracy, but there is no consensus on the periodicity of follow-up.^87^

### 6.2. Test Protocol

Follow-up VUS is similar to the diagnostic examination. It is essential to evaluate and describe all findings.

### 6.3. Ultrasound Evaluation after Carotid Endarterectomy

Surgical treatment of carotid stenosis is performed by means of an incision in the anterior wall, removal of the atherosclerotic plaque, and artery repair with or without placement of a patch.

Two of the main concerns after carotid endarterectomy (CEA) is the rate of restenosis and the risk of subsequent stroke^88^, which are fortunately infrequent.^89,90^

Restenosis developed between 6 and 12 months after CEA are usually due to neointimal hyperplasia. Lesions developing after 24 to 36 months tend to represent recurrence of the atherosclerotic process.^91^

AbuRahma et al.^92^ found no significant value for repeating routine VUS after CEA with a patch closure. Bandyk et al.^93^ and Zierler et al.,^86^ on the other hand, believe the benefits of surveillance outweigh the risks and recommend VUS surveillance with a grade of recommendation of 1B.

### 6.4. Vascular Ultrasound Findings after Endarterectomy

The arteriotomy closure sutures may be seen as bright, evenly spaced echoes along the wall of the CCA and ICA in B-mode imaging (Figure 7A). If a patch was used, it can create a dilation at the CEA site of varying dimensions (Figures 7B and 7C). While a vein patch may be indistinguishable in appearance from the wall of the native artery, the dilation and the sutures can help identify its presence. A Dacron patch will appear as a thick, brightly echogenic surface, and a polytetrafluoroethylene patch will typically appear as a bright, double line that represents the thickness of the material and the effects of US penetration.^86^

The diameters of the native vessel, the anastomosis sites, and the enlarged region, if any, should be measured so that they can be followed and compared later.

The main US features and complications after carotid interventions were described and illustrated in the 2015 DIC Guideline. In this update, only 1 change was made in the restenosis criteria. Chart 5 presents a summary of the criteria to reaffirm the definitions and include the update.

**Chart 5:** – Ultrasound features and major complications after endarterectomy. CCA: common carotid artery; ECA: external carotid artery; ICA: internal carotid artery; VUS: vascular ultrasound; EDV: end diastolic velocity; PSV: peak systolic velocity.

Follow-up after endarterectomy	VUS feature
**Disparate calibers:** Occurs immediately after the procedure	Large difference in caliber between the carotid bulb and the distal segment of the internal carotid artery after endarterectomy (common with carotid patch placement)
**“Step”:** Occurs early	Identification of a “step” in the arterial wall at the site of surgical intervention
**Postendarterectomy complications**	
**Aneurysmal dilatation** Typically occurs late	Marked dilation at the site of endarterectomy; may present with associated thrombus
**Occlusive/non-occlusive thrombosis:** Occurs early	Presence of hypoechoic image adhered to the surgical site, with or without a mobile component
**Restenosis:** Occurring: between 3 and 24 months after the procedure – mechanism: neointimal hyperplasia; 24 months – mechanism: atherosclerosis	**Update:** Lumen narrowing on B-mode imaging (transverse and longitudinal planes), with local turbulent flow < 70% stenoses PSV > 300 cm/s; EDV ≥ 125 cm/s, and PSV ICA/CCA ratio > 5
**Poststenting complications**	
**Stent malposition** Occurs immediately after the procedure	Stent not positioned on the site of greater stenosis, with maintenance of turbulent flow
**Inadequate expansion** Occurs between 0 and 24 months	Measurements of diameters at the stent margins and/or body with > 30% residual stenosis
**Restenosis:** Occurring: between 3 and 24 months after the procedure – mechanism: neointimal hyperplasia; 24 months – mechanism: atherosclerosis	**> 50%:** PSV ≥ 220 cm/s and ICA/CCA PSV ratio ≥ 2.7 **> 80%:** PSV ≥ 340 cm/s and ICA/CCA PSV ratio ≥ 4.15 **Types:** I: focal hyperplasia on the stent margins (< 10 mm) II: focal hyperplasia intrastent (< 10 mm) III: diffuse hyperplasia (> 10 mm) IV: proliferative diffuse hyperplasia (> 10 mm) extending beyond the stent margins V: stent occlusion
**Stent fracture/torsion**	VUS is not the method of choice Should be suspected when there is significant calcification and/or restenosis with abnormal radiography
**Effect of stent on CEA**	May lead to stenosis at the origin of the CEA, with turbulent flow (the flow passes through the stent mesh)

Although most studies consider > 70% stenosis after CEA as a criterion for restenosis severity, this cutoff point varies in the literature. Thus, further studies are needed to standardize the criteria for US velocities in restenosis after CEA. However, velocity differences found in CEA with or without the use of a patch and the possibility of disparate calibers after CEA should be considered.

We recommend the recent criteria by Bandyk et al.^93^ for grading > 70% stenoses after CEA (PSV > 300 cm/s, EDV > 125 cm/s, and an ICA/CCA PSV ratio > 5). For VUS surveillance, according to the same authors, we recommend intervals of 1, 3, and 12 months after the procedure.

## 7. Morphological Assessment of Carotid Plaques

The morphology of the atherosclerotic plaque has been increasingly studied in the evaluation of carotid atherosclerosis. Conventionally, the degree of carotid stenosis has always had the most prominent role in carotid and vertebral imaging studies, as it is the most used parameter in the decision-making process for CEA and carotid stenting. However, for over 2 decades, morphological and histopathological aspects linked to atherosclerotic plaque instability have been studied, that is, plaques with the same degree of stenosis do not necessarily have the same ischemic potential for thromboembolic events. Identifying which plaque would be more unstable or vulnerable may play a key role in therapeutic decision.

The presence of a CP with a lipid-rich necrotic core, carotid intraplaque hemorrhage, and ulceration in patients with recurrent strokes and non-significant stenoses may require surgical intervention or intensive medical therapy according to the best medical practices.^94^ The definition of atherosclerotic plaque is described in the second part of this document (Figure 1) and remains unchanged from the one in the 2015 Guideline.^1^

### 7.1. Investigation of Plaque Morphology

The characterization of plaque morphology plays an important role in the occurrence of strokes and may be an important predictor of ischemic events. The investigation of characteristics associated with an increased risk of ischemic events demonstrates an effort to identify plaque-related parameters that, together with the degree of stenosis, can more accurately predict the presence of vulnerable plaques and the associated risk of these events. However, the US has some characteristics that limit its role in this investigation. Other methods have not yet been routinely incorporated in this assessment, as there is no fully established evidence that it improves risk stratification.^95,96^

#### 7.1.1. Plaque Morphology

Plaque morphology should be described in the VUS report, as recommended in the 2015 DIC Guideline, using the parameters on Chart 5.^1^ The characterization of the atherosclerotic plaque may help predict the progression of the degree of stenosis and clinical events. Hypoechogenic, heterogeneous, and irregular plaques are risk markers for events such as stroke and transient ischemic attack (TIA).

In this document, we updated the value of some characteristics of atherosclerotic plaques and the risk of cardiovascular disease (CVD), assessment of plaque volume, and data from CTA and MRA.

#### 7.1.2. Characteristics of Atherosclerotic Plaques and Risk of Cardiovascular Disease

Herr et al. used a method similar to grayscale median analysis to assess the severity of CVD and risk of cardiovascular events in patients who had recently undergone coronary angiography. Increased echogenicity of CP (fibrous and/or calcium-like tissue) was correlated with increased coronary artery disease, and a combination of plaque height, percent calcium, and/or percent fat increased the risk of cardiovascular events. The study highlights the possibility of using CP composition on US for risk stratification (Chart 6).^97^

**Chart 6:** – Summary of atherosclerotic plaque characterization and cardiovascular risk. CTA = computed tomography angiography; MRA = magnetic resonance angiography; MRI = magnetic resonance imaging; US = ultrasound. *This parameter does not have good accuracy in perioperative testing and is reduced in the presence of calcium and stenotic plaques.

Plaque characteristic	Definition	Clinical risk/reference
**Echogenicity**	*Type I:* uniformly echolucent *Type II:* predominantly echolucent *Type III:* predominantly echogenic *Type IV:* uniformly echogenic *Type V:* calcified	Types I and III plaques are associated with a higher risk of stroke, whereas types IV and V are the most stable.^1^
**Location**	Describe in which segment of the carotid system the plaque is located: common carotid, bifurcation, proximal and middle external and internal branches.	
Surface*	*1. Regular:* < 0.4 mm in depth *2. Irregular:* 0.4 to 2.0 mm in depth *Ulceration:* De Bray – concavity and length > 2.0 mm, with a well-defined back wall and reverse flow within the concavity on color Doppler Muraki – clear concavity and base echogenicity less intense than on the adjacent wall *3. With or without a mobile component:* describe size if mobile component is present.	Irregular and ulcerated plaques are associated with an increased risk of events.^1^
**Intraplaque hemorrhage**	Anechoic area close to the plaque surface with an intact fibrotic cap.	Vulnerability marker due to its significant association with cerebrovascular events; occurs in plaques with and without hemodynamic compromise and appears to be caused by disruption of intraplaque neovascularization or of the atherosclerotic plaque itself.^1,103-105^
**Plaque volume**	CPV is the equivalent of atherosclerotic burden measured within a defined length of artery by 3D imaging and allows monitoring of lesion progression and treatment.	Total CPV, measured from 1.5 cm distal to the CCA to 1 cm distal to the bifurcation, is a predictor of future cardiovascular events.^26,98,99^
**CTA and MRA evaluation**	The advantage of CTA and MRA is that they have submillimeter spatial resolution, but they are not used for cardiovascular risk assessment. – Vessel wall imaging: novel technical resource for the diagnosis of intramural hematomas and arterial dissection by MRI.	Very useful for the diagnosis of acute and subacute cervical vessel dissection and intramural hematoma, for which the US is not as accurate – gold standard.^101,102^

#### 7.1.3. Plaque Volume

In recent years, advances in US technology have occurred at large scale. The creation of three-dimensional (3D) vascular probes and software for 3D reconstruction allowed the conduction of studies and elaboration of systematic recommendations for standardization of the quantification of carotid arterial plaque for the purposes of CVD risk stratification.^26^ This practical and reproducible technique allows quantifying the volume and characterizing the anatomy and function of the arterial wall, including the plaque, with improved spatial resolution.^26,98^ The main advantage of 3D quantification is the ability to measure a specific lesion in all planes, a technique that allows monitoring the progression of the lesion and its treatment.

CP volume (CPV) is the equivalent of atherosclerotic burden measured within a defined length of artery. This measurement is important because it can assist in the diagnosis of plaques in angiographically normal arteries and in carotid arteries with < 50% stenosis.^98^

CPV may be measured using 2 different approaches, depending on the equipment available:

1. Single-region protocol, in which a specific segment or only one plaque is reconstructed;
2. Full-vessel protocol, in which a dataset acquired along the length of the vessel is reconstructed.

Total CPV, measured from 1.5 cm distal to the CCA to 1 cm distal to the bifurcation, is a predictor of future CVD events.^26,99^ US evaluation of IMT and plaque volume has been used in risk stratification and for the evaluation of antiatherosclerotic therapies. According to Wannarong et al.,^99^ the measurement and progression of CPV are superior to IMT in both situations.

In the study by Ball et al.,^98^ CPV was higher in patients with symptoms of cerebral ischemia during the first weeks of symptoms, when the risk of stroke is also higher. However, there was no significant relationship between CPV and carotid stenosis. Noflatscher et al. demonstrated a strong correlation between total CPV and cardiovascular risk factors (hypertension, hyperlipidemia, age, presence of cerebrovascular and/or coronary disease), as well as the number of affected vascular beds.^100^ However, current data for plaque volume classification are limited, and further studies are needed to establish predictive cutoff values for CVD.^26^

## 7.2. Atherosclerotic Plaque Characterization by Computed Tomography Angiography and Magnetic Resonance Angiography

Among the various indications for CTA and MRA is the characterization of plaques and the arterial wall, as they have submillimeter spatial resolution, with accuracy in detecting these processes similar to the most modern equipment and techniques available.^101,102^ The decision on whether to indicate one method or the other should be individualized, according to the clinical particularities of each patient. However, these tests are not used to assess cardiovascular risk, but rather for patients who are asymptomatic or who were initially screened by another method, such as USV, and to assess the severity of the stenosis and the extent of the disease. These imaging methods are very useful for the diagnosis of cervical artery dissection and intramural hematoma, for which the US is not as accurate.

### 7.2.1. Cervical Artery Dissection

CTA and MRA are noninvasive and highly accurate methods that can assist in the diagnosis of cervical artery dissection (grade of recommendation: I; level of evidence: C) and have supplanted digital angiography (gold standard) as the method of choice for suspected arterial dissection.

In the most modern equipment available, CTA and MRA techniques showed similar accuracy in detecting arterial dissections. However, MRI has greater sensitivity for demonstrating mural hematomas and greater capacity for differentiating between acute and subacute dissections (characterized by a predominance of deoxy or methemoglobin in the mural hematoma). Vessel wall imaging, an additional and more recent technical resource, contributes to the superior detection capacity.

## 8. Ultrasound Enhancing Agents in the Characterization of Atherosclerotic Plaques

One of the greatest advances in US technology after the introduction of B-mode imaging and Doppler US are the USEAs, which significantly increased the value and use of US in clinical practice.^106^ The term USEA/echo enhancer is preferred over the term contrast agent to differentiate it from gadolinium and iodinated contrasts.^107^

The great technical innovation was the introduction of contrast-specific imaging modes on US scanners with the use of pulse inversion harmonics, allowing direct visualization of signals emitted by contrast agent microbubbles independently of their velocity. Specific characteristics of microbubble signals, which are fundamentally different from those of static tissue, allow the creation of microbubble-specific images that can display blood volume and parenchymal perfusion with extremely high sensitivity and spatial resolution.^108^ The use of USEAs has opened new horizons in the study of several arterial diseases by providing new sets of data that can be fundamental in patient management. Essential information for the use of USEAs is described below.

### 8.1. Characteristics and Properties of Ultrasound Enhancing Agents

Unlike MRI and CT contrast agents, which use physical and chemical properties of cells to generate their effect, USEAs use the physical properties of the US itself, that is, the greater the difference in density between the two media, the greater the reflection of emitted energy and the larger the amplitude of US signal. Unquestionably, the gaseous medium provides the greatest difference, corresponding to a signal increase of approximately 30 decibels.

USEAs consist of gas-filled microbubbles encapsulated within a phospholipid shell that is flexible and stable. SonoVue® (Bracco Imaging S.p.A.) is the only USEA currently approved for use in Brazil by the National Health Surveillance Agency (Anvisa) and the National Regulatory Agency for Private Health Insurance and Plans (ANS). SonoVue® consists of encapsulated microspheres of sulfur hexafluoride gas. The microbubbles have a mean diameter of 2.3 µm, which prevents them from crossing blood vessel walls and reaching the interstitial space. As a lipophilic gas, it has low blood solubility and does not spread outside the capsule. This protein shell composed of a single layer of phospholipids acts as a surfactant, providing stability and flexibility while it travels along the macro and microcirculation. Therefore, SonoVue® is considered a real blood-pool contrast agent and a marker of blood circulation—this property distinguishes it from MRI and CT contrast agents, which can cross into the extracellular space. After the microbubble ruptures, the gas is almost entirely exhaled via the lungs, without undergoing liver metabolism or renal excretion.^109^Thus, there is no contraindication to its use in patients with renal failure, which is extremely advantageous for patients with diabetes, hypertension, heart disease, and other diseases that progress with chronic renal failure.

### 8.2. Technical Aspects that Affect the Acquisition of Contrast-enhanced Images

Currently, most US device manufacturers have a dedicated software for imaging studies with contrast, which may be included in the original device configuration or purchased separately. However, even machines without a dedicated USEA mode have some parameters that can be configured by the operator. Some concepts and adjustments of the US machine that the operator should know in order to obtain the best imaging results are described below.

### 8.3. Mechanical Index

The signals obtained from microbubbles are dependent on the transmitted US power, that is, the amplitude of the acoustic wave (which is shown on the machine screen as the mechanical index [MI]). In non-USEA examinations, the MI ranges from 1.6 to 1.9; however, with this acoustic power, the microbubbles oscillate violently and rupture, leading to two undesired effects: a sudden increase in signal intensity with an excessive blurring of the image, and a significant reduction in contrast concentration, consequently reducing the examination time. This imaging mode, called “imaging by acoustic stimulation”, does not require machines with a dedicated contrast agent mode but, on the other hand, does not take full advantage of the contrast agent’s potential and is limited to the function of echo enhancer.

By reducing the MI to ≤ 0.2, the microbubbles remain intact and begin to oscillate in an asymmetric manner (initial compression followed by expansion) until they become resonant and emit different frequencies (known as harmonic frequencies) from the fundamental frequency of the transducer. Equipment suitable for this technology can filter signals transmitted specifically by microbubbles, allowing for a longer examination with a more enhanced microbubble signal compared with surrounding tissue, which is practically null (dark background). This imaging mode, also known as low-MI imaging, allows continuous assessment of time of contrast arrival in the region of interest (wash in), enhancement duration, and microbubble concentration in the target lesion, which is very important in cases such as imaging of the vasa vasorum, CPs, distribution of renal capillaries (perfusion), and masses in general.^108^

One major limitation of low-MI imaging is reduced depth of penetration, as the US wave becomes more attenuated while traveling through the tissue. Some solutions include selecting different acoustic windows that bring the target lesion closer to the nearfield, using wide-band transducers with lower frequencies (often necessary in carotid artery imaging), and, if penetration is still insufficient, increasing the MI, which has the disadvantage of increasing microbubble destruction in the nearfield.^110^

### 8.4. Imaging Gain

A noteworthy machine setting in imaging studies with contrast is imaging gain, which amplifies the signal received during postprocessing. High gain settings produce a bright image with enhanced background noise, which may obscure contrast signal (once the machine’s saturation level has been reached, contrast agent signal intensity can no longer be increased). During the examination, gain settings should therefore be reduced until the image becomes virtually black, except for highly echogenic structures. Some manufacturers provide automatic gain adjustment settings that can easily be turned on and off during the examination. When manually adjusting the gain settings, there should be the least amount of acoustic signal before injection of the contrast agent, and it is important to understand whether the signal is caused by an increase in MI (tissue structures become visible on the image) or gain (widespread noise increase over the whole image).^110^ In general, US machines allow simultaneous assessment with B-mode and contrast agents, on parallel screens (side by side).

### 8.5. Contrast Agent Dose

The USEA dose to be injected should always be previously assessed by the examiner. High doses initially blur the signal (saturation) and attenuate (acoustic shadowing) structures in the distal field until contrast concentrations drop to an adequate level. In addition, small differences in enhancement will no longer be distinguishable, as the upper limit of the machine’s dynamic range (grayscale) has been exceeded.^110^ One way of distinguishing different enhancing levels in a structure is to adjust the USEA dose to allow adequate opacification, with no blurring or attenuation, and increase the dynamic range of the machine. On the other hand, low doses will not reach the desired opacification level.

Indications for the use of contrast agents in VUS, and specifically for carotid arteries, are summarized in Chart 7.^111^

**Chart 7:** – Indications for the use of contrast agents in vascular ultrasound for carotid and vertebral arteries.

Application	Grade of recommendation	Level of evidence
Occlusion x near-occlusion	IIa	B
Plaque neovascularization	I	B
Dissection	IIa	B
Inflammation	IIb	B

### 8.6. Diagnosis of Occlusion and Near-occlusion

In suspected carotid artery disease, the use of microbubbles improves the sensitivity of Doppler US and can distinguish occlusion from tight near-occlusive stenosis, comparable to contrast-enhanced CTA.^111-113^ Contrast-enhanced US improves endovascular visualization, characterizing the geometry of prestenotic, intra-stenotic and poststenotic segments without artifacts or angle dependence^112,113^ (Figure 8).

**Figure 8:** – Echo enhancer in calcified plaques demonstrating > 70% stenosis (near occlusion) at the beginning of the left internal carotid artery (green circle). A. When using microbubbles, the contrast medium passes through the plaque, and contrast is observed beyond the stenosis (green circle) between 27 and 34 seconds after intravenous injection of SonoVue®. In B-mode imaging, check difficulty at the site of stenosis according to the acoustic shadow (green circle).

### 8.7. Evaluation of Plaque Vulnerability and Neovascularization

The indication of USEAs for the evaluation of CPs is based on the premise that vulnerable plaques have a thin fibrous cap covering a large lipid necrotic core in an active inflammatory process. Intraplaque neovascularization is the key to detecting vulnerable plaques, as the neo vessels serve as a port of entry for inflammatory cells, lipids, and red blood cells, increasing gap junctions and contributing to plaque growth. In addition, the neo vessels are at increased risk of rupture, causing intraplaque hemorrhage and rapid plaque growth.^106,112-114^

USEAs allow for a better assessment of the vessel wall and plaque surface. Because they can detect individual microbubbles passing though the capillary system, this technique allows direct visualization of intraplaque neovascularization, as microbubbles are strictly intravascular markers.^106^ Therefore, in carotid atherosclerosis, USEAs are not only capable of differentiating between occlusion and critical stenosis, but also of performing qualitative plaque assessment. The most important plaque features that the USEAs can identify are ulceration, neovascularization, and the presence of inflammatory infiltrates, all of which contribute to plaque vulnerability.^106,112^

### 8.8. Dissection

MRI is considered the gold standard for the diagnosis of vascular dissections. However, USEAs can improve the accuracy of US Doppler^111,115^ and are an important alternative for patients with contraindication to gadolinium contrast.

### 8.9. Inflammation

USEAs can also be used to assess large-vessel vasculitis, particularly within the vascular walls. They provide visualization of the lumen border and allow dynamic assessment of carotid wall vascularization, which is a marker of disease activity.^111,116^

#### 8.10 Follow-up after Stenting

Follow-up after stenting can be performed using microbubbles.^111,117^ USEAs improve intraluminal stent evaluation because they have fewer artifacts compared with spectral Doppler. Therefore, imaging studies using USEAs allow depiction of the length and morphology of the stenosis.

## 8.11 Contrast Preparation

SonoVue® is a kit including 1 vial containing 25 mg of lyophilized powder in a sulfur hexafluoride atmosphere, 1 prefilled syringe containing 5 mL of sodium chloride 9 mg/mL (0.9%) solution, and 1 transfer system. The USEA is easy to prepare at the bedside, following the manufacturer’s instructions. After emptying the contents of the syringe into the vial, shake the vial for 20 seconds to form microbubbles and obtain a white milky homogeneous liquid, which indicates that the microbubbles are homogeneously distributed. In this state, the suspension can be stored for up to 6 hours. If microbubbles accumulate on the surface during rest, shake the vial again until microbubble distribution becomes heterogenous again. The usual route of administration is by a bolus intravenous injection using a needle catheter of at least 20G, preferably in the antecubital fossa. A small volume should be administered initially, followed by a flush of 10mL of 0.9% saline to push the contrast agent into the central vein (which happens in seconds).

In VUS studies, the most recommended dose for a single injection is 2.4 mL, ranging from 1 to 4.8 mL according to the target organ, the probe used, and the sensitivity of the machine available. It should be noted that probes with higher frequencies need higher doses, in this case, 4.8 mL. The first 10 to 40 seconds after bolus injection correspond to the time-intensity curve contrast enhancement (wash in and wash out) and should be continuously recorded for later review. In some cases, such as in the investigation of late endoleaks after aortic stenting, the examination may reach up to 5 minutes, and shorter video clips may be recorded. The examiner should bear in mind that the higher the MI, the greater the degree of bubble disruption and the shorter the duration of contrast. After the bubbles disrupt, sulfur hexafluoride is quickly excreted by the lungs (approximately 2 minutes).

SonoVue® is a safe contrast agent with a low rate of complications. Anaphylactic reactions have been reported in approximately < 0.0014% of cases.^111^

## 8.12. Basic Protocol for Vascular Ultrasound with Microbubble Contrast Agents1,16

After determining the indication for the use of a microbuble contrast agent in VUS, the mandatory protocol described below must be followed.

– Repeat and record standard VUS examination of the target organ.
– Secure venous access for injection of contrast solution with microbubbles (preferably peripheral vein puncture).
– Prepare the solution with the microbubble contrast agent (SonoVue®) following the manufacturer’s instructions.
– Activate the dedicated USEA mode in the machine; if there is no specific software, adjust the MI (< 0.6 and as close as possible to 0.1), image gain (darken the background), and choose the appropriate windows to reduce the depth of the target organ under study.
– Administer the contrast solution, make adjustments to reduce excessive enhancement, and record digital images (video clips) for 10 to 40 seconds after the initial bolus injection; in longer examinations (5 to 8 minutes), record only the necessary parts for later analysis.

Examination with microbubble contrast agents is fundamentally dynamic, and the duration of the examination is short because the microbubbles are rapidly ruptured by US waves, even when using a very low MI setting. Thus, recording video clips of the examination is essential for later processing and careful review of images, ensuring the correct diagnosis and permanent storage of test results.

The main limitations of USEAs in VUS are the examiner’s inexperience, the lack of specific software, difficult access to USEAs in the public health system, and the complete absence of an ultrasound “window”. Clinical contraindications include MI, severe chronic obstructive pulmonary disease, severe cardiac arrhythmias, and hypersensitivity to USEAs (rare).^1,16,111^

## 9. Evaluation of Atheromatous Disease in Vertebral Arteries

### 9.1. Introduction

The investigation of atherosclerotic involvement of the extracranial vertebral artery using VUS is interwoven with the imaging of the carotid arteries. This is essential for the diagnosis and treatment of severe carotid lesions, as well as for a careful assessment of the risks of the surgical approach. Approximately 25% of ischemic strokes involve the posterior circulation, and atherosclerotic disease corresponds to 20% of cases.^118^ Atherosclerotic plaques are predominantly located at the origin of the vertebral arteries, and in most cases they are extensions from the subclavian arteries.^119^ The presence of vertebrobasilar stenosis in the setting of stroke or TIA involving the posterior circulation increases the risk of recurrence by approximately 33% in the first month after the initial event.^120,121^

A detailed description of the anatomy of the vertebrobasilar system arteries can be found in the 2015 DIC Guideline that precedes this update.^1^

### 9.2. Ultrasound evaluation of vertebral arteries

With the technical resources currently available, it is possible to image the entire vertebral artery, including the intracranial segment and the proximal basilar artery. We recommend including the origin of the vessel (most common site of stenosis) and the other extracranial segments in routine evaluation.

### 9.3. Methodology of Routine Examination

Patient positioning is the same as for imaging of the carotid arteries. Depth of field may vary according to neck anatomy. The color scale should be reduced, and the sensitivity of color flow detection should be increase.

The complete methodology is described in the 2015 DIC Guideline.^1^

### 9.4. Normal Parameters

The anatomical and hemodynamic parameters of vertebral artery hypoplasia are described in the 2015 Gideline^1^ and shown in Table 5.

**Table 5:** – Anatomical and hemodynamic parameters for the definition of vertebral artery hypoplasia

Anatomical and hemodynamic criteria for vertebral hypoplasia
Diameter ≤ 2mm in segment V2
Decreased diastolic flow
Resistance index > 0.75
Increased caliber of the contralateral vertebral artery (> 4 mm) at normal velocities

### 9.5. Stenosis Quantification

#### 9.5.1. Proximal Stenosis (V0-V1)

The diagnosis of proximal stenoses is based on the identification of turbulence on color Doppler and an increase in flow velocities at the lesion site (which is not always visualized). In tortuous vertebral arteries, there may be a physiological increase in velocities. A dampened waveform pattern corroborates the presence of significant proximal stenosis. If the 2D image is high quality, it is possible to detect a narrowing of the vessel lumen and measure, using power angiography, the residual lumen according to the distal anatomical criterion.

We recommend using the cutoff values in Table 6, adapted from the study by Hua et al.,^122^ to define the degrees of proximal stenosis on the vertebral artery. PSV at the origin of the vertebral artery is the most specific parameter for quantification of proximal vertebral stenosis when compared with other spectral criteria, such as the peak velocity index and EDV.

**Table 6:** – Velocity cutoff values for the evaluation of proximal stenosis in the vertebral artery (adapted from Hua et al.**122**)

Stenosis	< 50%	50-69%	70-99%
Vmax	≥ 85 cm/s	≥ 140 cm/s	≥ 210 cm/s
PVI*	≥ 1.3	≥ 2.1	≥ 4
EDV	≥ 27 cm/s	≥ 35 cm/s	≥ 55 cm/s

*PV: peak velocity index in the stenosis and V2 segment.

#### 9.5.2. Vertebral Stenosis in the Remaining Segments (V2-V4)

The diagnosis of stenosis in the remaining segments is based on a combined analysis of turbulence on color Doppler, local increase in flow velocities, increase in velocity indices, and damping in distal flow, as there are no quantification tables for stenoses in the V2-V4 segments.^123^

For segments that cannot be visualized on conventional examination, such as the intracranial segment (V4), findings are indirect and correlate with the degree of stenosis and the origin of the posterior inferior cerebellar artery (PICA). Spectral curves of stenoses before the origin of the PICA show reduced velocities and an elevated resistance pattern on segments V1-V2, whereas stenoses after the origin of the PICA do not cause flow alterations, as there is deviation to the cerebellum. In these cases, transcranial Doppler (TCD) imaging is essential to confirm the diagnosis.

#### 9.5.3. Vertebral Artery Occlusion

Findings vary according to the level of occlusion. Chart 8 shows possible spectral curves according to the level of occlusion. It is not uncommon for a vertebral artery occluded at its origin can return to its distal segment through well-defined anastomotic circuits. This possibility should be investigated through imaging of the distal extracranial segments.

**Chart 8:** – Spectral curves according to the degree of occlusion of the vertebral artery. PICA: posterior inferior cerebellar artery.

## 9.6. Subclavian Steal Syndrome

A subclavian steal effect can arise from hemodynamically significant stenosis or occlusion of the brachiocephalic trunk or proximal segment of the subclavian artery (right or left): if the caliber of the ipsilateral vertebral artery is normal and there is no associated significant atheromatous disease, blood supply of the affected subclavian artery is maintained through a steal effect from the contralateral side.^118,124-126^ In this case, the subclavian steal may be detected through the evaluation of spectral waveform morphology and flow direction in the vertebral artery on the same side as the abnormal subclavian artery, at rest or after induction of reactive hyperemia (compression of the ipsilateral arm with a blood pressure cuff). Unlike distal vertebral stenoses, in which the first component to be affected is the diastolic one, the earliest manifestation of the subclavian steal syndrome is a mild deceleration of blood flow during the systolic phase (almost imperceptible for less experienced examiners).

The classification of different spectral curve morphologies observed in vertebral arteries is described in Chart 9. In general, the type of steal correlates with greater degrees of stenosis in the subclavian artery or brachiocephalic trunk. In the case of severe stenosis in the brachiocephalic trunk, there may be concomitant carotid steal, which will present a spectral curve with systolic flow inversion.

**Chart 9:** – Types of steal according to spectral curve pattern.

## 10. Transcranial Doppler in Extracranial Carotid and Vertebral Atherosclerotic Disease

The fundamental aim of TCD in patients with symptomatic or asymptomatic extracranial carotid and vertebral atherosclerotic disease is to investigate the predictive value of ischemic stroke occurrence.

TCD offers some valuable tools, including the a) detection of spontaneous cerebral microemboli and b) recording of hemodynamic information during intraoperative monitoring (endarterectomy) and during endovascular procedures.^127-129^

### 10.1. Imaging Techniques

The imaging technique depends on the clinical indication. In outpatient and intraoperative evaluations, the need for continuous and long-term monitoring requires specific equipment, including an adjustable headset for fixing the probe. This will ensure that all necessary information is recorded during the transient event to define the most appropriate therapeutic approach.^129^

TCD devices are “blind” due to the absence of 2D imaging and color flow mapping (CFM), which means that useful anatomical information is lost during the examination. However, by providing a headset for probe fixation, these devices allow for continuous flow monitoring.

A standard conventional TCD examination should be initially performed, with the aim of evaluating the vascular anatomy and detecting any possible collateral flow.^130,131^ In addition, *in situ* investigation of the site of intracranial segmental intravascular stenosis can be performed, which is present in 10% of cases of ischemic stroke.^134,135^

The standard “blinded” TCD examination consists of insonating the segments of all arteries: anterior circulation, including the right and left internal carotid arteries and their branches; and posterior circulation, including the basilar artery (which arises from the confluence of the right and left vertebral arteries) and its branches.^136,137^

Both anterior and posterior circulations are connected by communicating arteries (left and right anterior and posterior), integrating a system of arteries known as the circle of Willis (Figure 9). This vascular architecture is an efficient automatic collateral mechanism of collateralization in case of occlusion in any of the vessels, preventing or mitigating the consequences of cerebral ischemia. However, anatomical variations are present in more than 50% of cases, which explains why different people suffer different sequelae from occlusions in the same artery. In each cerebral hemisphere of the anterior circulation, the ICA first gives a branch to the ophthalmic artery and then gives rise to the anterior and middle cerebral arteries, which supply most of the brain. In the posterior circulation, the right and left vertebral arteries merge into the basilar artery, which is divided into the right and left posterior cerebral arteries, supplying the brainstem and the cerebellar region.

**Figura 9:** – Schematic representation of the circle of Willis (9A) and contrast-enhanced ultrasound image in the right temporal window (9B).

A transducer with a frequency ≤ 2 MHz must be used in TCD examinations, as the deep location of the intracranial arteries requires the use of low-frequency waves for visualization. Identification of the insonated vessel by “blinded” Doppler depends on the a) acoustic window used; b) position of the transducer in relation to the skull (angle of incidence); c) depth of “sample volume”; d) characteristics of waveform spectral curves (morphology, flow direction in relation to the transducer, PSV, EDV, mean velocity, and pulsatility and resistance indices). The parameters on Table 7 can be used to characterize the examined artery, except in cases of anatomical variations, for which duplex image are more advantageous.

**Table 7:** – Criteria for the identification of intracranial vessels

Artery	Window	Transducer angle in relation to the skull	Vessel depth	Mean flow velocity	Flow direction
Intrasellar carotid siphon	Orbital	Perpendicular	55-70 mm	40-50 cm/s	Negative
Geniculate carotid siphon	Orbital	Perpendicular	55-70 mm	40-50 cm/s	Negative positive
Suprasellar carotid siphon	Orbital	Perpendicular	55-70 mm	40-50 cm/s	Positive
Ophthalmic	Orbital	Perpendicular	40-60 mm	20 cm/s	Positive
Distal internal carotid	Temporal	Downward	55-70 mm	45 cm/s	Positive
Anterior cerebral	Temporal	Upward and anterior	60-70 mm	60 cm/s	Negative
Middle cerebral	Temporal	Perpendicular	35-60 mm	70 cm/s	Positive
Posterior cerebral (p1)	Temporal	Downward and posterior	55-70 mm	40 cm/s	Positive
Posterior cerebral (p2)	Temporal	Downward and posterior	55-70 mm	40 cm/s	Negative
Vertebral (V4)	Foraminal	Slightly upward and lateral	55-70 mm	40 cm/s	Negative
Proximal basilar	Foraminal	Slightly upward and central	70-120 mm	45 cm/s	Negative
Inferior posterior cerebral	Foraminal	Slightly upward and lateral	40-55 mm	45 cm/s	Positive

### 10.2. Standard Protocol for a Conventional “Blinded” Transcranial Doppler Examination

Position the patient in supine and gently place the 2-MHz transducer over each of the five classic acoustic windows, in no specific order, to ensure imaging of all intracranial arteries: a) transorbital (right and left); b) transtemporal (right and left); c) transforaminal.

**a) Transorbital windows:** image the ophthalmic arteries and carotid siphons (cavernous portions of internal carotid arteries). The transducer should be placed over the eye, with the patient with the eye closed, without applying local pressure (Figure 10).
  **Figure 10:** – Transcranial Doppler in the transorbital window (10A); schematic representation of insonated arteries (carotid siphon and ophthalmic – 10B).
**b) Transtemporal windows:** located above the zygomatic arch (approximately 1 cm away from the external auditory canal), they vary individually in length and quality. The transducer should be initially placed perpendicular to the skull and then subtly tilted anteriorly and posteriorly to obtain images from the ipsilateral distal internal carotid, anterior cerebral (A1), middle cerebral (M1), top of the basilar, and posterior cerebral (P1 and P2) arteries (Figure 11). The communicating arteries (anterior and posterior) can also be insonated through these ultrasonic windows.
  **Figure 11:** – Transcranial Doppler in the transtemporal window (11A); schematic representations of the anterior (11B), middle (11C), and posterior (11D) cerebral arteries.
**c) Transforaminal window:** only access to the lumens of the intracranial segments of the vertebral arteries (V4) and to the origin of the basilar artery (Figure 12),
  **Figure 12:** – Transcranial Doppler in the foraminal window (12A); schematic representation of the basilar and vertebral arteries (12B). RV: right vertebral; LV: left vertebral.
in addition to the posteroinferior cerebellar arteries (crucial branches for collateral flow route in cases of occlusion of the vertebral artery above their emergence). The patient should be positioned in lateral decubitus, with the chin touching the thorax to expose the occipital region (topography of the foramen magnum), or sitting in the bed or a chair to facilitate positioning of the examiner. Pulsed Doppler will show the flow moving away from the transducer in the vertebral and basilar lumens; in the PICAs, flow direction is reversed.

Spectral waveforms recorded in the intracranial arteries have similar morphology, differing only in the specific velocities of each vessel and in the direction in relation to the transducer. Low frequency should be used in all segments except the ophthalmic, which is the only artery with a high resistance index—although it branches off the ICA, it supplies extracranial structures.

### 10.3. Standard Protocol for Continuous Transcranial Doppler

The patient should wear an adjustable headset with 2 or more “blind” transducers fixed to it and placed over the temporal windows, directed towards the middle cerebral arteries (Figure 13). Continuous and simultaneous flow analysis in both arteries ensures real-time observation and recording of emboli occurrence, as well as count of events per hour. Microembolism is reflected by a transient, short-term (less than 300 ms), high-intensity signal, depicted on the spectral curve (pulsed Doppler) as a vertical trace associated with an audible output, called high-intensity transient signal (HITS) (Figure 14). Counting the number of HITSs and differentiating between solid and gas HITS is important in CEA and endovascular procedures. The risk of ischemic stroke depends on the intensity of the embolic phenomena and can be estimated according to the number of HITS registered with the TCD.^138,139^

**Figure 13:** – Adjustable headset for transducer fixation and continuous flow monitoring in middle cerebral arteries. TCD: transcranial Doppler.

**Figure 14:** – High-intensity transient signals indicating emboli (gas or solid).

### 10.4. Clinical Usefulness of Transcranial Doppler in Cervical Atherosclerotic Disease

#### 10.4.1. Identification of Patients with HITS

Microembolism distal to carotid stenosis indicates a 7.5 times greater risk of recurrent ischemic stroke or TIA.^140^ In patients with recent symptomatic carotid stenosis (less than 7 days), the risk of recurrent ischemic stroke is 26% at 30 days.^140^ Therefore, microembolism screening can support the intensification of antithrombotic therapy, as shown in the Clopidogrel and Aspirin for Reduction of Emboli in Symptomatic Carotid Stenosis^141^ and Clopidogrel plus Aspirin for Infarction Reduction^142^ studies, or anticipate endarterectomy and endovascular treatment.

The identification of HITSs is also useful in the risk stratification of patients with asymptomatic carotid stenosis. The Asymptomatic Carotid Emboli Study identified an annual risk of ischemic stroke or TIA ipsilateral to the stenosis of 7.1% in patients with HITSs and 3.0% in those without microembolism.^143^

Microembolism was detected in 49% of patients in the first 24 hours after the onset of ischemic stroke, and this rate progressively and significantly decrease after 48 hours.^144-147^ Likewise, in patients with TIA, the presence of HITS is associated with the occurrence of ischemic stroke or new TIA.^147^

During endarterectomy, real-time detection of emboli released in the carotid occlusion phase of atherosclerotic plaque resection can be easily and quickly performed with continuous TCD monitoring, ensuring greater safety during the procedure and reducing postoperative ischemic complications.

#### 10.4.2. Induced Hemodynamic Repercussions

Special attention should be given to the evaluation of intracranial hemodynamic repercussions induced during flow monitoring of the middle cerebral arteries, which should be performed with a TCD device with a headset. In symptomatic patients, monitoring should last for at least 1 hour; in asymptomatic patients, monitoring should be extended to 4 hours in order to obtain better accuracy. The analysis of cerebral autoregulation and cerebral vasomotor reserve (CVR) provides some of the most useful information.

Cerebral autoregulation (or autoregulatory pressure) is a mechanism that maintains cerebral blood flow relatively constant despite changes in cerebral perfusion pressure (CPP).

Factors affecting cerebral perfusion include CPP and cerebrovascular resistance (microcirculation). Cerebral blood flow can remain constant despite variations in mean arterial pressure (MAP) if compensatory changes occur in the microcirculation (arterioles). There are two methods for assessing the state of cerebral autoregulation: a static and a dynamic one. TCD is one of the most used methods for estimating changes in cerebral perfusion. Dynamic autoregulation translates transient changes in cerebral blood flow after rapid changes in blood pressure and can be provoked by the femoral cuff test: a blood pressure cuff placed on the patient’s thighs is inflated and then abruptly deflated with the aim of inducing hyperemia in the legs and a drop in systemic blood pressure. Cerebral autoregulation will ensure that hypotension does not alter the cerebral blood flow.^148^

CVR can be estimated through the cerebrovascular reactivity test, whose objective is to quantify the dilation capacity of a certain arterial sites, identifying patients with hemodynamically critical stenoses and at high risk of cerebral circulation insufficiency.^149^ Among the tests for the evaluation of microcirculatory reserve, CO_2_ inhalation consists of inhaling in a controlled manner a gas mixture enriched with CO_2_.^150^ Hypercapnia causes dilation of arterioles and increased blood flow (Figure 15), whereas hypocapnia promotes vasoconstriction and reduced cerebral blood flow. During monitoring of blood flow velocity in the middle cerebral artery, the velocity can be reduced to 50% below baseline values during hypocapnia, while in hypercapnia it can rise up to 200% above baseline values. As a clinical cutoff point, it is recommended that flow increases of less than 10% should be considered CVR impairment.

**Figure 15:** – TCerebral vasomotor reserve capacity assessment through the CO_*2*_ inhalation test; significant reductions in velocities and the resistance index (15B) were observed after CO_*2*_ inhalation (15A).

Intraoperative flow monitoring of the middle cerebral arteries with TCD allows the analysis of variations in blood flow velocities in response to the use of volatile anesthetics, which cause vasodilation of the cerebral microcirculation and increase cerebral blood flow, and hypnotic agents, which decrease cerebral blood flow.^151^

Hyperperfusion syndrome: in the immediate postoperative period of CEA in patients with severe stenosis, the cerebral bed of small vessels (pial arteries and arterioles) may present with chronic vasodilation and loss of vasoconstriction capacity after sudden restoration of perfusion by CEA. This will lead to inadequate cerebral hyperemia once normal pressure is introduced into the vasodilator tissue bed, and significant morbidity associated with edema, intracranial hypertension, and hemorrhage may occur.^152-155^ Such a mechanism has also been described immediately or up to 24 to 48 hours after resection of arteriovenous malformations. TCD can detect spectral curves with increased velocities and low pulsatility and resistance in cerebral vessels. Flow velocity measurements in the middle cerebral arteries can guide treatment until normalization.

#### 10.4.3. Evaluation of Intracranial Vertebral Stenosis (V4)

Routine imaging of vertebral arteries should not be restricted to the extracranial segments, as plaques with severe stenosis, or even occlusions in intracranial segments (V4) of the vertebrobasilar system, may not cause any abnormality in spectral flow curves in cervical topography (V0-V3). If the atherosclerotic plaque is located before the origin of the PICA in the intracranial spine, the spectral curves will show low amplitude and high resistance in ipsilateral V1-V3. If the stenotic lesion or lumen occlusion is located above the PICA, there may be flow deviation to the cerebellum, and the spectral curves will be normal, which makes the TCD a valuable diagnostic tool (Figure 16).

**Figure 16:** – Anatomy of the intracranial segments of the vertebral and basilar arteries and their branches (16A); foraminal window: color flow mapping in vertebral lumens (V4) and PICAs (16B); color mapping of transition from vertebral V3 (extra) to V4 (intracranial – 16C); spectral curve on pulsed Doppler moving away from the transducer (V4) (16D); color flow mapping of the left PICA (flow towards the transducer – 16E); and spectral curves on pulsed Doppler of the left PICA (16F).PICA: posterior inferior cerebellar artery

Imaging of the intracranial segments of the vertebrobasilar system requires a ≤ 2.0-MHz sectoral transducer with CFM. Through the foramen magnum, the US will reach the arteries and provide visualization of the intraluminal flow, defining the regional anatomy. Vertebral artery flow moves away from the transducer and, in the PICA, has the opposite direction, facilitating vessel identification.

## 10.5. Recommendations

Our recommendations on the use of TCD in carotid atherosclerotic disease are summarized in Chart 4.

1. In patients with extracranial carotid and vertebral atherosclerotic disease, silent microembolism should be investigated with a “blinded” TCD device with a headset for transducer fixation. Continuous flow monitoring in the middle and basilar cerebral arteries should be performed for at least 4 consecutive hours.
2. Pre-CEA assessment of CVR provides valuable information for reducing the risk of severe cerebral ischemia during surgery.
3. Perioperative monitoring and for at least 90 minutes immediately after CEA is essential for simultaneous diagnosis and early treatment of complications resulting from gas or solid embolization (pieces of atherosclerotic plaques or thrombi).
4. We recommend including imaging of the intracranial segments of the vertebral and basilar arteries (via the foraminal window) in routine examinations of carotid and cervical vertebral arteries of symptomatic patients without extracranial anatomical lesions that warrant clinical attention.
